# Combined LM and SEM study of the middle Miocene (Sarmatian) palynoflora from the Lavanttal Basin, Austria: Part V. Magnoliophyta 3 – Myrtales to Ericales

**DOI:** 10.1080/00173134.2019.1696400

**Published:** 2020-02-14

**Authors:** Friđgeir Grímsson, Johannes M. Bouchal, Alexandros Xafis, Reinhard Zetter

**Affiliations:** 1 Department of Botany and Biodiversity Research, University of Vienna, Vienna, Austria; 2 Department of Palaeobiology, Swedish Museum of Natural History, Stockholm, Sweden; 3 Department of Palaeontology, University of Vienna, Vienna, Austria

**Keywords:** angiosperms, Cainozoic, Carinthia, fossil pollen, eudicots, palaeoclimate, palaeovegetation

## Abstract

The continued investigation of the middle Miocene palynoflora from the Lavanttal Basin reveals numerous additional angiosperm taxa. The Myrtales to Ericales pollen record documented here comprises 46 different taxa belonging to Onagraceae (*Ludwigia*), Ericaceae (*Craigia, Reevesia, Tilia*), Anacardiaceae (*Pistacia*), Rutaceae (*Zanthoxylum*), Sapindaceae (*Acer*), Santalaceae (*Arceuthobium*), Amaranthaceae, Caryophyllaceae, Polygonaceae (*Persicaria, Rumex*), Cornaceae (*Alangium, Cornus, Nyssa*), Ebenaceae (*Diospyros*), Ericaceae (*Andromeda, Arbutus, Empetrum, Erica*), Sapotaceae (*Pouteria, Sideroxylon*), Styracaceae (*Rehderodendron*) and Symplocaceae (*Symplocos*). Köppen signatures of potential modern analogues of the additional fossil woody elements confirm the hypothesis of a subtropical (*Cfa, Cwa*) climate at lower elevations and subsequent transition into a temperate climate with altitudinal succession (*Cfa* → *Cfb*/*Dfa* → *Dfb; Cwa* → *Cwb* → *Dwb*-climate). The fossil plants represent different vegetation units, from wetland lowlands to well-drained montane forests. Many of the fossil taxa have potential modern analogues that can be classified as nemoral and/or meridio-nemoral and/or semihumid-meridional vegetation elements. New is the recognition of oreotropical elements, which are direct indicators for a substantial altitudinal gradient.

This contribution is the fifth in a series of articles on the middle Miocene (Sarmatian) palynoflora from the Lavanttal Basin, Austria. For a general introduction and non-angiosperm pollen record, see Grímsson et al. (2011; spores, gnetophytes, ginkgophytes); Grímsson and Zetter (2011) covers the remaining gymnosperms (conifers), and Grímsson et al. (2015a, 2016a) the Magnoliales to Rosales. First numerical palaeoclimate and palaeovegetation assessments are provided in Grímsson et al. (2016), with focus on the, therein described, angiosperm clades of Fagales and Rosales. Using ‘Köppen signatures’ of potential modern analogues and classifying them as ‘Schroeder categories’ (Denk et al. 2013), we inferred a subtropical, per- to winter-humid *Cfa/Cwa* climate (Köppen 1936; Kottek et al. 2006; Rubel et al. 2017) in the lowlands providing habitats for meridio-nemoral and accessory nemoral elements. The occurrence of boreal elements, not adapted to subtropical climates, hinted towards a significant altitudinal gradient. Here, we collect the fossil pollen of two further angiosperm clades, the malvids and asterids. The malvids are represented by five orders, the Myrtales, Malvales, Sapindales, Santalales and Caryophyllales, accounting for 21 of the here recorded taxa. The asterids are represented by two orders, the Cornales and Ericales, in total, 25 taxa. Based on the potential modern analogues of the fossils we can confirm and refine our preliminary hypotheses about palaeovegetation and palaeoecology.

## Material and methods

For a detailed account on the geographical position, geology and age of the Lavanttal Basin and its surroundings, sedimentology and palaeoenvironment, preservation of organic matter, as well as detailed information on the sediment samples and preparation methods see Grímsson et al. (2011).

The fossil pollen grains were investigated both by light microscopy (LM) and scanning electron microscopy (SEM) using the single-grain method as described by Zetter (1989) and Halbritter et al. (2018). Pollen micrographs in compiled figures show the same single pollen grain photographed with LM (sometimes rotated and/or at different focus level) and with SEM (overview and close-up of sculpture).

**Figure 1.**
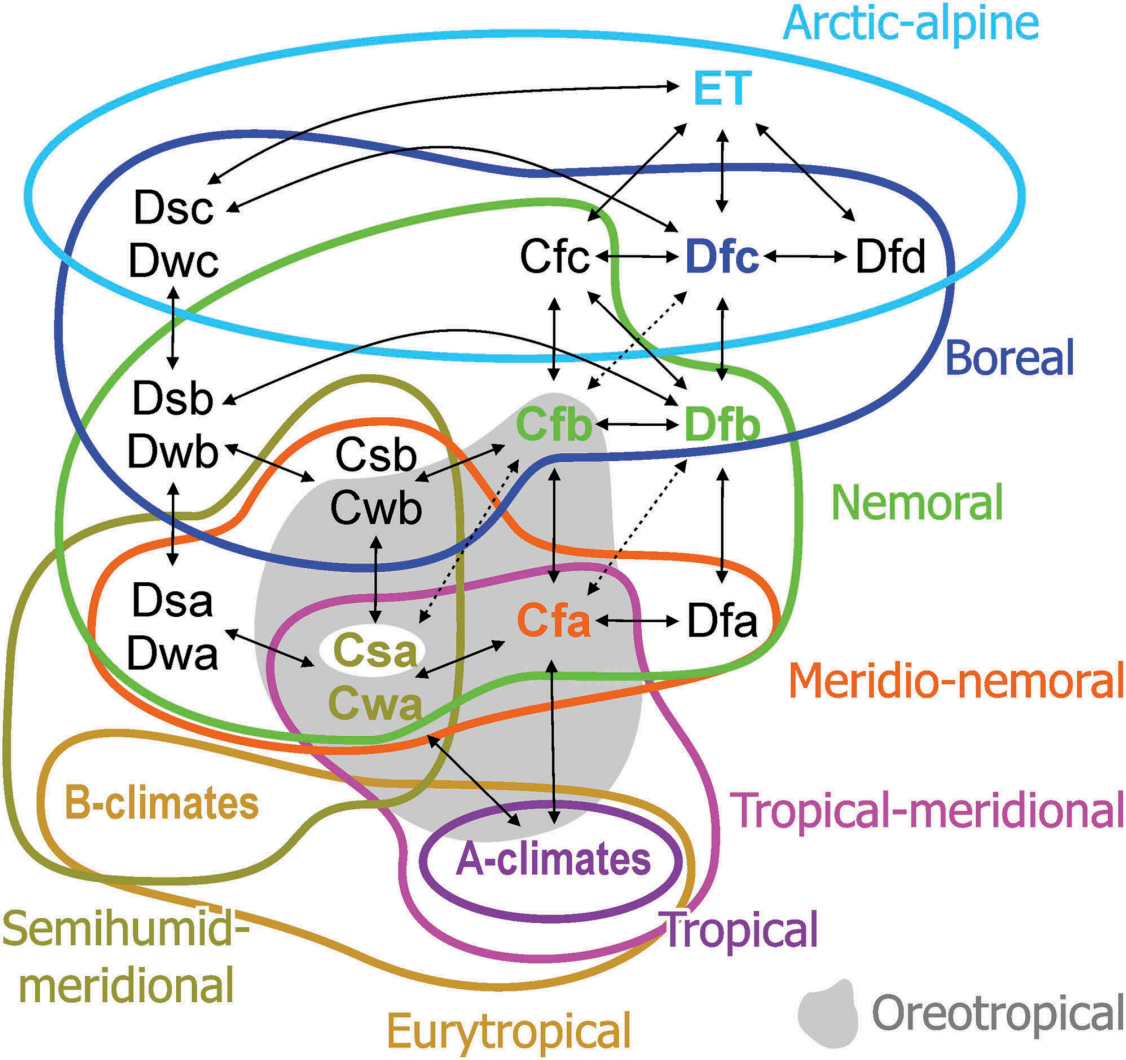
Circumscription of vegetation elements by Köppen climate types (‘Köppen signatures’), introducing one new category (scheme modified after Denk et al. 2013; Grímsson et al. 2016).

We used ‘Köppen signatures’ (Denk et al. 2013) to summarise the climatic niche occupied by potential modern analogues (species groups, genera) of the determined pollen taxa. We also categorised the potential modern analogues as climate-dependent vegetation elements adapting the concepts of Schroeder (1998) to provide a more generalised account of the palaeovegetation in relation to modern-day vegetation zones (cf. Denk et al. 2013, figure 2; Grímsson et al. 2016, figure 2). Here, we recognise two additional climate–vegetation categories, the ‘oreotropic’ (Figure 1) and ‘austral’ elements (Schroeder 1998) to accommodate species with hygric and thermic preferences not covered by the data set used by Denk et al. (2013) and Grímsson et al. (2016). Oreotropic species (Schröder 1998) are found in fully humid temperate to winter-dry temperate climates ([*A*], *Cfa, Cfb, Cwa, Cwb; sensu* Köppen-Geiger in Kottek et al. 2006) along altitudinal thermic successions in low latitudes characterised by tropical climates. Climatically they are hence similar to meridio-nemoral elements, but are exclusively found in mountainous areas within the tropics and, in contrast to meridio-nemoral elements, show no physical latitudinal connection to the nemoral or boreal vegetation zones. Austral species are adapted to thermic and humidity conditions of the Southern Hemisphere similar to the meridional and nemoral zones of the Northern Hemisphere including temperate fully humid to summer- and winter-dry climates with hot to warm summers (*Cfa, Cfb, Csa, Csb, Cwa, Cwb; sensu* Köppen-Geiger in Kottek et al. 2006). The climate data for all potential modern analogues of the fossil taxa presented herein are listed in Supplementary File S1.

Note that we use here two, semantically partly overlapping concepts that have caused much confusion in palaeoclimatic and palaeovegetational literature when not strictly applied. For the general climate/vegetation bands/zones, we make use of the latitudinal-based modification of Köppens climate classification by Trewartha (1954; Trewartha & Horn 1980), which recognises a subtropical zone (addressed also as ‘subtropics’) between the equatorial tropical zone (‘tropics’) and the (fully or ‘cool’) temperate zone at mid-latitudes (*c*. 30°–60°). The Köppen signatures refer strictly to Köppen’s system that has no explicit subtropical zone. Thus, when we speak of ‘warm temperate climates’ (Köppen’s *C-*climates), this includes climates typical for the (low- to mid-altitude) subtropics with hot and/or dry summers (*Cfa, Cwa, Csa, Csb* p.p.) but also climates typical for the warmer part of the temperate zone (*Cfb, Cwb*, rest of *Csb*) and climates that, according Trevartha’s system, transition into the boreal (also known as ‘cold temperate’) zone (*Cfc, Cwc*). Vegetation-wise, Köppen’s original system is more applicable since many dominant and common taxa of the subtropics extend into the warmer part or deep into the temperate zone. The transition from the subtropical into the temperate zone is vegetation-wise hardly visible in North America or East Asia due to the absence of major east–west physical barriers.

## Systematic palaeontology

All descriptions of angiosperm pollen presented herein include the most diagnostic features observed both in LM and SEM. The pollen terminology follows mostly Punt et al. (2007, LM) and Halbritter et al. (2018, SEM). The classification of orders and families follow APG III (2009). Families and genera are arranged in alphabetical order.

Clade Malvids

Order Myrtales Juss. ex Bercht. et J.Presl

Family Onagraceae Juss.

*Genus* Ludwigia *L.*

Ludwigia *sp. 1*

(*Figure 2A–E*)

**Figure 2.**
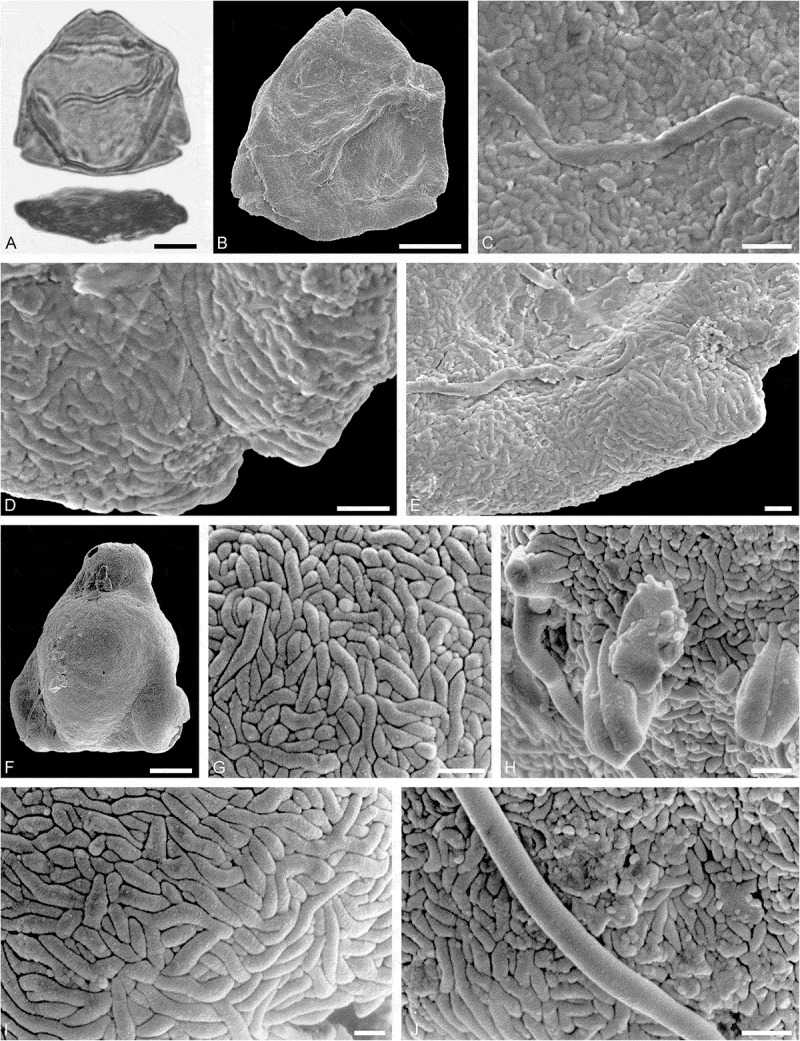
Light microscopy (LM) (**A**) and scanning electron microscopy (SEM) (**B–J**) micrographs of dispersed fossil Onagraceae pollen. **A–E.** *Ludwigia* sp. 1. **C.** Close-up showing part of a smooth viscin thread. **D.** Close-up of sculpture around aperture, elongated rugulae. **E.** Close-up of rugulae in area of mesocolpium. **F–J.** *Ludwigia* sp. 2. **G.** Close-up of rugulae in polar area. **H.** Close-up of viscin threads attachment points. **I.** Close-up of rugulae in area of mesocolpium. **J.** Close-up showing part of a smooth viscin thread. Scale bars 10 µm (A, B, F), 1 µm (C–E, G–J).

### 

#### 

##### Description

Pollen, monad, oblate, outline triangular in polar view, elliptic in equatorial view; equatorial diameter 38–41 µm wide in LM, 37–38 µm wide in SEM; tricolporate; exine 0.9–1.2 µm thick (LM), nexine thinner than sexine; tectate; sculpture scabrate in LM, microrugulate to rugulate in SEM, rugulae elongated around pori, viscin threads originating on proximal face of pollen, viscin threads smooth, mostly 0.4–0.6 µm wide (SEM).

##### Remarks

The pollen morphology of Onagraceae has been studied in detail using LM, SEM and transmission electron microscopy (TEM) by Ting (1966), Brown (1967), Skvarla et al. (1976, 1978), Praglowski et al. (1983, 1987, 1988, 1994), Patel et al. (1984), Keri and Zetter (1992), Punt et al. (2003), and Makbul et al. (2008). These studies show that Onagraceae produce distinct pollen types that cannot be confused with pollen from any other angiosperm family (cf. Patel et al. 1984), making it also possible to identify fossil Onagraceae pollen/tetrads at generic level.

##### Fossil record

The macrofossil record of *Ludwigia* is mostly confined to Europe, including the Oligocene to Pliocene of Germany (Mai 1985, 1989, 1997, 1998, 2000, 2001; Mai & Walther 1988; Mai & Wähnert 2000), the Miocene to Pliocene of Poland, the Czech Republic, and Slovakia (Buzek et al. 1985; Knobloch 1988; Zastawniak 1992), the Miocene of Denmark (Friis 1985), and the Pliocene of Italy (Mai 1995a). Fossil pollen grains assigned to *Ludwigia* are frequent compared to other genera of Onagraceae. Fossil *Ludwigia* pollen has been identified in the Upper Cretaceous of India (Farooqui et al. 2019), the Paleocene, Eocene and Miocene of China (Song et al. 2004; Grímsson et al. 2012b), the Eocene to Oligocene of Canada (Rouse 1962, 1977; Piel 1971), the Eocene of Columbia (Gonzáles Guzmán 1967) and Russian Far East (Brattseva 1969), the Oligocene to Pliocene of the United States (Traverse 1955; Rachele 1976), the Miocene of Mexico (Graham 1976a, 1976b, 1987) and Turkey (Bouchal et al. 2016b), and the Miocene/Pliocene of Guatemala (Graham 1998). Several Paleocene to Pliocene fossil Onagraceae pollen that are thought to have botanical affinities to *Ludwigia* have also been assigned to the form-genera *Jussitriporites* Gonzáles-Guzmán and *Corsinipollenites* Nakoman (e.g. Gonzáles Guzmán 1967; Krutzsch 1968, 1970c; Frederiksen 1983; Quattrocchio & Volkheimer 1990; Quattrocchio et al. 1997; Zheng et al. 1999; Stuchlik et al. 2009).

##### Ecological implications

*Ludwigia* displays a cosmopolitan, but mainly pantropical, distribution with some of its 82 species occurring on every continent except Antarctica. These are mostly small herbaceous annual or perennial plants (Wagner et al. 2007). Most extant *Ludwigia* species are water plants. It is very likely that both *Ludwigia* sp. 1 and *Ludwigia* sp. 2 represent small herbaceous plants that grew in lakes or along their shorelines in the lowland wetlands of the Lavanttal Basin.

Ludwigia *sp. 2*

(*Figure 2F–J*)

##### Description

Pollen, monad, oblate, outline triangular in polar view, elliptic in equatorial view; equatorial diameter 52–56 µm wide in LM, 50–52 µm wide in SEM; triporate, pori circular to elliptic; exine 1.0–1.3 µm thick (LM), nexine thinner than sexine; tectate; sculpture scabrate in LM, rugulate in SEM, rugulae elongated around pori, viscin threads originating on proximal face of pollen, attachment points are thickened and fused by three or more elongated (protruding) rugulae elements, viscin threads smooth, mostly 0.6–0.8 µm wide (SEM).

##### Remarks

The *Ludwigia* sp. 2 is much larger than the *Ludwigia* sp. 1, it is also triporate versus tricolporate in *Ludwigia* sp. 1. Attachment points of viscin threads are also markedly thickened and fused by rugulate elements in *Ludwigia* sp. 2, a feature not observed in *Ludwigia* sp.1.

Order Malvales Juss. ex Bercht. et J.Presl

Family Malvaceae Juss.

*Genus* Craigia *W.W.Sm. et W.E.Evans*

Craigia *sp.*

(*Figure 3A–F*)

**Figure 3.**
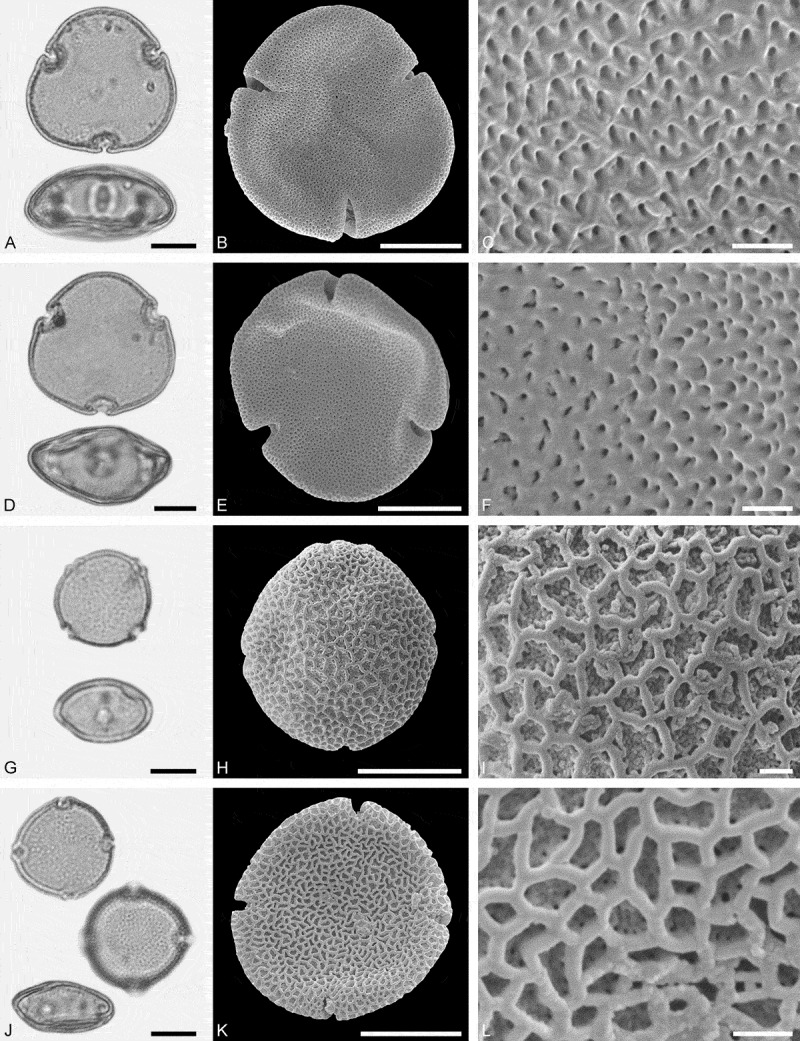
Light microscopy (LM) (**A, D, G, J**) and scanning electron microscopy (SEM) (**B, C, E, F, H, I, K, L**) micrographs of dispersed fossil Malvaceae pollen. **A–C.** *Craigia* sp., close-up of central part of proximal polar area. **D–F.** *Cragia* sp., close-up of central part of proximal polar area. **G–I.** *Reevesia* sp., close-up of central part of distal polar area. **J–L.** *Reveesia* sp., close-up of central part of proximal polar area. Scale bars 10 µm (A, B, D, E, G, H, J, K), 1 µm (C, F, I, L).

##### Description

Pollen, monad, oblate, convex triangular in polar view, elliptic in equatorial view; polar axis 16–19 µm long in LM, equatorial diameter 32–34 µm wide in SEM, 28–30 µm wide in SEM; brevitricolporate, planaperturate; exine 0.9–1.2 µm thick, nexine thinner than sexine, nexine thickened around endopori, thickening horseshoe-like in outline; sculpture reticulate in LM, microreticulate in SEM, muri narrow and crested on distal polar area, muri slightly microstriate, lumina circular to elliptic in outline, brochi funnel-shaped, proximal polar area microreticulate to perforate (SEM).

##### Remarks

Pollen morphology of *Craigia yunnanensis* W.W. Smith et W.E. Evans has been documented using LM and SEM by Long et al. (1985) and Kvaček et al. (2002). In LM the apertures of *Craigia* pollen is characterised by a circular horseshoe-like nexine thickening best observed in optical cross-section (Kvaček et al. 2002, plate V). In *Tilia*, this thickening is much broader/wider and less convex (Perveen et al. 2004, figure 4). *Tilia* pollen is also larger than the pollen of *Craigia*, and the sculpture of *Tilia* pollen observed with LM is more prominent.

**Figure 4.**
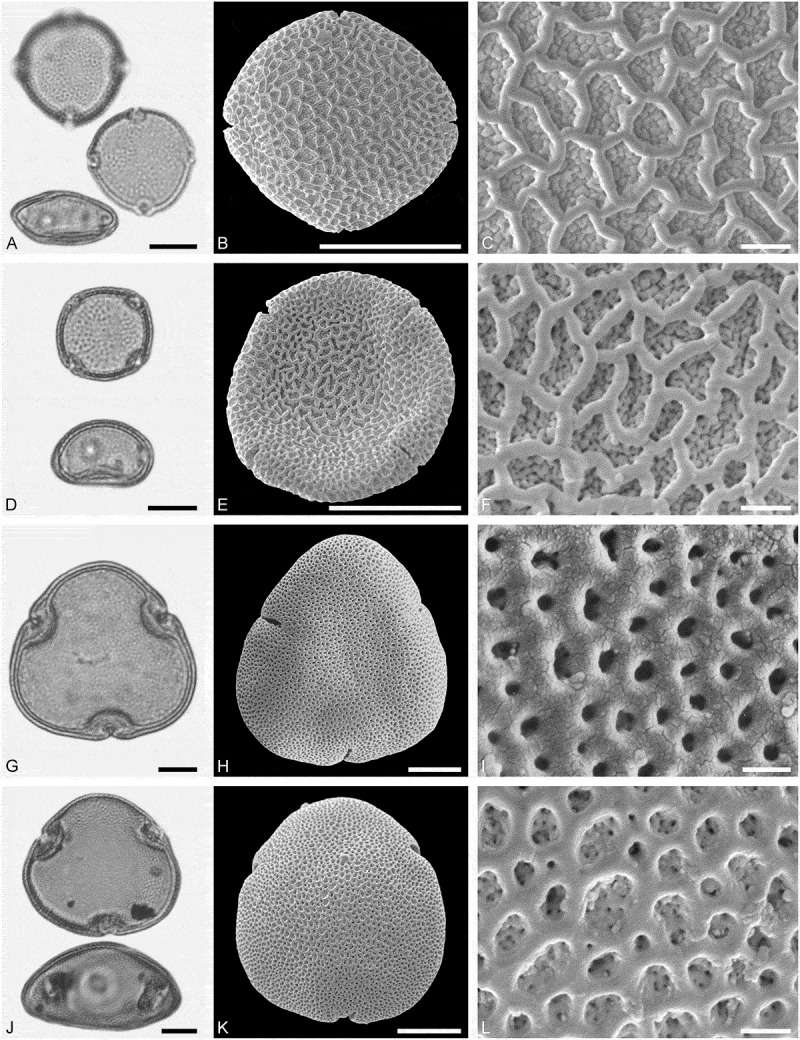
Light microscopy (LM) (**A, D, G, J**) and scanning electron microscopy (SEM) (**B, C, E, F, H, I, K, L**) micrographs of dispersed fossil Malvaceae pollen. **A–C.** *Reveesia* sp., close-up of central part of distal polar area. **D–F.** *Reveesia* sp., close-up of central part of proximal polar area. **G–I.** *Tilia* sp. 1, close-up of central part of proximal polar area. **J–L.** *Tilia* sp. 1, close-up of central part of distal polar area. Scale bars 10 µm (A, B, D, E, G, H, J, K), 1 µm (C, F, I, L).

##### Fossil record

The macrofossil record of *Craigia*, including leaves and fruits, is summarised in Kvaček et al. (2005). The record shows that this genus had a much wider distribution during the Cainozoic, with numerous fossils from the early Oligocene to late Pliocene of Western Eurasia, late Eocene/Oligocene of Spitsbergen, the Paleocene to Miocene of East and Central Asia, and the middle Eocene to middle Miocene of North America (e.g. Kvaček 2004; Kvaček et al. 2005; Jin et al. 2009; Liu et al. 2012). Fossil *Craigia* pollen is also well represented in the palynological record of the Cainozoic (e.g. Kvaček et al. 2002; Zetter et al. 2002), but the grains have mostly been lumped into various fossil species (along with *Tilia* type pollen) under the form-genus *Intratriporopollenites* Pflug et Thomson (*cf*. Stuchlik et al. 2014).

##### Ecological implications

*Craigia* is a small genus comprising only two modern species, *C. yunnanensis* and *C. kwangsiensis* H.H. Hsue. Nothing is known about the living plant of *C. kwangsiensis* and it might even be extinct. *Craigia yunnanensis* are small deciduous trees, up to 20 m tall, occurring mostly in open forests, at an elevation of 500 to 1600 m, in southern China and adjacent northernmost Vietnam (Ya et al. 2007b). Today’s *Craigia* prefer warm temperate climates with dry winters and hot summers (*Cwa*; semihumid-meridional vegetation element; File S1), with mean annual temperatures (MATs) ranging from 13 to 21 °C, annual precipitation of 1074 to 1688 mm, and coldest mean month temperature of 6.3 to 14.2 °C (Fang et al. 2009). According to Kvaček et al. (2005), *C*. *yunnanensis* is usually confined to mesic notophyllous evergreen broadleaved forests, with some populations occurring at higher elevations in mixed conifer and evergreen broadleaved forests or mixed mesophytic forests. Since macrofossils of *Craigia* (e.g. *C*. *bronnii, Dombeyopsis lobata*) are often associated with plants of moist habitats, Kvaček (2004) suggested that during the Miocene the Central European *Craigia* were part of wetland forest vegetation thriving in backswamp forests and along streams and in deltas. Based on the Lavanttal plant assemblage such a scenario is possible; taking into account its present habitat, however, it cannot be ruled out that *Craigia* was only/also part of more well-drained forests at higher elevation surrounding the Lavanttal Basin.

*Genus* Reevesia *Lindl.*

Reevesia sp.

(*Figures 3G–L, 4A–F*)

##### Description

Pollen, monad, oblate, circular to convex quadrangular in polar view, elliptic in equatorial view; polar axis 11–14 µm long in LM, equatorial diameter 19–23 µm wide in LM, 16–22 µm wide in SEM; brevitetracolporate; exine 0.8–1.2 µm thick, nexine thinner than sexine, nexine thickened around endopori, sexine slightly protruding in area of endopori; sculpture reticulate in LM, heterobrochate reticulate in SEM, duplicolumellate, columellae numerous and closely spaced, muri as high as columellae, muri rounded, lumina polygonal, lumina in distal polar area with numerous freestanding columellae, nexine in proximal polar area perforate (SEM).

##### Remarks

The pollen morphology of extant *Reevesia*, including *R. lofouensis* Chun et Hsue, *R. longipetiolata* Merr. et Chun, *R. formosana* Sprague, *R. pupescens* Mast. and *R. thyrsoidea* Lindl., is known from LM studies by Krutzsch (1970b), Huang (1972), Petrov and Drazheva-Stamatova (1972), Long et al. (1985) and Wang et al. (1995).

##### Fossil record

*Reevesia* pollen is morphologically unique and easy to identify. For this reason, even though it occurs only in low numbers in palynological samples, it has a well-established fossil record. The fossil pollen record of *Reevesia* and *Reevesiapollis* Krutzsch has been summarised in detail by Krutzsch (1970b), Petrov and Drazheva-Stamatova (1972), Raine et al. (2011), and Stuchlik et al. (2014). These accounts suggest that *Reevesia* had a wide continental European distribution, with records extending from the middle Paleocene until the Pliocene, but with a peak occurrence during the Miocene. Pollen of this genus is also known from the Eocene to Miocene of Russia and from the Miocene to Pleistocene of New Zealand.

##### Ecological implications

The genus *Reevesia* comprises about 20 species of evergreen and deciduous trees (8–18 m tall) with a South Asian distribution (excluding the two Central American species alternatively placed in *Veeresia*). Fifteen of the species occur in China, where 12 of them are endemic (Ya et al. 2007a). Most of the Chinese *Reevesia* occur in warm temperate climate with dry winter and hot summer (*Cwa*), a third of the taxa occur (also) in equatorial savannah climate with dry winter (*Aw*-climate), and one third (also) in fully humid warm temperate climate with hot summer (*Cfa*), in combination, the genus can be characterised as a tropical-meridional element (File S1). The majority of the taxa endure a MAT in the range of 14 to 25 °C, and an annual precipitation somewhere between 1268 and 2992 mm (Fang et al. 2009). All the *Reevesia* species in China have very restricted distributions except for *R. pubescens* (*Aw, Cwa, Cfa*); it grows under a MAT of 2 to 25 °C, and annual precipitation of 741 to 2435 mm (Fang et al. 2009). *Reevesia pubescens* Mast. is the only species extending into regions with winter frost, enduring coldest mean month temperatures down to −7 °C (Fang et al. 2009). According to Ya et al. (2007a) *Reevesia* occurs in both open valley forests as well as dense montane forests; it can also be found on forested slopes/hillsides and along riverbanks. Based on habitats of the potential modern analogues of the Lavanttal fossils, especially *R*. *pubescens*, the Miocene *Reevesia* was likely part of forest vegetation surrounding the basin, occurring in well-drained valley- and hillside forests, above the main wetlands.

*Genus* Tilia *L.*

Tilia *sp. 1*

(Figure 4G–L)

##### Description

Pollen, monad, oblate, convex triangular in polar view, elliptic in equatorial view; polar axis 23–28 µm long in LM, equatorial diameter 41–48 µm wide in LM, 35–45 µm in SEM; brevitricolporate, planaperturate; exine 1.7–2.2 µm thick, nexine slightly thinner than sexine, nexine remarkably thickened around endopori; sculpture reticulate in LM, reticulate to microreticulate in SEM, heterobrochate reticulate in distal polar area, heterobrochate microreticulate in proximal polar area, lumina elliptic to circular, muri rounded, single branched columellae filling lumina, space between branches appearing as perforations in the lumina (SEM).

##### Remarks

Pollen of recent *Tilia* has been studied in detail by various authors using both LM and SEM (e.g. Chambers & Godwin 1971; Zhang & Chen 1984; Huo et al. 1985; Christensen & Blackmore 1988; Perveen et al. 2004) and even TEM (e.g. Halbritter & Hesse 2016; Sam 2016).

##### Fossil record

The earliest macrofossils assigned to *Tilia* are bracts (‘type B’; Manchester 1994b) from the late Eocene of North America. In Europe, *Tilia* type B bracts are known from the Oligocene (Manchester 1994b), suggesting that the genus spread to Eurasia via the North Atlantic land bridge. *Tilia* bracts are also known from the Oligocene of Asia (‘type C’; Manchester 1994b). For a more complete summary on the macrofossil record of *Tilia* consult the monograph on the genus by Pigott (2012). *Tilia* and *Tilia*-like pollen from the Paleocene to Pliocene of Europe have been assigned to the form-genus *Intratriporopollenites* Pflug et Thomson using various species names (e.g. Mai 1961; Muller 1981; Stuchlik et al. 2014). Combined LM and SEM analyses have shown that the early Cainozoic records (Paleocene to late Oligocene) of *Intratriporopollenites* in central Europe do not represent *Tilia* but other extant genera (*Mortoniodendron*: Grímsson et al. 2017b) and unknown or extinct groups (Gastaldo et al. 1998). True *Tilia* pollen is not known from western Eurasian sediments prior to the Oligocene, but is frequent in Miocene sediments and has been documented, among others, from Iceland (Denk et al. 2011), Germany (Ferguson et al. 1998), Poland (Stuchlik et al. 2014), Austria (Kovar-Eder et al. 1998; Zetter 1998) and Turkey (Bouchal et al. 2016b, 2017; Bouchal 2019).

##### Ecological implications

The monograph on *Tilia* by Pigott (2012) accepts only 23 extant species with 14 subspecies. *Tilia* are deciduous trees (up to 45 m tall) occurring mostly throughout the temperate zone of the Northern Hemisphere. Two species occur in North and Central America, four species are growing in Europe and western Asia, and 17 species are distributed in eastern Asia (e.g. Jones 1968; Ya et al. 2007b; Pigott 2012; Hanes 2015). *Tilia americana* L. covers a wide geographical region in eastern North America, occurring in both fully humid warm temperate climates and snow climates, with either hot or warm summers (*Cfa, Cfb, Dfa, Dfb*; nemoral vegetation element; File S1). In China, most *Tilia* species occur in warm temperate climates with hot summers that are either fully humid (*Cfa*) or winter-dry (*Cwa*). Only some East Asian *Tilia* species (≤ 6 spp.) extend into cooler areas with warm summers (*Cwb*) or (≤ 4 spp.) extend into snow climates (*Dwa, Dwb*). None of the *Tilia* species in China are particularly prominent in lowland wetland vegetation, but are predominantly reported from altitudes above 600 m and even occurring in mountain forests up to *c*. 4000 m (Ya et al. 2007b). Only very few of the *Tilia* species (≤ 3 spp.) in China extend into areas where the MAT drops below 0 °C, most of the taxa (≥ 11 spp.) occur in areas where the MAT is above 5 °C, and the annual precipitation ranges between 250 and 1997 mm (Fang et al. 2009). According to Ya et al. (2007b) *Tilia* trees in China are components of evergreen or mixed evergreen and deciduous forests, occurring on well-drained steep valley slopes and mountainsides. A similar habitat is suggested for the middle Miocene *Tilia* from the Lavanttal Basin.

Tilia *sp. 2*

*(Figure 5A–F)*

**Figure 5.**
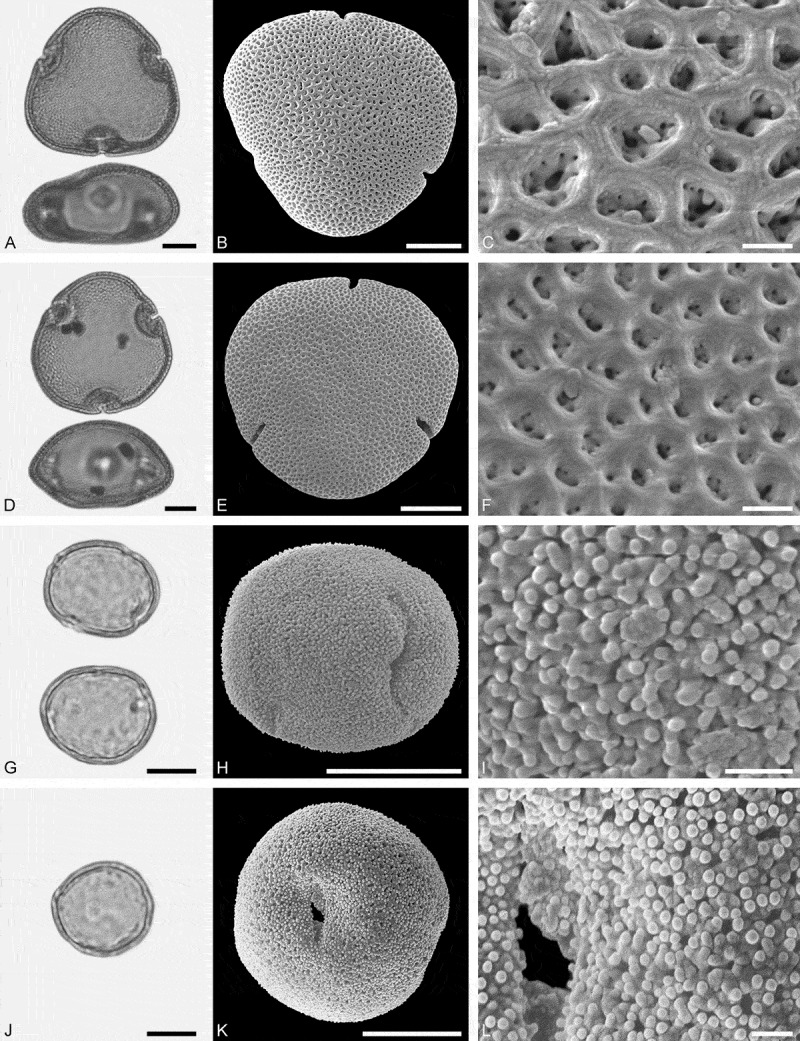
Light microscopy (LM) (**A, D, G, J**) and scanning electron microscopy (SEM) (**B, C, E, F, H, I, K, L**) micrographs of dispersed fossil Malvaceae and Anacardiaceae pollen. **A–C.** *Tilia* sp. 2, close-up of central part of distal polar area. **D–F.** *Tilia* sp. 2, close-up of central part of proximal polar area. **G–I.** *Pistacia* sp., close-up showing sculpture in area of mesocolpium. **J–L.** *Pistacia* sp., close-up of sculpture around aperture. Scale bars 10 µm (A, B, D, E, G, H, J, K), 1 µm (C, F, I, L).

##### Description

Pollen, monad, oblate, convex triangular in polar view, elliptic in equatorial view; polar axis 24–27 µm long in LM, equatorial diameter 44–48 µm wide in LM, 39–43 µm wide in SEM; brevitricolporate, planaperturate; exine 1.4–2.2 µm thick, nexine slightly thinner than sexine, nexine remarkably thickened around endopori; sculpture reticulate in LM, reticulate to microreticulate in SEM, heterobrochate reticulate in distal polar area, heterobrochate microreticulate in proximal polar area, lumina elliptic to circular, muri broadly rounded, muri with a striate suprasculpture, single branched columellae filling lumina, space between branches appearing as perforations in the lumina (SEM).

##### Remarks

This pollen type differs from *Tilia* sp. 1 in having striate suprasculpture. Identical striate suprasculpture observed in SEM has been documented by Perveen et al. (2004, figure 4G) for extant *Tilia platyphyllos* Scop. as well as fossil Miocene *Tilia* pollen (Bouchal 2019, SI3, figure 12A–C).

Order Sapindales Juss. ex Bercht. et J.Presl

Family Anacardiaceae R.Br.

*Genus* Pistacia *L.*

Pistacia *sp.*

(Figure 5G–L)

##### Description

Pollen, monad, spheroidal, outline ± circular in polar and equatorial view; polar axis 20–22 µm long in LM, 17–22 µm long in SEM, equatorial diameter 21–24 µm wide in LM, 18–22 µm wide in SEM; (brevi)tetracolpate, colpi short; exine 0.9–1.1 µm thick, nexine thinner than sexine; tectate to semitectate; sculpture psilate to scabrate in LM, perforate to microreticulate in SEM, area between perforations nanobaculate (nanoechinate) or muri with nanobaculate (nanoechinate) suprasculpture (SEM).

##### Remarks

*Pistacia* is characterised by unique pollen that has been described in detail using LM (e.g. Erdtman 1952; Beug 2004; Bahramabadi et al. 2017) and also SEM (Díez 1986; Belhadj et al. 2007; Perveen & Qaiser 2010; Halbritter & Weis 2016; Li et al. 2011). The apertures described from this genus have either been classified as pori, meridionally elongated pori, or as short colpi. The apertures of the fossil pollen from Lavanttal correspond to the colpi of *P*. *terebinthus* L. as figured by Halbritter and Weis (2016).

##### Fossil record

The European macrofossil record of *Pistacia* is meagre. According to Mai (1995b), leaf and fruit records are rare and confined to the middle Oligocene and Pliocene of Italy, and the Miocene of Germany, Hungary and Austria. *Pistacia* pollen is also very rare. So far, the only European records are from the Miocene of France and Spain (cf. Muller 1981).

##### Ecological implications

The recently revised classification of *Pistacia* by Al-Saghir and Porter (2012) recognised nine species (and five subspecies). *Pistacia* are deciduous or evergreen shrubs to small trees (up to 20 m tall). The genus has a disjunct northern hemispheric distribution, with a single species in south-western United States and Central America (*P. mexicana* Humb.), five species in Mediterranean Europe and Africa (*P. atlantica* Desf., *P. chinensis* Bunge, *P. terebinthus* L., *P. vera* L., *P. lentiscus* L.), six species in West to Central Asia (*P. atlantica, P. chinensis, P. eurycarpa* Yalt., *P. khinjuk* Stocks, *P. vera, P. lentiscus*), and two species in East Asia (*P. chinensis, P. weinmannifolia* J. Poiss. ex Franch.) (Al-Saghir & Porter 2012). The current diversification centre of *Pistacia*, the Mediterranean region (summer-dry warm temperate climates), is believed to have been established following the latest Miocene (Xie et al. 2014). Hence, the Mediterranean (and alike) *Pistacia* species cannot be used as informative modern-day analogues for the Lavanttal fossils. This leaves the East Asian species as potential modern analogues of the fossil taxon, *P*. *chinensis* and *P. weinmannifolia*. The former shows a disjunct distribution across Asia into Africa. In China, *P*. *chinensis* has a wide distribution, thriving in winter-dry equatorial savannah climates (*Aw*) to fully humid warm temperate climate with hot summers (*Cfa*), and enduring coldest mean month temperatures down to −13 °C (Fang et al. 2009). It occurs alongside *P*. *weinmannifolia* in warm temperate climates with hot or warm summer (*Cwa, Cwb*;), hence, the genus can be classified as a tropical-meridional vegetation element (File S1). Both taxa are found in hill and mountain forests, growing mostly on hard substrates (rocky soils and limestone). *Pistacia chinensis* occurs at an elevation between 100 and 3600 m, and *P. weinmanniifolia* at elevation between 500 and 2700 m (Tianlu & Barfod 2008). The Miocene *Pistacia* from Lavanttal was most likely growing outside the lowland wetland area, on well-drained hills and mountain slopes, especially on hard or rocky substrate.

Family Rutaceae Juss.

*Genus* Zanthoxylum *L.*

Zanthoxylum *sp. 1*

*(Figure 6A–G)*

**Figure 6.**
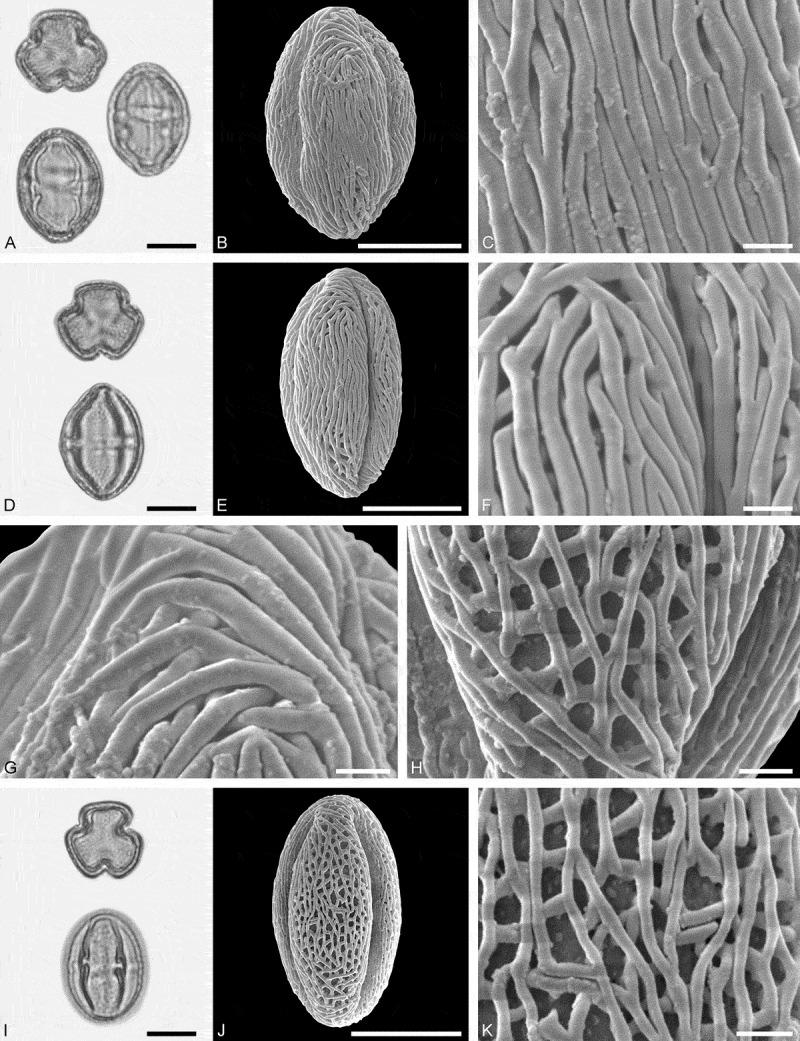
Light microscopy (LM) (**A, D**, **I**) and scanning electron microscopy (SEM) (**B, C, E, F, G, H, J, K**) micrographs of dispersed fossil Rutaceae pollen. **A–C, G.** *Zanthoxylum* sp. 1. **C.** Close-up of striae in area of central mesocolpium. **G.** Close-up of striae in polar area. **D–F.** *Zanthoxylon* sp. 1, close-up of striae along colpi. **H–K.** *Zanthoxylon* sp. 2. **J.** Close-up of striae in polar area. **K.** Close-up of striae in area of mesocolpium. Scale bars 10 µm (A, B, D, E, I, J), 1 µm (C, F, G, H, K).

##### Description

Pollen, monad, prolate, outline lobate in polar view, elliptic in equatorial view; polar axis 23–25 µm long in LM, 23–25 µm long in SEM, equatorial diameter 16–18 µm wide in LM, 12–14 µm wide in SEM; tricolporate, colpi long and bow-like, endopori lalongate elliptic to rectangular, margins of endopori perpendicular to polar axis are thickened; exine 0.8–1.0 µm thick (LM), nexine thinner than sexine, sexine slightly thickened in polar areas; tectate; sculpture scabrate to striate in LM, striate in SEM, striae 0.6–0.7 µm wide, striae closely spaced in mesocolpium, striae wider apart in polar areas, partly striato-reticulate in polar areas (SEM).

##### Remarks

Pollen of extant *Zanthoxylum* has been studied in LM by Huang (1972), Barth (1980), Wang et al. (1995), and Mayer (1996), in SEM by Barth (1980), Mayer (1996), Li et al. (2011), and Cao et al. (2014), and in TEM by Barth (1980). The pollen morphology of *Zanthoxylum* is unique within Rutaceae (Mayer 1996). The suite of characters that permit identification of this genus include long and bow-like colpi and lalongate rectangular endopori observed with LM. Also, margins of endopori perpendicular to polar axis are thickened (LM), and the sculpture is striato-reticluate to striate. The only genus producing similar pollen is *Toddalia*, but their pollen is smaller, spheroidal (versus prolate in *Zanthoxylum*), and the colpi are shorter.

##### Fossil record

According to Gregor (1989) and Mai (1995b) *Zanthoxylum* has a reliable fossil seed record from the middle Eocene to Pliocene in Europe, the Oligocene to Pleistocene of Asia, and the Miocene of North America. The leaf record is scarce and doubtful (Mai 1995b). This pollen type has rarely been reported (e.g. Gastaldo et al. 1998; Ferguson et al. 1998) even though it is often occurring in Oligocene to Pliocene sediments of Central Europe (R. Zetter, pers. obs.).

##### Ecological implications

*Zanthoxylum* comprises about 225 species with a pantropical distribution but extending into temperate latitudes in eastern Asia and eastern North America. The plants are either deciduous or evergreen and occur as woody climbers, shrubs, or small trees that can be up to 20 m tall (Dianxiang et al. 2008; Kubitzki et al. 2011). Chinese *Zanthoxylum* plants occur in various habitats including lowland forests, hillside thickets, moist (river) valley forests, and open upland forests, and are often conspicuous at forest margins. The plants are found mostly in lowlands, at an elevation from sea-level up to 800 m, but also occur in various forest types reaching an elevation of 2000 to 3000 m (Dianxiang et al. 2008). The majority of the *Zanthoxylum* species in China occur in fully humid and/or winter-dry warm temperate climates with hot and/or warm summers (*Cfa, Cwa, Cwb*; meridio-nemoral vegetation element; File S1). Only a few species extend into areas with equatorial climates (*Aw*; tropical-meridional vegetation element; File S1). Only three species in China seem to extend into a winter-dry snow climate with hot summers (*Dwa*). Based on the modern life form and pollen morphology of the genus it is impossible to conclude if the Miocene *Zanthoxylum* from Lavanttal was deciduous or evergreen and if it was a climber, shrub or a small tree. Also, the habitat ranges of modern species suggest that *Zanthoxylum* could have been a component of lowland wetland forests and/or various hillside forests and/or mountain forests.

Zanthoxylum sp. 2

(Figure 6H–K)

##### Description

Pollen, monad, prolate, outline trilobate in polar view, elliptic in equatorial view; polar axis 21–23 µm long in LM, 20–22 µm long in SEM, equatorial diameter 15–17 µm wide in LM, 14–16 µm wide in SEM; tricolporate, colpi long and bow-like, endopori lalongate rectangular, margins of endopori perpendicular to polar axis are thickened; exine 0.8–0.9 µm thick (LM), nexine thinner than sexine; semitectate; sculpture reticulate in LM, striato-reticulate to striato-microreticulate in SEM, striae 0.2–0.3 µm wide; margo striate, perforate (SEM).

##### Remarks

Even though the two *Zanthoxylum* pollen types are very similar in size and outline they unambiguously belong to two different species because *Zanthoxylum* sp. 2 has much narrower striae (0.2–0.3 versus 0.6–0.7 µm wide) and is also striato-reticulate over most of the pollen surface versus striate in *Zanthoxylum* sp. 1.

Family Sapindaceae Juss.

*Genus* Acer *L.*

Acer *sp. 1*

*(Figure 7A–I)*

**Figure 7.**
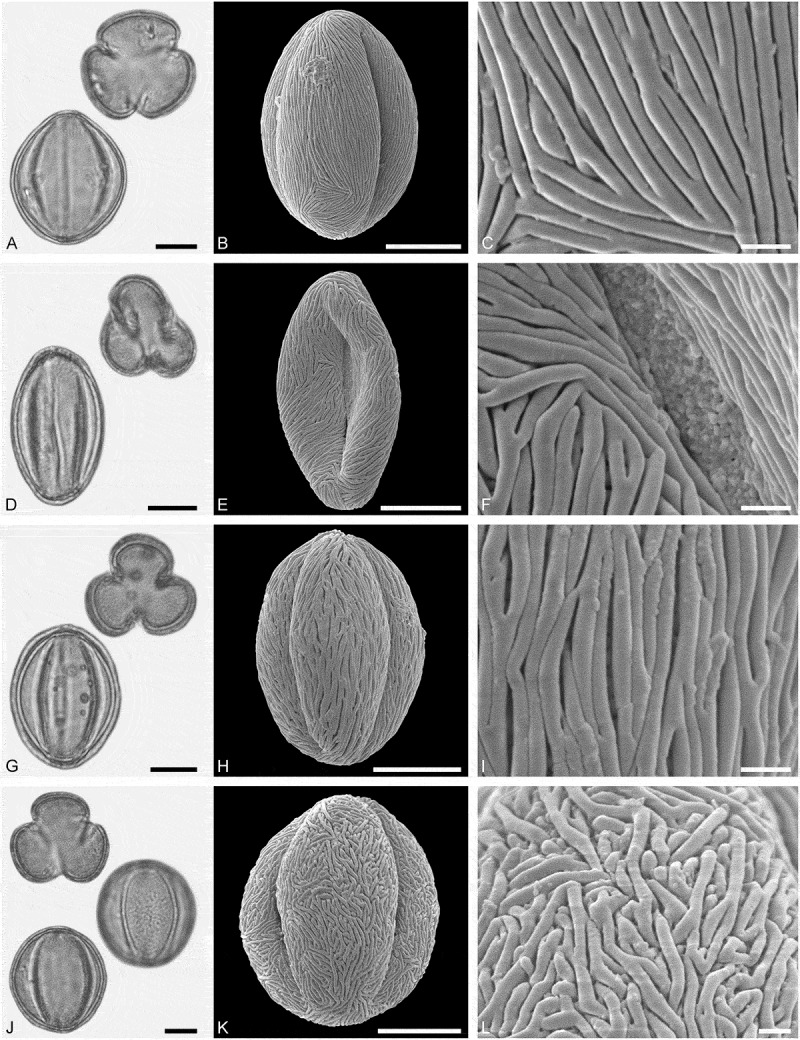
Light microscopy (LM) (**A, D, G, J**) and scanning electron microscopy (SEM) (**B, C, E, F, H, I, K, L**) micrographs of dispersed fossil Sapindaceae pollen. **A–C.** *Acer* sp. 1, close-up of striae in are of mesocolpium. **D–F.** *Acer* sp. 1, close-up of colpus membrane. **G–I.** *Acer* sp. 1, close-up striae in area of mesocolpium. **J–L.** *Acer* sp. 2, close-up of striae in area of mesocolpium. Scale bars 10 µm (A, B, D, E, G, H, J, K), 1 µm (C, F, I, L).

##### Description

Pollen, monad, prolate, outline lobate in polar view, elliptic in equatorial view; polar axis 29–33 µm long in LM, 27–31 µm long in SEM, equatorial diameter 18–27 µm wide in LM, 15–22 µm wide in SEM; tricolpate, colpi long; exine 1.2–1.6 µm thick, nexine slightly thinner or as thick as sexine; tectate; sculpture striate in LM and SEM, striae long and straight, running mostly parallel to the polar axis, closely spaced, often dividing and/or fusing, 0.3–0.5 µm wide (SEM).

##### Remarks

Pollen morphology of both American and Eurasian *Acer* species has been studied in detail using LM and SEM by Biesboer (1975), Adams and Morton (1976), Clarke and Jones (1978), Philbrick and Bogle (1981), Pozhidaev (1993), Fürstl (2002), Li et al. (2011), Miyoshi et al. (2011) and Siahkolaee et al. (2017). Most authors agree that *Acer* pollen can be distinguished into four groups based on the sculpture observed in LM and/or SEM; a striate group (including most American and Eurasian taxa of various sections including sect. *Acer* and *Platanoidea*, the dominant groups in western Eurasia), a rugulate (or rugulose, cf. Biesboer 1975) group (including unrelated *A*. *negundo* L. and *Acer* sect. *Rubra*), a striato-reticulate (or microreticulate, cf. Biesboer 1975) group (including *A. saccharum* Marshall, the North American representative of sect. *Acer*), and a rugulate-echinate (or granulate, cf. Biesboer 1975) ‘group’ (including *A*. *carpinifolium* Siebold et Zucc., a systematically and genetically isolated species; Grimm et al. 2006; Renner et al. 2008).

##### Fossil record

The massive macrofossil record (leaves and samaras) of *Acer* has been summarised by numerous authors including Walther (1972), Tanai (1983), Wolfe and Tanai (1987), Oterdoom (1994), Mai (1995b), Boulter et al. (1996), Manchester (1999), McClain (2000), and Grimm et al. (2007). Based on these accounts, the current consensus is that the earliest accepted *Acer* fossils are from the Paleocene of North America. *Acer* is then believed to have dispersed across Beringia into Asia during the Eocene and finally reaching Europe during the Oligocene. *Acer* was one of the most species-rich and widely distributed woody genera in the Miocene of Europe (e.g. Walther 1972; Mai 1995b; Boulter et al. 1996). In western Eurasia, striate *Acer* pollen grains, similar to the fossils, have been documented among others from the Oligocene and Miocene of Germany (Schmid 2000; Kottik 2002), and the Miocene of Iceland (Denk et al. 2011), Poland (Stuchlik et al. 2014), and Turkey (Bouchal et al. 2016b, 2017; Bouchal 2019; Denk et al. 2019).

##### Ecological implications

*Acer* is one of the largest tree genera of the Northern Hemisphere comprising *c*. 126 species (e.g. de Jong 1994; Acevedo-Rodríguez et al. 2011) divided into 16 sections and 19 series (cf. de Jong 1994). Most of the species occur in Eurasia (*c*. 116) and especially China (including many microspecies); relatively few species are wide-spread including many of the western Eurasian species (sections *Acer, Platanoidea*) and most of the nine species found in North America (van Gelderen 1994); in East Asia, the most widespread species are *A. pictum* Thunb. (section *Platanioidea*) and *A. palmatum* Thunb. (section *Palmata*), the latter cultivated worldwide as a garden ornamental. The striate fossil *Acer* sp. 1 and *Acer* sp. 2 pollen types correspond to pollen from numerous modern North American and Eurasian taxa and it is, therefore, difficult to affiliate them to any of the modern, genetically supported sections. These pollen grains could have originated from lowland wetland trees or from individuals that were part of well-drained highland or even mountain forests surrounding the basin.

Acer *sp. 2*

(*Figure 7J–L*)

##### Description

Pollen, monad, prolate, outline lobate in polar view, elliptic in equatorial view; polar axis 32–34 µm long in LM, 28–30 µm long in SEM, equatorial diameter 29–31 µm wide in LM, 24–26 µm wide in SEM; tricolpate, colpi long; exine 1.3–1.5 µm thick, nexine thinner than sexine; tectate; sculpture striate in LM and SEM, striae short and sinuous, often dividing, 0.4–0.6 µm wide, sometimes short parts of striae protruding, striae separated by wide grooves (SEM).

##### Remarks

*Acer* sp. 2 differs from the previous taxon mainly in the length and orientation of the striae. The striae in *Acer* sp. 2 are shorter, more sinuous, often dividing and they are generally wider than those in *Acer* sp. 1.

Acer *sp. 3*

(*Figure 8A–I*)

**Figure 8.**
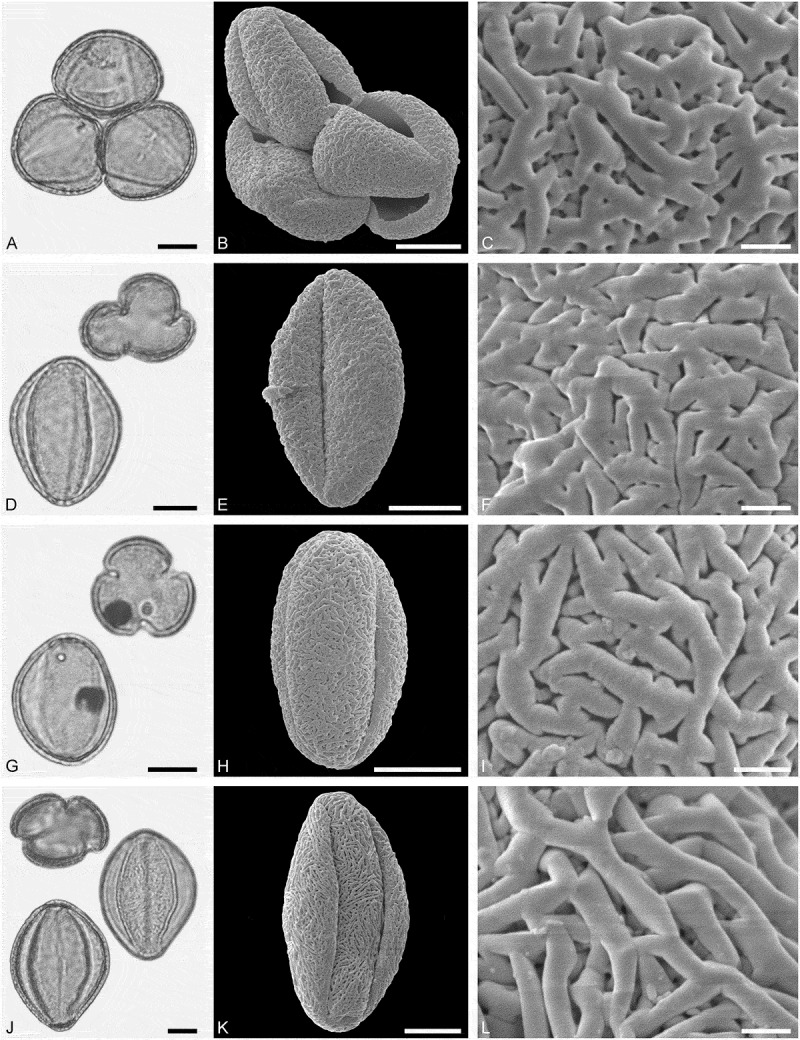
Light microscopy (LM) (**A, D, G, J**) and scanning electron microscopy (SEM) (**B, C, E, F, H, I, K, L**) micrographs of dispersed fossil Sapindaceae pollen. **A–C.** *Acer* sp. 3, tetrad, close-up of striae in area of mesocolpium. **D–F.** *Acer* sp. 3, close-up of striae in area of mesocolpium. **G–I.** *Acer* sp. 3, close-up striae in area of mesocolpium. **J–L.** *Acer* sp. 4, close-up of striae in area of mesocolpium. Scale bars 10 µm (A, B, D, E, G, H, J, K), 1 µm (C, F, I, L).

##### Description

Pollen, monad (rarely tetrads), prolate, outline lobate in polar view, elliptic in equatorial view; polar axis 27–34 µm long in LM, 28–34 µm long in SEM, equatorial diameter 21–25 µm wide in LM, 16–19 µm wide in SEM; tricolpate, colpi long; exine 1.1–1.4 µm thick, nexine thinner than sexine; tectate; sculpture scabrate to striate in LM, rugulate-striate in SEM, striae short and sinuous, 0.5–0.8 µm wide, striae branching radially or in series, striae separated by irregularly outlined grooves and perforations (SEM).

##### Remarks

*Acer* sp. 3 clearly differs from the two previously described *Acer* sp. 1 and *Acer* sp. 2 by its rugulate-striate sculpture. This pollen type falls within the rugulate (rugulose) group of Biesboer (1975). Modern taxa known to produce similar pollen are *A*. *negundo* (sect. *Negundo*) but also *A. saccharinum* and *A. rubrum* L. of the distantly related sect. *Rubra* (Adams & Morton 1976; Philbrick & Bogle 1981); according to Renner et al. (2008), both lineages originated during the first radiation phase that took place at least ~55–35 ma ago). On a side note, the fossil species *A*. *tricuspidatum* Bronn (leaves) is associated with *Acer* sect. *Rubra* based on its cuticular analysis (Walther 1972). Leaves of this species have not been reported from St. Stefan but have been identified and collected from a Badenian Lavanttal locality (Schaßbach, J. M. Bouchal, pers. obs.).

##### Fossil record

In western Eurasia, rugulate *Acer* pollen grains, similar to the *Acer* sp. 3 fossils, have been documented from the Miocene of Iceland (Denk et al. 2011), Germany (Kmenta 2011), and Turkey (Bouchal et al. 2017; Bouchal 2019).

##### Ecological implications

*Acer negundo* are deciduous trees or shrubs. The species has a vast range throughout North America (Canada, United States, Mexico, and Guatemala), occurring mostly in forested valleys, along riverbanks, and in mixed riparian forests (van Gelderen 1994). Concerning its vast distribution *A. negundo* has a wide climate range, occurring under various warm temperate and snow climates (*Cfa, Cfb, Dfa, Dfb, Cwa, Dwa, Csb*; predominantly a nemoral vegetation element; File S1). *Acer saccharinum* are deciduous trees (up to 40 m tall) native to eastern and central North America (eastern United States, Canada) (van Gelderen 1994). They occur mostly in lowland wetlands and are found in river floodplains, along streams and lakes, and even in swamps (Gabriel 1990b). They grow under fully humid warm temperate to snow climates with hot or warm summers (*Cfa, Cfb, Dfa, Dfb*; nemoral vegetation element; File S1). It is possible that the plants producing the *Acer* sp. 3 pollen grains were European counterparts of the North American *A. negundo* or *A. rubrum-saccharinum* lineages and part of the lowland wetland vegetation during the Miocene of Lavanttal, occurring in mixed forest along lakes and streams, and floodplains and backswamps.

Acer *sp. 4 (aff*. A. saccharum)

(*Figure 8J–L*)

##### Description

Pollen, monad, prolate, outline lobate in polar view, elliptic in equatorial view; polar axis 43–45 µm long in LM, 42–44 µm long in SEM, equatorial diameter 32–34 µm wide in LM, 25–28 µm wide in SEM; tricolpate, colpi long; exine 1.4–1.6 µm thick, nexine thinner than sexine; tectate; sculpture striate in LM and SEM, striae 0.5–0.7 µm wide, often branching and interwoven, striae separated by wide grooves, striae fused along margin of colpi (SEM).

##### Remarks

This pollen type is much larger than the *Acer* sp. 1–3. Its sculpture type is different to the long striae of *Acer* sp. 1 and the radially branching striae observed in *Acer* sp. 3. This pollen type falls within the striato-reticulate (or microreticulate) group of Biesboer (1975). Modern taxon known to produce similar pollen is *A. saccharum*, a widespread, morphologically variable species that now includes all North American members of sect. *Acer* (Adams & Morton 1976; van Gelderen 1994).

##### Ecological implications

*Acer saccharum* are deciduous trees, up to 40 m tall, occurring in eastern Canada and central and eastern United States to Mexico and Guatemala (van Gelderen 1994). *Acer saccharum* is not a wetland taxon and does not occur in swamps but prefers well-drained slopes at intermediate elevation. It is found in rich forests, on slopes, in ravines and valleys, and near streams. In the Mixed Mesophytic Climax Forest of the eastern United States, *A. saccharum* appears as a dominant member of the forest canopy in association with *Aesculus, Fagus, Liriodendron, Quercus, Tilia* and *Tsuga* (Godman et al. 1990; Gabriel 1990a). *Acer saccharum* is growing under fully humid warm temperate to snow climates with hot or warm summers (*Cfa, Cfb, Dfa, Dfb*; nemoral vegetation element; File S1). It is possible that the trees producing this type of *Acer* pollen, during the Miocene of Lavanttal, were found at moderate elevation above the main wetland as part of ravine vegetation and well-drained species-rich hillside and slope forests.

Order Santalales R.Br. ex Bercht. et J.Presl

Family Santalaceae R.Br.

*Genus* Arceuthobium *M.Bieb.*

Arceuthobium sp.

(*Figure 9A–F*)

**Figure 9.**
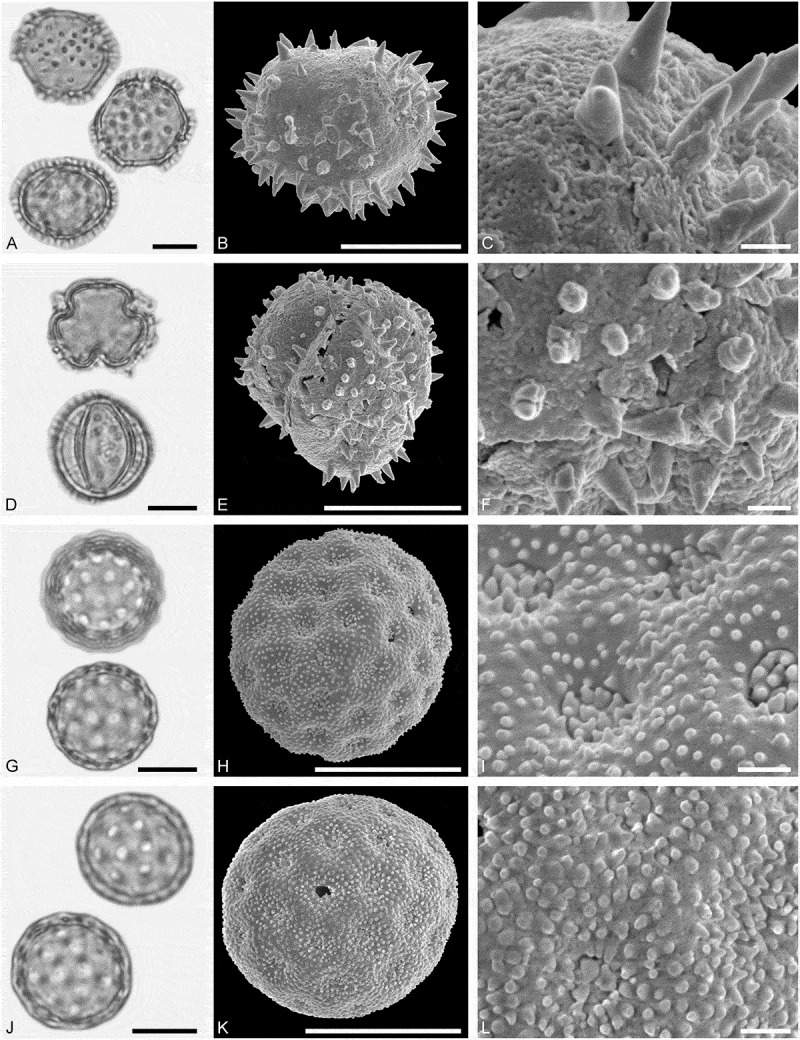
Light microscopy (LM) (**A, D, G, J**) and scanning electron microscopy (SEM) (**B, C, E, F, H, I, K, L**) micrographs of dispersed fossil Santalaceae and Amaranthaceae pollen. **A–C.** *Arceuthobium* sp., close-up of granulate and echinate sculpture. **D–F.** *Arceuthobium* sp., close-up of echinate sculpture in area of mesocolpium. **G–I.** Amaranthaceae gen. et spec. indet. 1, close-up showing opercula and microechinate sculpture of the surrounding tectum. **J–L.** Amaranthaceae gen. et spec. indet. 1, close-up showing opercula and microechinate sculpture of the surrounding tectum. Scale bars 10 µm (A, B, D, E, G, H, J, K), 1 µm (C, F, I, L).

##### Description

Pollen, monad, spheroidal to oblate, outline hexagonal to lobate in polar view, circular to elliptic in equatorial view; polar axis 20–22 µm long in LM, 17–19 µm long in SEM, equatorial diameter 20–24 µm wide in LM, 16–20 µm wide in SEM; tricolpate with three alternating pseudocolpi (heteroaperturate), colpi much longer than pseudocolpi; exine (excl. echini) 1.0–1.1 µm thick (LM); tectate; sculpture echinate in LM, microrugulate to granulate and echinate in SEM, echini 1.1–1.8 µm long, widely spaced, conical in form, loosely attached to tectum, colpus membrane granulate (SEM).

##### Remarks

Pollen morphology of the genus was investigated using LM and SEM by Hawksworth and Wiens (1972), also listing all previous studies on the pollen morphology of *Arceuthobium*. Fossil pollen affiliated to modern *Arceuthobium* has been assigned to the pollen form-genus *Spinulaepollis* Krutzsch (e.g. Krutzsch 1962; Stuchlik et al. 2014).

##### Fossil record

The pre-Quaternary fossil record of *Arceuthobium* is scarce and mostly based on dispersed pollen. The earliest pollen records are from the middle Eocene of Europe, but pollen of this genus is known from the middle Eocene to Pliocene of Germany (Krutzsch 1962, 1970a; Sontag 1966; Krutzsch & Lenk 1973; Menke 1975; 1976; Thiele-Pfeiffer 1980; Gastaldo et al. 1998), the late Eocene of UK (unpublished data, F. Grímsson), the late Oligocene to Miocene of Poland and Austria (Stuchlik 1964; Ziembińska-Tworzydło 1974; Oszast & Stuchlik 1977; Hochuli 1978; Ziembińska-Tworzydło & Ważyńska 1981; Rębas 1985; Stuchlik et al. 1990; 2014; Kohlman-Adamska 1993; Ziembińska-Tworzydło et al. 1994b; Piwocki & Ziembińska-Tworzydło 1997; Meller et al. 1999; Słodowska & Paruch-Kulczycka 2008; Worobiec 2009; Worobiec & Szulc 2010; Stuchlik et al. 2014), and the Miocene of Hungary (Nagy 1969, 1985), Turkey (Bouchal 2019) and north-eastern China (unpublished data, F. Grímsson). The mostly LM-based European pollen record documents two different morphotypes, the smaller *Spinulaepollis arcethobioides* Krutzsch and the larger *Spinulaepollis major* (Stuchlik) Stuchlik, suggesting the coexistence of at least two different *Arceuthobium* lineages during the Eocene to Miocene of Europe. Previous studies on the pollen morphology of *Arceuthobium* (e.g. Hawksworth & Wiens 1972) suggest *Arceuthobium* being stenopalynous in LM (term *sensu* Halbritter et al. 2018), and the pollen from different extant species are hard to distinguish except in some few taxa. If this was the case in fossil species each morphotype could easily represent more than a single biological taxon. There are not many reports on *Arceuthobium* macrofossils worldwide. The few Palaeogene records are fragments of shoots and fruiting inflorescences originating from the late Eocene Baltic amber (Sadowski et al. 2017b). The only Neogene macrofossil records are shoots, flowers and fruits, from the late Miocene of Poland (Łańcucka-Środoniowa 1980). Interestingly, the Baltic amber seems to encompass six different co-occurring *Arceuthobium* species (see Sadowski et al. 2017b). Such a diversity hot spot is only known from current-day Mexico, established following the Miocene (Hawksworth & Wiens 1996). The high number of fossil species in the Baltic amber raises the question whether sexual dimorphism, that is apparent in extant taxa (Hawksworth & Wiens 1996), played a role and lead to an overestimation of the actual palaeo-diversity. Extant *Arceuthobium* are very host selective and exclusive, raising the additional question whether this was also the case during the Eocene and whether the Baltic amber forests had sufficient host numbers to satisfy at least six different parasites. Sadowski et al. (2017a) note a considerable variety of conifers (at least 12 different taxa) thriving in the Baltic amber forests, including *Calocedrus, Quasisequoia, Taxodium* (Cupressaceae), *Cupressospermum* (Geinitziaceae), *Abies, Cathaya, Nothotsuga, Pseudolarix, Pinus* (Pinaceae), and *Sciadopitys* (Sciadopityaceae). Finding an appropriate host was apparently not a problem and the high number of different conifers could easily have sustained a diverse *Arceuthobium* flora. The same can be said about the Miocene Lavanttal flora: Grímsson and Zetter (2011) reported at least 20 different conifers, including various Cupressaceae (5 spp.), Pinaceae (14 spp.) and *Sciadopitys*.

Comparing primitive versus advanced characteristics of extant *Arceuthobium*, Hawksworth and Wiens (1972) hypothesised that this genus originated in north-eastern Asia during the early Cainozoic. *Arceuthobium* apparently then dispersed westwards to the Mediterranean area, south into Africa, and north-eastwards across the Bering land bridge into the Americas. Following first molecular evidence, Hawksworth and Wiens (1996) adjusted their theory by adding a possible migration event across the North Atlantic land bridge during the early Cainozoic and constraining the migration across the Bering land bridge as a second event during the Miocene. This would imply that American *Arceuthobium* originated from two different stocks, an Eocene western Eurasian stock and a Miocene East Asian stock. This contradicts the current molecular phylogenetic framework of the genus by Nickrent et al. (2004) supporting a monophyletic origin of all American taxa with *A. azoricum* resolved as sister to the American clade, i.e. a single colonisation event via the North Atlantic. The current fossil record suggests a middle Eocene European origin for *Arceuthobium* (see earlier). Likely, *Arceuthobium* dispersed southwards into Africa already during the Eocene, similar migration routes have recently been proposed for other plant groups, e.g. the Loranthaceae (from Asia; Grímsson et al. 2017a, 2018b) and the Picrodendraceae (from Europe; Grímsson et al. 2019). The restricted present-day distribution of *Arceuthobium* in Africa is most likely the result of post-Miocene climate and vegetation changes. How, if and when *Arceuthobium* migrated across the North Atlantic land bridge is uncertain. Palynological data place the genus at the gate of the corridor during the late Eocene (UK record, unpublished data, F. Grímsson), but studies on Palaeogene floras of Greenland hold no such records (Grímsson et al. 2014, 2015b; 2016b, 2016c, 2018a). Also, the Miocene floras of Iceland, both macrofloras and microfloras, have been studied in detail and yield no records of *Arceuthobium*, but *Viscum* is present (e.g. Grímsson et al. 2005, 2007a, 2007b; Denk et al. 2005, 2010, 2011; Grímsson & Símonarson 2008). The earliest American fossil records of *Arceuthobium* are from the Miocene (summarised by Hawksworth & Wiens 1996). The fossil pollen from China noted herein represent the only known record of this genus from East Asia. If the hypothesis holds that *Arceuthobium* originated in Europe during the Eocene, it would be unlikely that it migrated into Asia before the closure of the Turgai Seaway that took place during the early Oligocene (Akhmetiev & Reshetov 1996). It is more likely that *Arceuthobium* only reached Asia following its closure in the Oligocene and/or Miocene, at the same time as numerous other plant groups dispersed between these continental areas (e.g. Akhmetiev & Reshetov 1996; Manchester 1999; Manchester et al. 2009a; Denk et al. 2012; Grímsson et al. 2012a).

##### Ecological implications

*Arceuthobium* is a small genus with a mostly northern hemispheric distribution. Hawksworth and Wiens (1996) recognised 42 spp., but on the background of molecular phylogenetic data Nickrent et al. (2004) reduced their number to 26. Of these, a single species occurs in Europe, *A. oxycedri* (DC) Bieb., extending from Spain eastwards through Mediterranean Europe and Africa (Morocco, Algeria) to the Middle East and the Himalayas of India and China (Hawksworth & Wiens 1996; Polhill & Wiens 1998). This species along with *A. juniperi-procerae* Chiovenda and *A. tibetense* H.S Kiu et W. Ren are considered the most basal/primitive within the genus (Nickrent et al. 2004). *Arceuthobium juniperi-procerae* is confined to the highlands of Eritrea, Ethiopia and central Kenya (Polhill & Wiens 1998). *Arceuthobium tibetense* is only known from the Mainling area in eastern Xizang, Tibet (Hawksworth & Wiens 1996). These three species are placed in Subgenus *Arceuthobium* Section *Arceuthobium* and were placed as sister lineages to the clade composing the remaining Asian taxa, *A. chinense* Lecomte, *A. minutissimus* JD Hooker, *A. pini* Hawksws. et Wiens and *A. sichuanense* (H.S. Kiu) Hawksw. et Wiens of Section *Chinense* (Nickrent et al 2004). *Arceuthobium minutissimum* is known from the Himalayas in Pakistan, India, Nepal, and Bhutan, but the other three Asian taxa (*A. chinense, A, pini* and *A. sichuanense*) are restricted to south-western China (Hawksworth & Wiens 1996). In addition to these Old World taxa, *A*. *azoricum* Hawksw. et Wiens, an endemic to the Azores, occurs on the islands of Terceira, San Jorge, Pico, and Faial in the North Atlantic Ocean (Hawksworth & Wiens 1996). All remaining *Arceuthobium* (18 spp.) are found in the Americas, occurring in Canada (3 spp.), United States (8 spp.), Mexico (12 spp., greatest diversity), Hispaniola (1 sp.) and continental Central America (3 spp.) (Hawksworth & Wiens 1996; Nickrent et al. 2004).

*Arceuthobium* are stem hemiparasites that exclusively parasitise conifers and have been found on *Abies, Cupressus, Juniperus, Keteleeria, Larix, Picea, Pinus, Pseudotsuga* and *Tsuga* (Hawksworth & Wiens 1972, 1996; Polhill & Wiens 1998; Kuijt 2015). Individual taxa can be very selective and parasitising only a single host species, some are found on several different conifers but usually do not cross genera. Dual parasitism (two *Arceuthobium* species infecting a single host tree) is extremely rare and they rarely parasitise trees that are already occupied by other parasitic plants. It is also believed that climate is a limiting factor in the distribution of *Arceuthobium*, especially when they do not occur throughout the whole range of their host (Hawksworth & Wiens 1996). At present, *Arceuthobium* is mostly absent from all equatorial and polar climates (*A*- and *E*-climates) and thrives well in the drier variants (summer or winter-dry) of the warm temperate climates (*Cs, Cw*), arid climates (*B*-climates) and snow climates (*D*-climates). Being a parasite, *Arceuthobium* is depended on its host. Based on the rich conifer flora previously documented from Lavanttal by Grímsson and Zetter (2011), *Arceuthobium* could have been infecting trees both in the lowland wetlands as well as in the surrounding hills and mountains.

Order Caryophyllales Juss. ex Bercht. et J.Presl

Family Amaranthaceae Juss.

Amaranthaceae gen. et spec. indet. 1

(*Figure 9G–L*)

##### Description

Pollen, monad, spheroidal, circular in outline; diameter 17–19 µm wide in LM, 15–17 µm wide in SEM; pantoporate, 48–51 pori, pori circular with opercula, diameter of pori 1.3–1.5 µm wide (SEM); exine 0.9–1.2 µm thick (LM); tectate; sculpture scabrate in LM, nanoechinate, perforate in SEM, nanoechini closely packed, opercula nanoechinate (SEM).

##### Remarks

Pollen of Amaranthaceae is invariable (relatively thick exine, pantoporate, nanoechinate, perforate) and has been extensively studied using both LM and SEM (e.g. Nowicke 1975; Skvarla & Nowicke 1976; Müller & Borsch 2005; Olvera et al. 2006; Dehghani & Akhani 2009; Hamdi et al. 2009; Toderich et al. 2010; Borsch et al. 2018). Except for the subfamily Celosioideae (e.g. Borsch 1998), characterised by reticulate pollen, dispersed pollen of this family cannot be assigned to taxa below the family level owing to the substantial overlap in pollen morphology.

##### Fossil record

The macrofossil record of this family is sparse (e.g. Friis et al. 2011). Seeds of *Chenopodium* have been reported from the early Miocene of the Randecker Maar, southern Germany (Gregor 1982). The few additional Eurasian Neogene macrofossil records representing the family are summarised in Mai (1995b). The earliest pollen records are from the Maastrichtian of Canada (Srivastava 1969) and the United States (Nichols 2002). From the Palaeogene, pollen of this family is more often reported and is known from the Paleocene to Eocene of the United States (Zetter et al. 2011; Bouchal et al. 2016a), the Eocene of Central and East Asia (Wang & Zhao 1980; Hoorn et al. 2012), and the Eocene of western Eurasia (Akkiraz et al. 2006; Worobiec & Gedl 2018). From the Neogene onward Amaranthaceae pollen is very common in Eurasia (summarised by Muller 1981; Stuchlik et al. 2009). Fossil Amaranthaceae pollen is commonly assigned to the form-genus *Chenopodipollis* Krutzsch (e.g. Stuchlik et al. 2009).

##### Ecological implications

The Amaranthaceae (including Chenopodiaceae) comprise about 170 genera and over 2400 species. The family has a cosmopolitan distribution, but Amaranthaceae *s. str*. (excluding the Chenopodiaceae) are mainly tropical and the former Chenopodiaceae are subtropical to temperate. The plants are mostly annual or perennial herbs, subherbs or shrubs, rarely lianas or trees (e.g. Townsend 1993; Kühn 1993). Most Amaranthaceae *s. str*. occur in regions with low humidity and rainfall, some taxa are aquatic, others occur in inundated or damp depressions, and some are found near water in montane forests (Townsend 1993). The Chenopodiaceae can be dominant components in both marshes and (semi-)deserts. Chenopodiaceae prefer coastal or xeric habitats, they are light demanding plants, and many of them have a dominant position within certain vegetation units. *Chenopodium* and *Atriplex*, with non-pronounced xeromorphic attributes, are typical members of vegetation occurring on sandy or stony soil at riverbanks or rocky habitats (Kühn 1993). Based on the cosmopolitan distribution of the family and the overlapping pollen morphology between taxa, potential modern analogues for the fossil Amaranthaceae gen. et spec. indet. 1 and 2 cannot be established. Nonetheless, the fossil pollen grains likely originated from herbaceous plants growing in open and sunny areas, maybe on sandy/stony banks of streams running into the lowland wetlands, or on rocky outcrops in the surrounding highlands.

Amaranthaceae gen. et spec. indet. 2

(*Figure 10A–C*)

**Figure 10.**
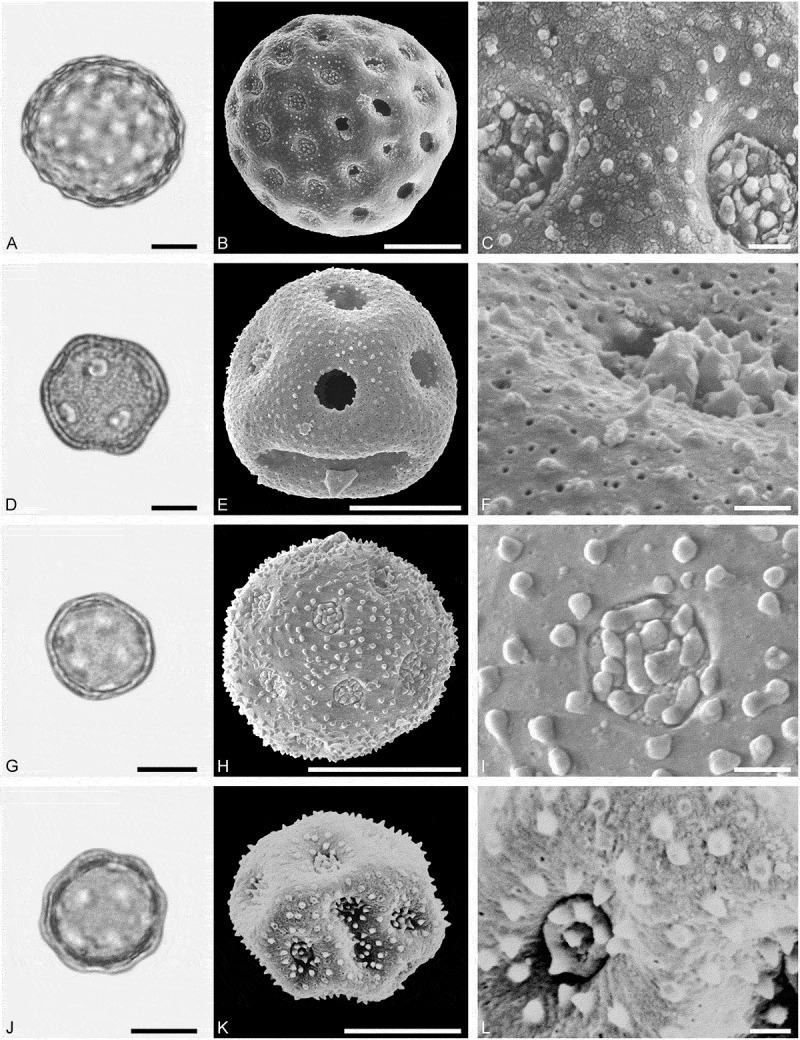
Light microscopy (LM) (A, D, G, J) and scanning electron microscopy (SEM) (**B, C, E, F, H, I, K, L**) micrographs of dispersed fossil Amaranthaceae and Caryophyllaceae pollen. **A–C.** Amaranthaceae gen. et spec. indet. 2, close-up showing opercula and microechinate sculpture of the surrounding tectum. **D–F.** Caryophyllaceae gen. et spec. indet. 1, close-up showing opercula and microechinate and perforate sculpture of the surrounding tectum. **G–I.** Caryophyllaceae gen. et spec. indet. 2, close-up showing opercula and microechinate sculpture of the surrounding tectum. **J–L.** Caryophyllaceae gen. et spec. indet. 3, close-up showing opercula and microechinate sculpture of the surrounding tectum. Scale bars 10 µm (A, B, D, E, G, H, J, K), 1 µm (C, F, I, L).

##### Description

Pollen, monad, spheroidal, circular in outline; diameter 33–35 µm wide in LM, 31–33 µm wide in SEM; pantoporate, 57–63 pori, pori circular with opercula, diameter of pori 2.4–2.6 µm wide (SEM); exine 1.2–1.5 µm thick (LM); tectate; sculpture scabrate in LM, nanoechinate, perforate in SEM, nanoechini widely spaced, opercula microechinate (SEM).

##### Remarks

The Amaranthaceae gen. et spec. indet. 2 is much larger than the indet.1 type pollen, the pori are also much wider (1.3–1.5 vs 2.4–2.6), and the density of nanoechini is much higher in the previous taxon than the latter.

Family Caryophyllaceae Juss.

Caryophyllaceae gen. et spec. indet. 1

(*Figure 10D–F*)

##### Description

Pollen, monad, spheroidal, circular in outline; diameter 26–28 µm wide in LM, 22–24 µm wide in SEM; pantoporate, 9–12 pori, pori circular with opercula; exine 1.5–1.6 µm thick (LM); tectate; sculpture scabrate in LM, microechinate, perforate in SEM, perforations at regular interval, radially arranged around pori, perforations more frequent around pori, opercula microechinate, microechini of opercula clustered and fused (SEM).

##### Remarks

The morphology (LM, SEM) and ultrastructure (TEM) of Caryophyllaceae pollen has been studied extensively, e.g. Chanda (1962), Vishnu-Mittre and Gupta (1964), McNeill and Bassett (1974), Iwarsson (1977), McNeill and Crompton (1978), Ghazanfar (1984), Straka and Friedrich (1988), Al-Eisawi (1989), Punt and Hoen (1995), Kaplan (2008), Ataşlar et al. (2009), Al-Taie and Almousawi (2018), Doğan and Erdem (2018), and Ullah et al. (2018, 2019). Caryophyllaceae pollen are commonly pantoporate (all Caryophylloideae); in the other two subfamilies, Alsinoideae and Paronychioideae, one can also find, colpate or colporate pollen. The general morphology and SEM sculpture of spherical pantoporate Caryophyllaceae pollen is very alike and the different genera are hard to distinguish based on dispersed pollen (within and across subfamilies). This makes affiliation of the fossil Caryophyllaceae pollen from Lavanttal to any extant infrafamily taxon impossible. Concerning pore number and pore membrane, Caryophyllaceae gen. et spec. indet. 1 displays morphological similarities to the ‘*Areanaria serpyllifolia* type’ of Punt and Hoen (1995).

##### Fossil record

The macrofossil record of Caryophyllaceae is sparse. A fossil inflorescence, *Caryophylloflora paleogenica* G.J.Jord. et Macphail, with *in situ* pollen, is known from the Eocene of Tasmania, Australia (Jordan & Macphail 2003). Fossil seeds, assigned to *Hantsia*, are described from the Eocene of England, UK (Chandler 1960; 1961, 1963, 1964). All other macrofossils are post-Miocene seed records, assigned to *Arenaria, Cerastium, Cucubalus, Minuartia, Silene*, and *Stellaria* (e.g. Zazula et al. 2005; Thompson et al. 2011; Huang et al. 2013). The palynological record of Caryophyllaceae is summarised in Muller (1981), Jordan and Macphail (2003), and Stuchlik et al. (2009). Even though Caryophyllaceae-type pollen (*Periporopollenites* Pflug et Thompson) seems to occur in Upper Cretaceous to Palaeogene sediments of Australia (cf. Jordan & Macphail 2003), their earliest European records (mostly assigned to *Caryophyllidites* Couper and *Vaclavipollis* Krutzsch) are of Miocene age (cf. Muller 1981; Stuchlik et al. 2009).

##### Ecological implications

The Caryophyllaceae is a large family that includes about 86 genera and *c*. 2200 species of annual or perennial herbs, or subshrubs, rarely shrubs or small trees. Three genera compose large shrubs or small trees such as *Sanctambrosia* (San Ambrosia Island, Chile), *Alsinidendron* (Hawaii) and *Schiedea* (Hawaii). The family is cosmopolitan, including Antarctica, with a primarily Holarctic distribution, and a centre of diversity (54 of the 86 genera occur) in the Mediterranean and Irano-Turanean region. Caryophyllaceae are found in various habitats, especially in dry open areas and mountainous regions (up to 7000 m above sea level [a.s.l.]). They also occur in moist temperate forests or meadows, moist areas in tropical Afromontane regions, but are absent from tropical lowland rain forests (Bittrich 1993). Based on the cosmopolitan distribution of Caryophyllaceae, and the fact that the fossil Lavanttal pollen cannot be assigned to a particular genus (due to overlapping in pollen morphology), further speculations regarding climate preferences of potential modern analogues are impractical. It is likely that the fossil pollen originates from herbaceous plants that were part of open habitats, maybe meadows or shrublands at forest margins, or formed part of the understory in the rich highland forests surrounding the lowland wetlands.

Caryophyllaceae gen. et spec. indet. 2

(*Figure 10G–I*)

##### Description

Pollen, monad, spheroidal, circular in outline; diameter 17–19 µm wide in LM, 14–16 µm wide in SEM; pantoporate, 15–18 pori, pori circular with opercula; exine 0.8–1.0 µm thick (LM); tectate; sculpture scabrate in LM, microechinate, perforate in SEM, perforations irregularly distributed, opercula microechinate, microrugulate (SEM).

##### Remarks

This pollen type differs from the previously described not only in size of the pollen, but also in the size and shape of echini and the number and conspicuousness of perforations. Also, the echini on the opercula of the Caryophyllaceae gen. et spec. indet. 2 are often fused to elongated, a feature not observed in pollen of the indet. 1 type. This pollen type is morphologically similar (pore membrane configuration) to the ‘*Silene vulgaris* type’ of Punt and Hoen (1995).

Caryophyllaceae gen. et spec. indet. 3

(*Figure 10J–L*)

##### Description

Pollen, monad, spheroidal, circular in outline; diameter 19–21 µm wide in LM, 18–20 µm wide in SEM; pantoporate, 15–18 pori, pori circular with opercula; exine 1.6–1.8 µm thick (LM); tectate; sculpture scabrate in LM, microechinate, perforate in SEM, opercula microechinate, microechini on opercula specially arranged, with a single centrally placed microechinus surrounded by a circle of six or more echini (SEM).

##### Remarks

*–* This pollen type differs from Caryophyllaceae gen. et spec. indet. 1 in both size and number of apertures. It is considerably smaller and equipped with more apertures. The pollen wall in Caryophyllaceae gen. et spec. indet. 3 is much thicker than that of Caryophyllaceae gen. et spec. indet. 2. The indet. 3 pollen type shows morphological similarities (pore membrane configuration) to the ‘*Dianthus superbus* type’ of Punt and Hoen (1995).

Family Polygonaceae Juss.

Subfamily Polygonoideae Eaton

Tribe Persicarieae Dumort

*Genus* Persicaria *(L.) Mill.*

Persicaria *sp.*

(*Figure 11A–F*)

**Figure 11.**
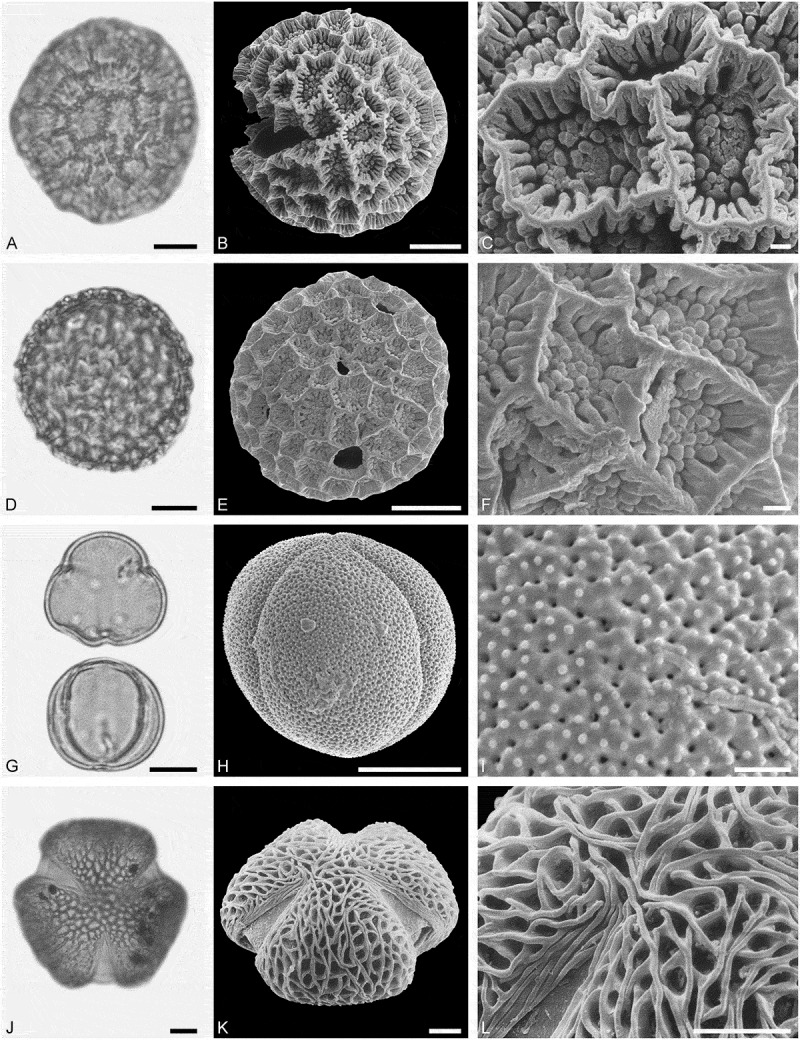
Light microscopy (LM) (**A, D, G, J**) and scanning electron microscopy (SEM) (**B, C, E, F, H, I, K, L**) micrographs of dispersed fossil Polygonaceae and Cornaceae pollen. **A–C.** *Persicaria* sp., close-up showing heterobrochate reticulate sculpture. **D–F.** *Persicaria* sp., close-up showing crested muri and short freestanding columellae. **G–I.** *Rumex*, close-up of microechinate and perforate sculpture in area of mesocolpium. **J–L.** *Alangium* sp., close-up of polar area. Scale bars 10 µm (A, B, D, E, G, H, J, K), 1 µm (C, F, I, L).

##### Description

Pollen, monad, spheroidal, outline circular; diameter 40–46 µm wide in LM, 33–45 µm wide in SEM; pantoporate; exine 2.4–2.8 µm thick (LM), nexine thinner then sexine; semitectate; sculpture reticulate in LM, heterobrochate reticulate in SEM, duplicolumellate, columellae high and closely spaced, muri crested, lumen with short densely spaced freestanding columellae (SEM).

##### Remarks

Pollen morphology (LM, SEM) and ultrastructure (TEM) of *Persicaria*, generic circumscription following Haraldson (1978), Ronse Decraene and Akeroyd (1988), Sanchez et al. (2011), and Schuster et al. (2015), has been studied by various authors, but referred to both *Persicaria* and/or *Polygonum* (e.g. Wodehouse 1931; Hedberg 1946; Nowicke & Skvarla 1977; van Leeuwen et al. 1988; Hong & Hedberg 1990; Hong 1994; Zhou et al. 1999; Ayodele 2005; Yasmin et al. 2010b). Based on pollen morphology observed in some of the 131 *Persicaria* species accepted by POWO (2017), two main pollen types are occurring in this genus, a pantocolpate/pantoporate type and a 3-colpate type. The fossil pantoporate pollen from Lavanttal falls within the framework of the pantoporate ‘*Persicaria*’/‘*Polygonum persicaria*’ pollen types of Wodehouse (1931), Hedberg (1946), van Leeuwen et al. (1988) and Yasmin (2010b, ‘*Persicaria* type’). Pantoporate pollen has been documented for *P*. *acuminata* (Kunth) M.Gómez, *P. amphibia* (L.) Delarbre, *P. arifolia* (L.) Haraldson, *P. barbata* (L.) H.Hara, *P. bungeana* (Turcz.) Nakani, *P. cespitosa* (Blume) Nakai, *P. careyi* (Olney) Greene, *P. criopolitana* (Hance) Migo, *P. dichotoma* (Blume) Masam., *P. foliosa* (H.Lindb.) Kitag, *P. glabra* (Willd.) M.Gómez, *P. hydropiper* (L.) Delarbre, *P. hydropiperoides* (Michx.) Small, *P. jucunda* (Meisn.) Migo, *P. lanigera* (R.Br.) Soják, *P. lapathifolia* (L.) Delarbre, *P. longiseta* (Bruyn) Kitag., *P. maculosa* Gray, *P. minor* (Huds.) Opiz, *P. meisneriana* (Cham. et Schltdl.) M.Gómez, *P. mitis* (Schrank) Assenov, *P. orientalis* (L.) Spach, *P. pensylvanica* (L.) M. Gómez, *P. perfoliata* (L.) H.Gross, *P. sagittata* (L.) H.Gross, *P. senticosa* (Meisn.) H.Gross, *P. sinuata* (Royle ex Bab.) H.Gross, *P. stagnina* (Buch.-Ham. ex Meisn.) M.A. Hassan, *P. tinctoria* (Aiton) Spach, and *P. thunbergii* (Siebold et Zucc.) H.Gross.

##### Fossil record

The macrofossil record of the genus was recently summarised/revised by Doweld (2017): Fossil *Persicaria* leaves are known from the Miocene of Romania, *P. asymmetrica* (Givulescu et Ţicleanu) Doweld, and Poland, *P. lapathiformis* (Kownas) Iljinskaja and *P. zablockii* Doweld. Also, Doweld reports fossil fruits from the Miocene of Ukraine and Siberia, Russia, *P. vladichenica* (Negru) Doweld and *P. omoloica* Doweld, and the Pliocene of Germany and western Russia, *P. wolfii* (Kinkelin) Doweld and *P. dorofeevii* Arbuzova. The LM-based palynological record of *Persicaria*, recorded as the form-genus *Persicarioipollis* Krutzsch, is summarised in Muller (1981), Song et al. (2004), Stuchlik et al. (2009), Schuster et al. (2013), and Doweld (2017). According to these summaries, *Persicaria*-type pollen has its earliest appearance in the Paleocene of western/central Europe (France, Germany), the Eocene of China, and the Oligocene of Russia. It became very common during the Neogene and had a worldwide distribution after the Miocene.

##### Ecological implications

*Persicaria* comprises about 150 species of annual or perennial herbs. It is a cosmopolitan genus, occurring mostly in the Northern Hemisphere with some taxa extending into subtropical and tropical regions, from sea-level to high elevation (Brandbyge 1993; Heywood et al. 2007). *Persicaria* plants usually occur near or in the water and are found along the coastline, at riversides, along streams and river banks, along the shoreline of lakes, in swamps, fens, ponds, bogs, tidal marshes, wet ravines, marshlands, moist prairies, on floodplains, and other types of wetlands. The plants can be part of various different forest types (including deciduous forests, mixed deciduous and evergreen forests, and evergreen forests), but are typically concentrated in openings and/or at the forest margins (e.g. Li et al. 2003; Freeman & Reveal 2005; Kantachot et al. 2010; Li 2014; Liang & Li 2014). For a detailed account on the ecology and habitat of *P*. *amphibia* consult Partridge (2001). The cosmopolitan distribution of *Persicaria* and the similarities in pollen morphology of its species, render fossil *Persicaria* useless for climate inferences using potential modern analogues. The fossil *Persicaria* pollen from Lavanttal probably originates from herbaceous plants, growing in or close to moving or stagnant water as part of lowland wetlands or in open areas of the forests surrounding the basin, at forest margins and/or along streams.

Tribe Rumiceae Dumort

*Genus* Rumex *L.*

Rumex *sp.*

(*Figure 11G–I*)

##### Description

Pollen, monad, spheroidal, outline circular to trilobate in polar view, circular in equatorial view; polar axis 24–25 µm long in LM, 22–23 µm long in SEM, equatorial diameter 24–25 µm wide in LM, 23–24 µm wide in SEM; tricolporate, colpi long and narrow, endopori lolongate, not wider than colpi; exine 0.9–1.0 µm thick (LM), nexine thinner than sexine, nexine slightly thickened around endopori; tectate; sculpture scabrate in LM, microechinate, perforate in SEM, perforations in pits (SEM).

##### Remarks

Pollen of *Rumex* has been studied using LM (e.g. Wodehouse 1931; den Nijs et al. 1980; van Leeuwen et al. 1988; Cheng & Feng 1996; Ayodele 2005; Yasmin et al. 2010a), SEM (e.g. Nowicke & Skvarla 1977; den Nijs et al. 1980; van Leeuwen et al. 1988; Cheng & Feng 1996; Yasmin et al. 2010a; Soleimani et al. 2014) and TEM (e.g. Nowicke & Skvarla 1977; Cheng & Feng 1996). The sculpture of *Rumex* pollen observed in SEM seems to be very similar between taxa, although there is a considerable variation in aperture configuration both within and between species. *Rumex* pollen grains are predominantly tricolporate, but grains with 4–9 apertures also occur (van Leeuwen et al. 1988, table II; Cheng & Feng 1996, table II). The fossil *Rumex* pollen from Lavanttal is of the typical tricolporate type and resembles pollen from many of the extant species.

**Table I.** Angiosperm pollen described in this study in comparison to Klaus (1984).

This study	Klaus (1984)
*Ludwigia* sp. 1	x
*Ludwigia* sp. 2	*Ludwigia*
*Craigia* sp.	x
*Reevesia* sp.	*Reevesia*
*Tilia* sp. 1	?*Tilia caroliniana*
*Tilia* sp. 2	?*Tilia caroliniana*
*Pistacia* sp.	x
*Zanthoxylum* sp. 1	x
*Zanthoxylum* sp. 2	*Toddalia*
*Acer* sp. 1	*Acer franchetti*
*Acer* sp. 2	x
*Acer* sp. 3	*Acer saccharinum*
*Acer* sp. 4	x
*Archeuthobium* sp.	*Archeuthobium*
Amaranthaceae gen. et spec. indet. 1	?Chenopodiaceae Form A
Amaranthaceae gen. et spec. indet. 2	Chenopodiaceae Form B
Caryophyllaceae gen. et spec. indet. 1	x
Caryophyllaceae gen. et spec. indet. 2	x
Caryophyllaceae gen. et spec. indet. 3	x
*Persicaria* sp.	x
*Rumex* sp.	x
*Alangium* sp.	x
*Cornus* sp.	x
*Nyssa* sp.	*Nyssa*
*Diospyros* sp.	x
*Andromeda* sp.	x
*Arbutus* sp.	*Arbutus*
*Empetrum* sp.	x
*Erica* sp. 1	x
*Erica* sp. 2	x
*Erica* sp. 3	x
*Erica* sp. 4	x
*Erica* sp. 5	?*Ericaceoipoll*. cf. *acastus*
Ericaceae gen. et spec. indet. 1	?*Ericaceoipoll*. cf. *ericius*
Ericaceae gen. et spec. indet. 2	x
Sapotaceae gen. et spec. indet. 1	x
Sapotaceae gen. et spec. indet. 2	x
*Pouteria* sp. 1	x
*Pouteria* sp. 2	x
*Sideroxylon* sp.	x
*Rehderodendron* sp.	*Rhoipites pseudocingulum*
*Symplocos* sp. 1	x
*Symplocos* sp. 2	x
*Symplocos* sp. 3	x
*Symplocos* sp. 4	*Symplocos*
*Symplocos* sp. 5	x

Note: Question marks indicate when we are uncertain if the light microscopy (LM)-based description and/or micrographs by Klaus correspond to our pollen types, the x indicates that this pollen type was not reported by Klaus.

**Table II.** Preferred general forest types of extant species of genera/families represented among the Lavanttal Myrtales, Sapindales, Santalales, Caryophyllales, Cornales and Ericales.

	RF	MF	DF
*Ludwigia*	x		
*Craigia*	x		x
*Reevesia*		x	x
*Tilia*		x	x
*Pistacia*		x	x
*Zanthoxylum*	x	x	x
*Acer*	x	x	x
*Archeuthobium*	x	x	x
*Amaranthaceae*	x	x	x
*Caryophyllaceae*		x	x
*Persicaria*	x	x	x
*Rumex*	x	x	x
*Alangium*		x	x
*Cornus*	x	x	x
*Nyssa*	x	x	
*Diospyros*	x	x	x
*Andromeda*	x		
*Arbutus*	x	x	x
*Empetrum*			x
*Erica*	x	x	x
*Ericaceae*	x	x	x
*Madhuca*	x	x	
*Manilkara*	x	x	
*Mimusops*	x	x	
*Palaquim*	x	x	
*Xantolis*	x	x	
*Pouteria*	x	x	
*Sideroxylon*	x	x	
*Rehderodendron*		x	x
*Symplocos*	x	x	x

Note: RF, riparian forest; MF, mesic forest; DF, dry forest.

##### Fossil record

There are currently no convincing pre-Miocene macrofossil records of *Rumex*. The fossil record is composed solely of fruits from the Neogene and Quaternary of Eurasia (e.g. Reid 1920; Szafer 1954; Nikitin 1957; Łańcucka-Środoniowa 1979; Friis 1985; Mai 1995b). The palynological record is also scarce and confined to Miocene and younger sediments (e.g. Muller 1981).

##### Ecological implications

*Rumex* comprises about 195 species of perennial or annual herbs (POWO 2017). Like the other found Polygonaceae, it is a cosmopolitan genus but with a preference of the temperate regions of the world (Brandbyge 1993). *Rumex* occurs from sea-level to an elevation of about 4300 m. The plants are found in various environments (e.g. coastal, alluvial and montane habitats), including, swamps, marshes, bogs, shores of lakes, banks of streams and rivers, grasslands, (wet) meadows, (moist) valleys, (dry) mountain slopes, forest margins, sandy and gravelly shores, sandy planes, sand dunes, rocky outcrops, rocky fissures, and even saline deserts and sands (Li et al. 2003; Freeman & Reveal 2005). As in the case of other Polygonaceae, it is useless for climate reconstruction. Based on the variable habitats of extant *Rumex* it is uncertain if the pollen originates from plants that were growing in the lowland wetlands or suitable patches in the highland and/or mountain forests surrounding the basin.

Clade Asterids

Order Cornales Link.

Family Cornaceae Bercht. et J.Presl

*Genus* Alangium *Lam.*

*Alangium sp.*(aff. *A. kurzii*)

(*Figure 11J–L*)

##### Description

Pollen, monad, oblate to spheroidal, outline elliptic in equatorial view, trilobate in polar view; polar axis 56–58 µm long in LM, equatorial diameter 69–72 µm wide in LM, 73–76 µm wide in SEM; tricolporate, colpi broad, endopori elliptic with two short opposite equatorially placed crevasses (LM); exine 4.5–4.6 µm thick, nexine thinner than sexine (LM); semitectate; sculpture reticulate in LM, heterobrochate reticulate in SEM, becoming striatoreticulate towards apertures, margo striate, colpus membrane irregularly granulate, muri narrow and high, partly interwoven (SEM).

##### Remarks

The pollen morphology of *Alangium* has been studied quite thoroughly by Reitsma (1970), but see also Chao (1954), Straka et al. (1967), Eyde et al. (1969), Eyde (1972), Cerceau-Larrival et al. (1984) and Martin et al. (1996). These palynological studies were based on the *c*. 20 taxa known at that time. Recent revision on three of the four currently accepted sections within *Alangium* (sections *Alangium, Conostigma, Rhytidandra*) by de Wilde and Duyfjes (2016, 2017a, 2017b) shows that they comprise 43 species. Section *Marlea* has not been revised so far but is believed to hold less than 10 taxa (de Wilde & Duyfjes 2016, 2017a, 2017b). The pollen morphology of *Alangium* is therefore in need of revision to clarify the characteristics of each section and to assess whether important and/or diagnostic features are overlapping between sections. The fossil *Alangium* pollen from Lavanttal is heterobrochate-reticulate to striatoreticulate and with narrow muri. Until now, *Alangium kurzii* Craib (section *Marlea*) is the only extant taxon known to produce comparable pollen (Reitsma 1970, plates 7–10).

##### Fossil record

The fossil record of *Alangium* goes back to the Eocene of the Americas, Europe, India, southeast Asia and Australia and has been summarised by Eyde et al. (1969), Morley (1982), Phadtare and Thakur (1990), Martin et al. (1996) and Shatilova and Kokolashvili (2014). Fossil pollen grains attributed to extant *Alangium kurzii* (sect. *Marlea*) are known from the middle Oligocene to late Miocene of Germany (Krutzsch 1962), the Miocene of Hungary (Nagy 1962, 1969, 1973), Poland (Słodkowska 1998; Ziembińska-Tworzydło et al. 1994b; Stuchlik et al. 2014), Austria (Kovar-Eder et al. 1998) and Bulgaria (Ivanov 1997), the Miocene to Pliocene of Georgia (Stuchlik & Shatilova 1987; Shatilova & Kokolashvili 2014), and the Pliocene of southeast Asia (Morley 1982). Fossil *Alangium* pollen grains of this type are mostly assigned to *Alangipollis barghoornianum* (Traverse) Krutzsch that was originally described from the Miocene of Vermont, USA, by Traverse (1955); later emended by Krutzsch (1962) adding fossils from the middle Oligocene of Germany.

##### Ecological implications

*Alangium kurzii* is a deciduous shrub or tree that can be up to 28 m tall. It occurs from 50–1600 m a.s.l. and has a vast distribution across southeast Asia (including Myanmar, Thailand, Vietnam, Malaysia, Indonesia and the Philippines) and East Asia (including southern and eastern China, Korea and Japan) (Bloembergen 1935, 1939; Qin & Phengklai 2007). In China, *Alangium kurzii* is a woodland tree occurring at 600–1600 m a.s.l. in mixed deciduous-evergreen broadleaved and conifer forests (Qin & Phengklai 2007) in fully humid to winter-dry temperate climates with hot summers (*Cfa, Cwa*; meridio-nemoral vegetation element; File S1) with mean annual temperatures ranging from 11.7 to 24.8 °C, an annual precipitation of 711 to 2435 mm, and coldest month mean temperatures of 1.1 to 20.1 °C (Fang et al. 2009). Based on the preferred habitat of extant *Alangium kurzii*, and the rareness of this fossil pollen type, it is likely that the Lavanttal *Alangium* was not growing in the lowland wetlands, but was part of the forest community surrounding the basin, and probably growing along streams or in sparse forest units on well-drained substrates.

*Genus* Cornus *L.*

Cornus *sp.*

(*Figure 12A–C*)

**Figure 12.**
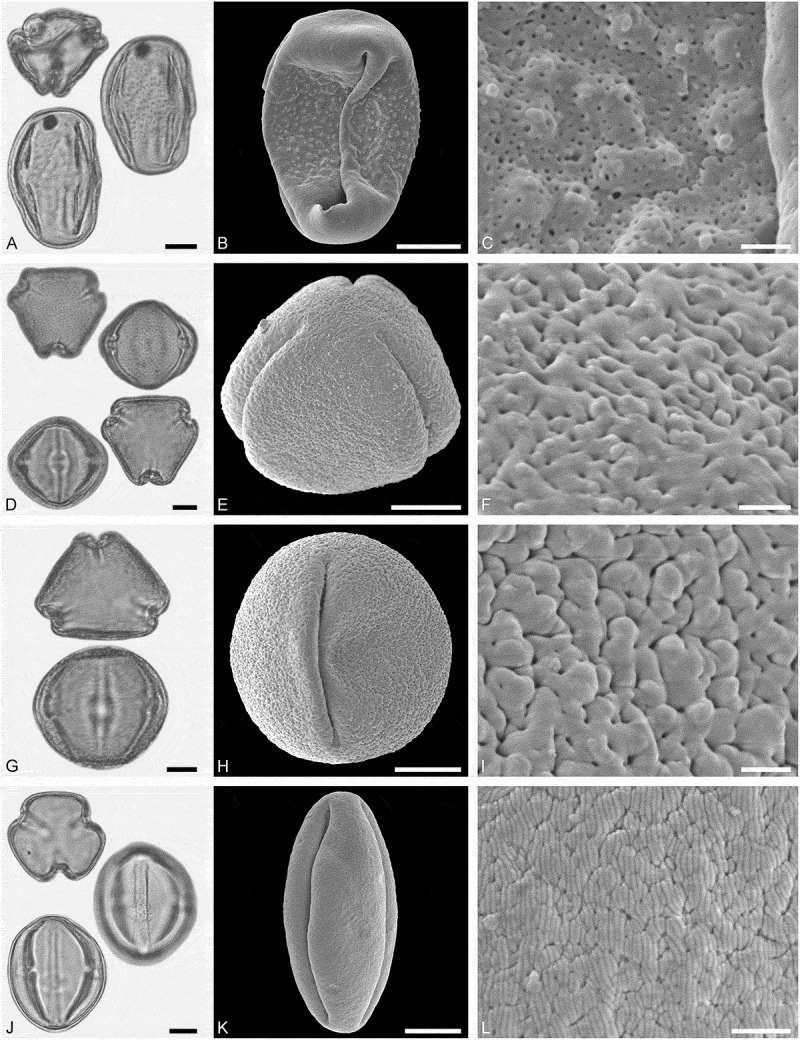
Light microscopy (LM) (**A, D, G, J**) and scanning electron microscopy (SEM) (**B, C, E, F, H, I, K, L**) micrographs of dispersed fossil Cornaceae and Ebenaceae pollen. **A–C.** *Cornus* sp., close-up of sculpture in area of mesocolpium. **D–F.** *Nyssa* sp., close-up of sculpture in area of central mesocolpium. **G–I.** *Nyssa* sp., close-up of sculpture in area of central mesocolpium. **J–L.** *Diospyros* sp., close-up of microrugulate sculpture in area of central mesocolpium. Scale bars 10 µm (A, B, D, E, G, H, J, K), 1 µm (C, F, I, L).

##### Description

Pollen, monad, prolate, outline elliptic in equatorial view, convex triangular in polar view; polar axis 44–45 µm long in LM, 38–39 µm long in SEM, equatorial diameter 29–30 µm wide in LM, 25–26 µm wide in SEM; tricolporate, endopori lalongate; exine 1.1–1.2 µm thick (LM), nexine thinner than sexine, sexine thickened along colpi; tectate; sculpture psilate to scabrate in LM, microechinate, perforate in SEM, perforations closely spaced, polar areas and margins around colpi psilate with irregularly and widely distributed perforations (SEM).

##### Remarks

The pollen morphology and ultrastructure of *Cornus* has been studied by Ferguson (1977). For additional LM- and SEM-based work on *Cornus* pollen see e.g. Adams and Morton (1976), Stafford and Heath (1991), Chester and Raine (2001), Perveen and Qaiser (2002), Mert (2009), Li et al. (2011), Miyoshi et al. (2011), and Kilie and Tuttu (2017). In his study on *c*. 44 different *Cornus* species, Ferguson (1977) concluded that all taxa produce similar pollen (stenopalynous genus *sensu* Halbritter et al. 2018) but divided them into two groups: (a) ‘*Cornus mas*-subtype’, and (b) ‘*Cornus sanguinea*-subtype’. The main difference between these two subtypes is the polar/equatorial (P/E) ratio. Pollen assigned to the *Cornus mas*-subtype are ± isodiametric, and those grouped in the *C*. *sanguinea*-subtype are ± prolate.

##### Fossil record

The earliest fossil attributed to *Cornus* is a permineralised fruit, *Cornus* cf. *piggae* Manchester, Wing et Xiang, from the late Campanian (*c*. 73 Ma) of Vancouver Island, British Columbia, Canada (Atkinson et al. 2016). Fossil *Cornus* fruits are also known from the early Cainozoic, including *C. piggae* from the Paleocene of North Dakota, USA (Manchester et al. 2010), *C. ettingshausenii* (Gardner) Eyde and *C. multilocularis* (Gardner) Eyde from the early Eocene of southern England (Eyde 1988). Other early Cainozoic records are based on leaves and include *C. swingii* Manchester, Xiang, Kodrul et Akhmetiev from the Paleocene of the United States (Manchester et al. 2009b), *Cornus hyperborean* Heer from the Paleocene of Atanikerluk, Nuussuaq Peninsula, West Greenland (Heer 1869), *Cornophyllum hebridicum* (Johnson) Boulter et Kvaček from the Paleocene of Mull, Scotland (Boulter & Kvaček 1989), *C*. *platyphylla* Saporta from the Paleocene of Sézanne, France (Saporta 1868), and *C. krassilovii* Manchester, Xiang, Kodrul et Akhmetiev from the Paleocene of Amur, Russian Far East (Manchester et al. 2009b). The complete post-Paleocene fossil record of *Cornus* was summarised in detail by Eyde (1988), including the alleged pollen record. Both Eyde (1988) and Muller (1981) were sceptic about all the fossil *Cornus* pollen that had been published/described (using LM only) at that time, stating they lack diagnostic features to differentiate them from pollen grains of other Cornaceae and related groups. The earliest fossil *Cornus* pollen studied using SEM is reported from the late Paleocene of Almont, North Dakota, USA (Zetter et al. 2011).

##### Ecological implications

The fossil *Cornus* pollen from Lavanttal definitely falls into the *C*. *sanguinea*-subtype. Extant taxa producing such pollen are distributed across most of the Northern Hemisphere. These include the American *C. alternifolia* L.f. (central and eastern Canada to north central and eastern United States), *C. amomum* Mill. (east central and eastern United States), *C. asperifolia* Michx. (southeast United States), *C. drummondii* C.A.Mey. (south-eastern Canada to central and east central United States), *C*. *excelsa* Kunth (Mexico to Honduras), *C. foemina* Mill. (east central and south-eastern United States), *C. glabrata* Benth. (southern Oregon to California, USA), C. *peruviana* J.F.Macbr. (Costa Rica to north-western Venezuela and Bolivia), *C. rugose* Lam. (central and eastern Canada to north central and eastern United States) and, *C. sericea* L. (widespread), the Eurasian *C. sanguinea* L. (Europe to Lebanon) and *C. alba* L. (north-eastern Europe to northern Korea), and the East Asian *C. controversa* Hemsl. (central Himalaya to southern Kuril Island), *C. hemsleyi* C.K.Schneid. et Wangerin (China), *C. quinquenervis* Franch. (central and southern China), *C. ulotricha* C.K.Schneid (Tibet to central China), *C. walteri* Wangerin (China to Korea), *C. wilsoniana* Wangerin (central and southern China) (distribution data from WCSP 2019). Plants producing the *C*. *sanquinea*-subtype pollen are mostly deciduous shrubs to small trees (up to 20 m tall). Depending on their geographic distribution, taxa occuring from sea-level to *c*. 3600 m a.s.l. (Xiang & Boufford 2005; Murell & Poindexter 2016). The Chinese *Cornus* are part of various, different vegetation units and forests. These include mixed broad-leaved and conifer forests, mixed thickets/woods, dense forests, the margin of woods, and scrub vegetation. *Cornus* is frequently growing along streams in both lowlands and at higher elevations, on hillsides and in real mountainous regions (Xiang & Boufford 2005). In China, most *Cornus* species occur in fully humid warm temperate climates with hot summers or winter-dry warm temperate climates with hot to warm summers (*Cfa, Cwa, Cwb*; meridio-nemoral vegetation element; File S1). Some *Cornus* extent into winter-dry snow climates with hot to warm summers or cool summers and cold winters (*Dwa, Dwb, Dwc*; nemoral vegetation element; File S1). In North America, *Cornus* grow from sea-level to *c*. 3400 m a.s.l. *Cornus* plants are found in both open vegetation as well as in dense forest, in mesic or dry-mesic deciduous hardwood forests, alluvial woods, or dry woodlands. As in China, *Cornus* in America often occurs along river and stream banks, as well as near wet meadows, swamp margins and marshes (Murell & Poindexter 2016). North American *Cornus* species occur in fully humid warm temperate or snow climates with hot or warm summers (*Cfa, Cfb, Dfa, Dfb*; nemoral vegetation element; File S1). Based on present-day *Cornus*, the Lavanttal *Cornus* pollen represents a nemoral or meridio-nemoral vegetation element originating from plants growing in the lowlands, at the boundary of swamps or on levees, or from plants growing along streams in highland areas surrounding the basin.

*Genus* Nyssa *L.*

Nyssa *sp.*

(*Figure 12D–I*)

##### Description

Pollen, monad, spheroidal, outline triangular in polar view, circular in equatorial view; polar axis 41–42 µm long in LM, 34–37 µm long in SEM, equatorial diameter 39–45 µm wide in LM, 34–36 µm wide in SEM; tricolporate, endopori thickened in corners where crossing colpi (LM); exine 1.1–1.5 µm thick, nexine thinner or as thick as sexine (LM); tectate, sculpture scabrate in LM, rugulate, fossulate, perforate, microechinate in SEM, microechini widely and irregularly spaced, rugulae partly fused along colpi (SEM).

##### Remarks

Pollen of *Nyssa* has been studied in detail by various authors using both LM (Sohma 1963, 1967) and SEM (Saito et al. 1992; Göschl 2008; Li et al. 2011). *Nyssa aquatica* L., *N. ogeche* W.Bartram ex Marshall, *N. sylvatica* Marshall and *N. sinensis* Oliv. produce pollen with less sculptured or psilate areas surrounding the colpi. In *N. javanica* (Blume) Wangerin and *N. bifida* Craib a distinct nanorugulate sculpture is present in areas adjacent to the colpi (Göschl 2008). Information concerning this character state in *N. talamancana* Hammel et N.Zamora is currently unavailable. All investigated *Nyssa* pollen from Lavanttal displayed no nanorugulation in the colpus area.

##### Fossil record

The fossil record of *Nyssa* has been summarised by Eyde (1997) and Manchester et al. (2015). Early macrofossil records of Nyssoideae (fruits) date back to the Upper Cretaceous (early Coniacian) of Japan (Takahashi et al.  2002). The three sculpture types known from endocarps of extant species (ridged with sunken bundles, ridge raised bundles, and smooth) have been reported from the early Eocene of North America and Europe, indicating radiation prior to the Eocene. The form-genera *Nyssapollenites* Thiergart ex R.Potonié and *Nyssoidites* R.Potonié, Thomson et Thiergart ex R.Potonié have commonly been used for fossil *Nyssa*-like pollen. The Cainozoic palynological record of *Nyssa* is summarised by Muller (1981) and Eyde (1991), and most recently by Stuchlik et al. (2014), accepting earliest records from the Paleocene of the Northern Hemisphere.

##### Ecological implications

The nine extant species of *Nyssa* show a disjunct distribution, with five species occurring in eastern North America (*N. aquatica, N. biflora* Walter, *N. ogeche, N. sylvatica, N. ursina* Small), one in Central America (*N. talamancana*), and three in east to southeast Asia (*N. bifida, N. javanica, N. sinensis*) (Wen & Stuessy 1993; Tucker 2016; POWO 2017). All extant species of *Nyssa* are deciduous trees. The North American species are commonly found in habitats with water-logged soils (swamps, floodplain forests, riparian forests) at low elevations in fully humid warm temperate climate (*Cfa*). *Nyssa sylvatica* shows the widest ecological range, thriving in wet, well-drained, and even dry environments/habitats, from subtropical to snow climates (*Aw, Cfa, Cfb, Dfa, Dfb*; meridio-nemoral vegetation element; File S1), at an elevation from sea level up to 1100 (1600) m, and frost resistance in mature trees to −30 °C (Sakai & Weiser 1973; Tucker 2016). *Nyssa talamancana* thrives at middle elevations in Costa Rica and Panama under fully humid equatorial climate or fully humid warm temperate climate with warm summers (*Af, Cfb*; tropical-oreotropical vegetation element; File S1) together with other relict genera, e.g. *Ticodendron, Alfaroa, Oremunnea* and *Gordonia* (Hammel & Zamora 1990). *Nyssa sinensis* is found in wet mixed forests along streams at elevations from 300 to 2700 m (Haining & Chamlong 2007), under fully humid to winter-dry warm temperate climate with hot to warm summers (*Cfa, Cfb, Cwa, Cwb*; meridio-nemoral vegetation element: File S1). The habitats of extant *Nyssa* species producing pollen similar to the fossil from Lavanttal point to a nemoral to meridio-nemoral vegetation element. Its pollen probably originated from plants growing in the lowlands, in swamps, on waterlogged soils, or as riparian elements.

Order Ericales Bercht. et J.Presl

Family Ebenaceae Gürke

Genus *Diospyros* L.

Diospyros *sp.*

(*Figure 12J–L*)

##### Description

Pollen, monad, prolate, outline elliptic in equatorial view, trilobate in polar view; polar axis 41–43 µm long in LM, 40–42 µm long in SEM, equatorial diameter 34–36 µm wide in LM, 21–26 µm wide in SEM; tricolporate, pori lalongate, colpi long and bowlike (LM); exine 1.0–1.3 µm thick, nexine thinner than sexine (LM); tectate; sculpture psilate in LM, microrugulate, perforate, fossulate in SEM, rugulae in small groups, rugulae parallel and oriented within groups (SEM).

##### Remarks

The pollen morphology (LM, SEM) and ultrastructure (TEM) of *Diospyros* has been studied thoroughly by, among others, Erdtman (1952), Ng (1971), Cerceau-Larrival et al. (1984), de Franceschi (1993), Morton (1994), Tissot et al. (1994), Kodela (2006), Geeraerts et al. (2009), Sánchez-Dzib et al. (2009), Grygorieva et al. (2010, 2013), Li et al. (2011), Miyoshi et al. (2011), and Gosling et al. (2013). The recent most comprehensive SEM work by Geeraerts et al. (2009) defines four main sculpture types within Ebenoideae (including *Diospyros, Euclea, Royena*). The sculpture of the fossil pollen type from Lavanttal falls within ‘Type 1b’ of Geeraerts et al. (2009) that occurs in various extant *Diospyros*, but also *Euclea* and *Royena* species. According to Geeraerts et al. (2009) pollen of the three genera can be distinguished based on pollen and orbicule size, equatorial outline, and sculpture. Pollen of extant *Diospyros* species display a wide range in size and outline, but all have a (micro)rugulate sculpture (Geeraerts et al. 2009). The relatively long colpi and perforations observed in interapertural areas (SEM) of the fossil pollen is shared with pollen of many extant *Diospyros* taxa (Geeraerts et al. 2009, tables I and II).

##### Fossil record

The macrofossil record of *Diospyros* has been summarised by Hiern (1873), Berry (1912), and Basinger and Christophel (1985). Both Basinger and Christophel (1985) and Wallnöfer (2001, 2004) agree that most of the Cretaceous to late Cainozoic leaf records are questionable and need to be re-evaluated. The most convincing records are fossil flowers, *Austrodiospyros cryptostoma* Basinger et Christophel, with *in situ* pollen and leaves from the middle Eocene of southern Australia. Both leaves and pollen of this fossil taxon are identical to those found in extant *Diospyros* (Christophel & Basinger 1982; Basinger & Christophel 1985). As in the case of leaves, the dispersed fossil pollen of *Diospyros* is hard to identify and distinguish from pollen of many Sapotaceae and Styracaceae when studied using LM only (cf. Erdtman 1952). The only reliable pollen records of *Diospyros* are those verified using SEM. Fossil *Diospyros* pollen identified using SEM have been described from the middle Eocene of Hainan, China (Hofmann 2018), and the late Eocene of Colorado, USA (Bouchal et al. 2016a).

##### Ecological implications

*Diospyros* is pantropical and comprises between 500 and 600 species, growing mostly in lowland tropical to subtropical regions. Only a few species are known to extend into mountains and temperate regions. There are about 100 different species in the Americas, *c*. 95 in Africa, *c*. 95 in Madagascar and the Comoro Islands, 200–300 in Asia and the Pacific area, and about 15 in Australia (Wallnöfer 2001, 2004). *Diospyros* are mostly small to medium-sized evergreen, less frequently deciduous, trees in the forest understorey. The trees often thrive along rivers but can also be found in swamps and periodically flooded environments. Some species are part of well-drained forests and occur in deciduous forests (e.g. *D. lotus* L., *D. kaki* L.f., *D. virginiana* L.); some are even growing in fire-prone savannahs (Wallnöfer 2001, 2004). In North America, *D. virginiana*, is a widely distributed tree (up to 40 m tall) in the eastern United States. It occurs from 0 to 1100 m a.s.l. and is found in various forest types, from seasonally flooded bottomlands to dry ridgetops (Eckenwalder 2009), predominantly thriving in fully humid warm temperate climates with hot to warm summers (*Cfa*; meridio-nemoral vegetation element; File S1) and extending into *Dfa* climates with coldest month minima of −4.9° (Thompson et al. 1999). The other North American taxon, *D. texana* Scheele, distributed in Texas and Mexico, occurs from 0 to 1800 m a.s.l., and is part of open lowland bottomlands, prairie margins, and is found on rocky hillsides. In Texas, it thrives under fully humid warm temperate climate with hot summers (*Cfa*), but in the southern part of its distribution area, Mexico, it is found in hot arid steppe climate (*BSh*; File S1). China, mostly its south-eastern and south-western parts, is home to about 60 species of *Diospyros* (Lee et al. 1996). Small shrubs to large trees (up to *c*. 25 m tall), which can be either deciduous or evergreen, which occur from sea level to an elevation of 2700 m. The plants are part of both deciduous and broad-leaved evergreen forests, as well as mixed broad-leaved evergreen-deciduous forests. They often occur in forested ravines or on slopes, and in forests beside streams or moist lowland valley forests (Lee et al. 1996). The Chinese *Diospyros* fall into two major groups regarding climate preferences. About one-third of the taxa grow under fully humid to winter-dry warm temperate climate with hot to warm summers (*Cfa, Cfb, Cwa, Cwb*; nemoral to meridio-nemoral vegetation element; File S1). About two-thirds of the species either extend into (tropical-meridional vegetation element) or are confined to various equatorial climates (tropical vegetation element), like fully humid rainforest (*Af*), monsoon (*Am*), or savannah climates with dry winter (*Aw*; File S1). Based on the extant habitats of warm temperate *Diospyros* species, the fossil pollen from Lavanttal most likely represent shrubs or small trees, characteristic for the understory. The pollen could originate from plants growing in the lowland wetlands or along streams at the periphery of the basin. It could also derive from plants growing further away from the accumulation zone, representing ravine vegetation or a highland element.

Family Ericaceae Juss.

*Genus* Andromeda *L.*

Andromeda *sp.*

(*Figure 13A–C*)

**Figure 13.**
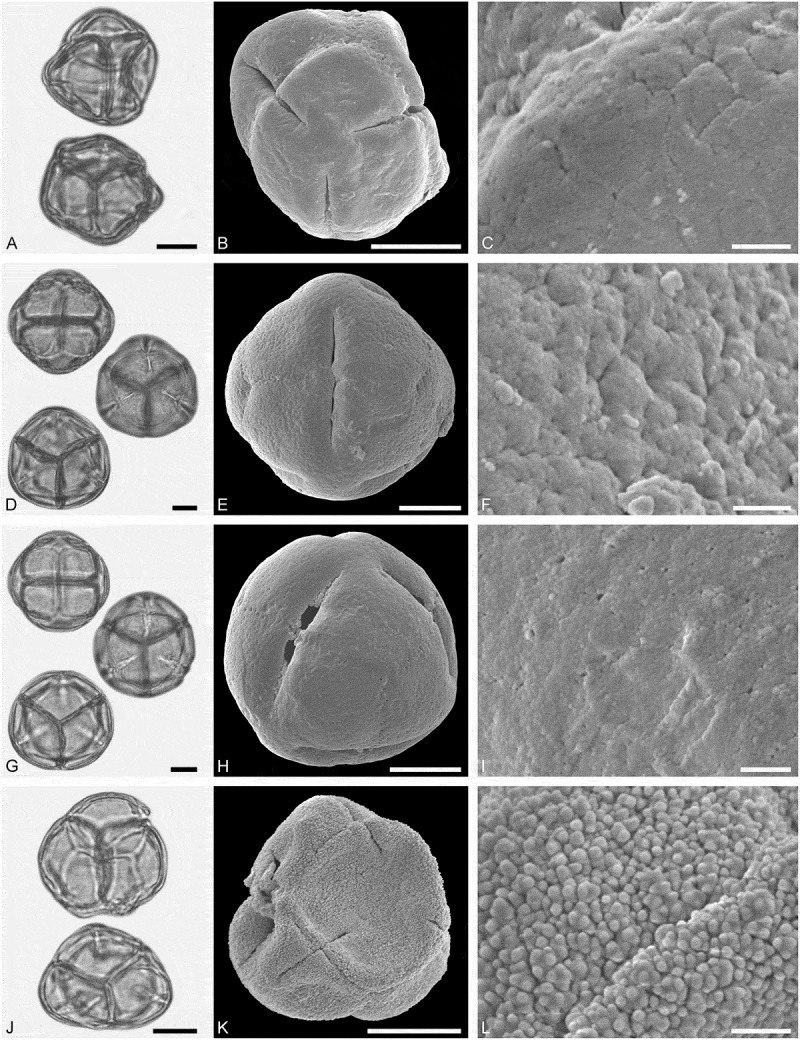
Light microscopy (LM) (**A, D, G, J**) and scanning electron microscopy (SEM) (**B, C, E, F, H, I, K, L**) micrographs of dispersed fossil Ericaceae pollen tetrads. **A–C.** *Andromeda* sp., close-up of microrugulate and fossulate sculpture in polar area. **D–F.** *Arbutus* sp., close-up of granulate and fossulate sculpture in area of mesocolpium. **G–I.** *Arbutus* sp., close-up of sculpture in polar area. **J–L.** *Empetrum* sp., close-up showing microverrucate sculpture with nanoechinate suprasculpture. Scale bars 10 µm (A, B, D, E, G, H, J, K), 1 µm (C, F, I, L).

##### Description

Permanent tetrads, subspheroidal, outline convex triangular in polar view, rounded quadrangular in equatorial view; diameter of tetrad 27–31 µm wide in LM, 26–28 µm wide in SEM; pollen tricolporate; exine 1.3–1.5 µm thick (LM); tectate; sculpture psilate in LM, microrugulate, granulate, fossulate, perforate in SEM, fossulae more pronounced in area of pollen intersection (SEM).

##### Remarks

The tetrad morphology, size, and the delicate sculpture overserved with SEM suggests that this type belongs to *Andromeda*. Extant pollen tetrads of *A*. *polifolia* L. have been figured using both LM and SEM by several authors (e.g. Oldfield 1959; Adams & Morton 1979; Hebda 1979; Warner & Chinappa 1986; Sarwar & Takahashi 2006; Halbritter & Svojtka 2016).

##### Fossil record

It is not easy to affiliate fossil Ericaceae tetrads to extant lineages and/or genera. Most fossil records are documented using LM only and assigned to different species of the form-genus *Ericipites* Wodehouse (e.g. Stuchlik et al. 2014). We are unaware of any convincing pre-Quaternary *Andromeda* macrofossil or pollen record.

##### Ecological implications

*Andromeda polifolia* is the single modern species of genus *Andromeda* (POWO 2017). It is a low growing evergreen shrub that is usually 10 to 25 cm tall but can grow up to 40 cm. This species is present in all circumboreal regions of the Northern Hemisphere. It inhabits wet sites, (peat) bogs, fens, swamps, margins of pools, and boggy shores, from sea level up to 1500 m, throughout boreal forests and the Arctic (Jacquemart 1998; Fabijan 2009). For a detailed summary of the distribution and the ecology of *A. polifolia* see Jacquemart (1998). *Andromeda polifolia* does not extend into tropical (*A*) or arid (*B*) climates but is found in fully humid warm temperate and snow climates with hot to cold summers (*Cfa, Cfb, Cfc, Dfb, Dfc*; File S1), summer- and winter-dry snow climates (*Dwb, Dwc, Dsb, Dsc*) and Tundra climates (*ET*) (boreal to nemoral vegetation element; File S1). During the Miocene of Lavanttal *Andromeda* probably was part of the lignite forming swamp community or growing at the margins of conifer forests belts at higher elevation.

*Genus* Arbutus *L.*

Arbutus *sp.*

(*Figure 13D–I*)

##### Description

Permanent tetrads, subspheroidal, outline convex triangular in polar view, rounded quadrangular in equatorial view; diameter of tetrad 37–44 µm wide in LM, 34–39 µm wide in SEM; pollen tricolporate; exine 1.1–1.5 µm thick (LM); tectate; sculpture scabrate in LM, granulate, fossulate, perforate in SEM, fossulae deeper and more pronounced in area of mesocolpium, fossulae in polar areas and around colpi fewer and more shallow, colpus membrane microverrucate to microechinate (SEM).

##### Remarks

*Arbutus* has large tetrads compared to most Ericaceae. The colpi are distinct, but the boundaries between individual pollen grains (in mesocolpium area) are extremely vague (linear perforations) and untypical for this family. Individual pollen grains are ± triangular in polar view (LM) versus lobate to circular in most Ericaceae. This, in combination with the granulate and perforate sculpture observed with SEM, clearly place these fossil tetrads from Lavanttal in *Arbutus*. The pollen morphology of most *Arbutus* species have been documented in detail using both LM and SEM by e.g. Adams and Morton (1979), Lewis et al. (1983), Warner and Chinappa (1986), Barento et al. (1987), Luteyn et al. (1995), Sarwar (2007), Sarwar et al. (2008), and Halbritter (2016a).

##### Fossil record

The fossil record of *Arbutus* is meagre. In the United States, fossil *Arbutus* leaves have been reported from the early and late Oligocene of Colorado (Axelrod 1987; Gregory & McIntosh 1996), the Miocene of Nevada and Oregon (e.g. Axelrod 1991; Graham 1999), and the Miocene/Pliocene of California (Axelrod 1950). In Europe, fossil *Arbutus* leaves are known from the Eocene to Pliocene (summarised in Mai 1995b). Noteworthy is that genetic investigations of Arbutoideae have shown that European *Arbutus* species are sister to all American genera (including *Arbutus*) of this subfamily (Hileman et al. 2001). As mentioned previously, it is not easy to affiliate fossil Ericaceae pollen tetrads to extant lineages and/or genera, therefore, most fossil Ericaceae pollen tetrads are assigned to the form genus *Ericipites* (e.g. Stuchlik et al. 2014). There seems to be no previous convincing pre-Quaternary *Arbutus* pollen record.

##### Ecological implications

*Arbutus* is a small genus, comprising *c*. 14 species of shrubs and trees, occurring in western Europe to the Mediterranean, Macaronesia, and western Canada to Central America (Hileman et al. 2001; Sørensen 2009; POWO 2017). Four species, *A. andrachne* L., *A. unedo* L., *A*. × *andrachnoides* Link, and *A. pavarii* Pamp., occur in the Mediterranean region from North Africa to the Middle East, here they thrive under summer-dry or fully humid temperate conditions with hot to warm summers (*Csa, Csb, Cfa, Cfb*; File S1). *Arbutus canariensis* Veill. ex Duhamel and *Arbutus* × *androsterilis* Salas, Acebes et del Arco are endemic to the Canary Islands. The remaining eight taxa (*A. arizonica* [A.Gray] Sarg, *A*. *bicolor* S.González, MGonzález et P.D.Sørensen, *A. occidentalis* McVaugh et Rosatti, *A. madrensis* S.González, *A. menziesii* Pursh, *A. mollis* Kunth, *A. tessellata* P.D.Sørensen, *A. xalapensis* Kunth) all occur in the Americas (Hileman et al. 2001; Sørensen 2009; POWO 2017). *Arbutus arizonica* thrives in riverine forest and along seasonally moist waterways, at an elevation of 1500 to 2400 m, in Arizona and northern Mexico. *Arbutus menziesii* occurs in open forests, on rocky slopes, foothills, in ravines, and along shores, at an elevation of 0 to 1800 m, from British Columbia to northwest Mexico. In tropical Central America *Arbutus* is found primarily in forested montane areas dominated by *Pinus* and *Quercus* (Sørensen 2009). The American *Arbutus* species grow under a wide range of climates, from fully humid temperate conditions with warm to cool summers (*Cfb, Cfc*) to summer- or winter-dry climates (*Csb, Cwa, Cwb*), and even extend into equatorial and arid climates (*Aw, BSh, BSk*). The *Arbutus* pollen from the Miocene of Lavanttal could have originated from trees growing along streams or in ravines at the periphery of the basin, or from drier hill sites or mountain areas surrounding the lowlands.

*Genus* Empetrum *L.*

*Empetrum* sp.

(*Figure 13J–L*)

##### Description

Permanent tetrads, subspheroidal, outline convex triangular in polar view, rounded quadrangular in equatorial view; diameter of tetrad 29–31 µm wide in LM, 25–27 µm wide in SEM; pollen tricolporate; exine 0.8–1.0 µm thick (LM); tectate; sculpture psilate in LM, microverrucate in SEM, microverrucae with a nanoechinate suprasculpture, apices of microechini often blunt (SEM).

##### Remarks

The pollen morphology of *Empetrum* has been studied with both LM and SEM and figured, among others, by Warner and Chinappa (1986), Foss and Doyle (1988), Beug (2004), Sarwar (2007), Miyoshi et al. (2011), and Halbritter (2016b). The fossil pollen from Lavanttal corresponds in size and morphology to extant *Empetrum* pollen, and the sculpture observed in SEM is comparable to that of *E. nigrum* L. as depicted by Halbritter (2016b).

##### Fossil record

The few North American and European macrofossil records of *Empetrum* are mostly confined to the Quaternary (e.g. Mai 1995b; Graham 1999). Still, a single endocarp assigned to this genus is reported from the middle Miocene of Denmark (Friis 1979). According to Mai (1995b), *Empetrum* pollen is first recovered from the Miocene of Europe, but then became a prominent component in Pliocene and younger peat deposits of that region. As noted earlier, Ericaceae pollen tetrads are very similar in morphology and it is not easy to affiliate fossil tetrads to extant taxa unless observed with SEM. Most fossil Ericaceae tetrads studied using LM are assigned to the form-genus *Ericipites* (e.g. Stuchlik et al. 2014). There are currently no previous pre-Miocene *Empetrum* pollen records verified using SEM.

##### Ecological implications

*Empetrum* composes about four extant species of prostrate shrubs (Murray et al. 2009; POWO 2017). Three of the species are restricted in distribution. *Empetrum rubrum* Vahl ex Willd. is native to southern South America and the Falkland Islands, where it thrives under fully humid warm temperate climates, with warm to cool summers and cold winters, and extends into summer-dry as well as tundra climates (*Csb, Cfb, Cfc, ET*; File S1). *Empetrum atropurpureum* Fernald et Wiegand and *E. eamesii* Fernald et Wiegand are native to eastern Canada and north-eastern United States, where they occur at an elevation from sea level to *c*. 1500 m, on dunes, sandy terraces, coastal rock barrens, alpine heath, and exposed mountain slopes near treeline (Murray et al. 2009). Both species grow under fully humid snow climates with warm to cool summers (*Dfa, Dfb, Dfc*; File S1) with *E. atropurpureum* extending into fully humid temperate climates (*Cfb*) touching *Cfa* at the coast of Maine, USA. The most common species, *E*. *nigrum*, has a circumpolar (temperate to Arctic) northern hemispheric distribution (POWO 2017). According to Murray et al. (2009), *E. nigrum* occurs in North America, Greenland and Europe at elevations from sea level to *c*. 1900 m, in windswept southern arctic and alpine tundra and open subalpine and boreal forests and mountain summits, as well as on exposed, coastal bluffs and in sphagnum bogs. In China (East Asia), *E. nigrum* can be found in forests, on stony hills, at elevations of 700 to 1500 m (Fang et al. 2005). *Empetrum nigrum* does not extend into tropical (*A*) or arid (*B*) climates but is found in fully humid warm temperate and snow climates with hot to cold summers (*Cfa, Cfb, Cfc, Dfb, Dfc*; File S1), summer-dry warm temperate climate with warm summers (*Csb*; File S1), summer- to winter-dry snow climates (*Dsc, Dwb, Dwc*), and Tundra climates (*ET*) (boreal to nemoral vegetation element; File S1). It is possible that during the Miocene of Lavanttal, *Empetrum* was growing on shady slopes or in shadows at the edge of woodlands in the hillsides and/or mountains surrounding the Lavanttal Basin.

*Genus* Erica *L.*

Erica *sp. 1*

(*Figure 14A–C*)

**Figure 14.**
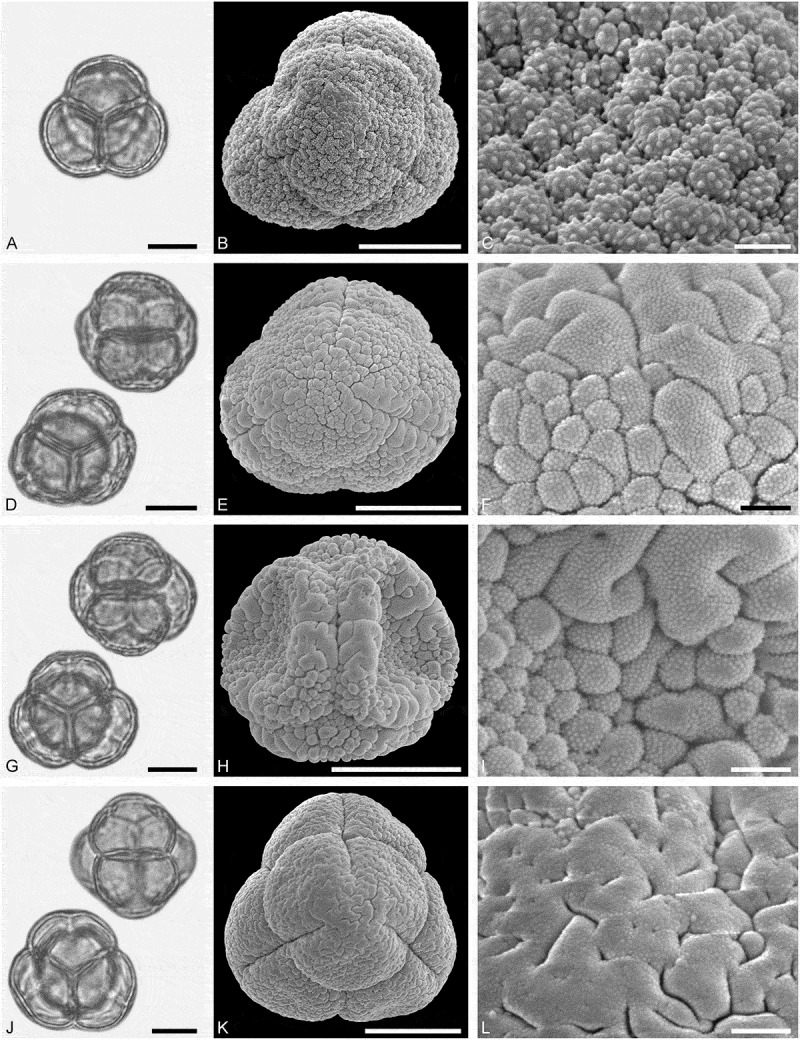
Light microscopy (LM) (**A, D, G, J**) and scanning electron microscopy (SEM) (**B, C, E, F, H, I, K, L**) micrographs of dispersed fossil Ericaceae pollen tetrads. **A–C.** *Erica* sp. 1, close-up of polar area showing (micro)verrucae with nanoechinate suprasculpture. **D–F.** *Erica* sp. 2, close-up of polar area showing (micro)verrucae and rugulae with nanoechinate suprasculpture. **G–I.** *Erica* sp. 2, close-up of sculpture in area of mesocolpium. **J–L.** *Erica* sp. 3, close-up of polar area showing rugulae with a minute-nanoechinate suprasculpture. Scale bars 10 µm (A, B, D, E, G, H, J, K), 1 µm (C, F, I, L).

##### Description

Permanent tetrads, subspheroidal, outline lobate in polar view, rounded quadrangular in equatorial view; diameter of tetrad 23–25 µm wide in LM, 22–24 µm wide in SEM; pollen tricolporate; exine 0.9–1.2 µm thick (LM); tectate; sculpture scabrate in LM, verrucate to microverrucate in SEM, verrucae with a nanoechinate suprasculpture (SEM).

##### Remarks

Pollen in permanent tetrads showing verrucate to microverrucate sculpture and nanoechinate suprasculpture (SEM) are typical for *Erica*. Despite the number of publications including *Erica* pollen, only a small portion, less than 100 species, are palynologically studied so far (e.g. Oldfield 1959; Foss & Doyle 1988; Díez & Fernandes 1989; Mateus 1989; Oliver 2000; Sarwar 2007; Sarwar & Takahashi 2014; Wrońska-Pilarek et al. 2018; and references cited therein). Based on the taxa studied so far, *Erica* pollen can be divided into two major groups: (1) pollen dispersing in permanent tetrahedral tetrads (majority), and (2) pollen dispersing as monads (minority). All the *Erica* type pollen from Lavanttal belong to the former. Because of the numerous unstudied extant taxa and the overlapping pollen morphology of the studied ones, we refrain from affiliating the Lavanttal pollen tetrads to any particular species group or intrageneric lineage.

##### Fossil record

According to Mai (1995b, p. 182), the few pre-Quaternary leaf records of Ericoideae/*Erica* are doubtful and not based on reliable leaf anatomy nor cuticle analyses. Rare fruits/capsules of *Erica* are documented from the late Miocene of Europe (van der Burgh 1987). As with other fossil Ericaceae pollen tetrads, they are hard to affiliate to extant lineages and/or genera unless studied with SEM. Therefore, most fossil *Erica* tetrads are lumped into the form-genus *Ericipites* (e.g. Stuchlik et al. 2014). The earliest pollen records of *Erica*, verified using combined LM and SEM, are from the early Miocene of Europe, but they become common elements in late Miocene and younger sediments of that region (e.g. Zetter 1991; Ferguson et al. 1998; van der Burg & Zetter 1998; Hofmann et al. 2002).

##### Ecological implications

*Erica* is one of the largest plant genera, comprising *c*. 858 species (POWO 2017) of perennial woody prostrate shrubs and trees, up to 10 m tall (Oliver 2000; Stevens et al. 2004). *Erica* species show a narrow north–south geographic distribution, spanning north-western Europe and the Mediterranean region (*c*. 23 spp.), the Middle East (the genus extends eastwards into Turkey and Lebanon and the south-western part of the Arabian Peninsula), eastern and soutern Africa (*c*. 780 spp.), and Madagascar and the Mascarene Islands (*c*. 50 spp.). *Erica arborea* L. is the only species that is widespread in both Europe and Africa. *Erica arborea* is present in fully humid to summer-dry temperate (*Cfa, Cfb, Csa, Csb*; File S1) climates in Europe. In Africa, this species is found in winter-dry to fully humid temperate climates in mountainous regions (*Cfb, Cwa, Cwb*; File S1). In Europe, *Erica* are part of different shrub communities, and in Africa they are part of montane scrub or grasslands (Quezel 1978; Oliver 2000; McGuire & Kron 2005). In general, *Erica* thrives under winter-dry, summer-dry, and fully humid temperate climates with hot or warm summers (*Cfa, Cfb, Csa, Csb, Cwa, Cwb*; File S1), as well as fully humid snow climates with warm to cool summers (*Dfb, Dfc*), but rarely extends into equatorial (*A*) or arid (*B*) climates. The fossil *Erica* pollen tetrads from Lavanttal either originate from small shrubs that occurred in open habitats (dry to wet substrate, lowland to highland) or as understory growth (ground vegetation) in woodlands.

Erica *sp. 2*

(*Figure 14D–I*)

##### Description

Pollen, tetrad, subspheroidal, outline lobate in polar view, rounded quadrangular in equatorial view; diameter of tetrad 22–26 µm wide in LM, 19–23 µm in SEM; pollen tricolporate; exine 0.8–1.0 µm thick (LM); tectate; sculpture psilate in LM, verrucate to microverrucate in SEM, (micro)verrucae fused around colpi forming rugulae, (micro)verrucae and rugulae with a nanoechinate suprasculpture, nanoechini numerous and densely packed (SEM).

##### Remarks

The *Erica* sp. 2 pollen tetrads are easily distinguished from the *Erica* sp. 1 type. In *Erica* sp. 2 the verrucae around the colpi are fused and forming ‘large’ rugulae, a feature not observed in *Erica* sp. 1. Also, the nanoechini in *Erica* sp. 2 are much smaller and more numerous than in *Erica* sp. 1. A similar ornamented *Erica* pollen tetrad is reported from the middle Miocene of Turkey (Bouchal et al. 2016b, figure 20G–I).

Erica *sp. 3*

(*Figure 14J–L*)

##### Description

Pollen, tetrad, subspheroidal, outline lobate in polar view, rounded quadrangular in equatorial view; diameter of tetrad 25–28 µm wide in LM, 23–25 µm wide in SEM; pollen tricolporate; exine 0.9–1.1 µm thick (LM); tectate; sculpture psilate in LM, rugulate in SEM, rugulae larger and more elongated in polar areas and around colpi, rugulae with a minute-nanoechinate suprasculpture (SEM).

##### Remarks

The *Erica* sp. 3 pollen tetrads are similar to those of *Erica* sp. 2, but are slightly larger, the rugulae are more homogeneous in size and relief, and the nanoechini are also much shorter and blunt.

Erica *sp. 4*

(*Figure 15A–C*)

**Figure 15.**
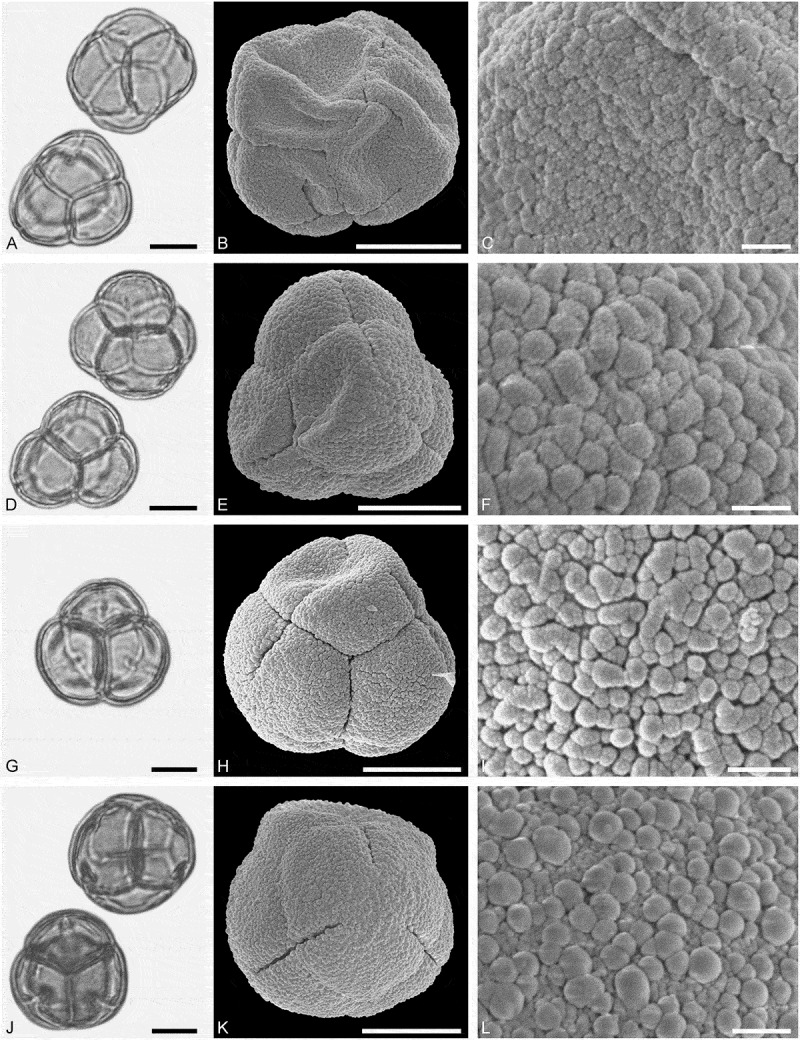
Light microscopy (LM) (**A, D, G, J**) and scanning electron microscopy (SEM) (**B, C, E, F, H, I, K, L**) micrographs of dispersed fossil Ericaceae pollen tetrads. **A–C.** *Erica* sp. 4, close-up showing nanoverrucae with nanoechinate suprasculpture. **D–F.** *Erica* sp. 5, close-up of polar area showing nanoverrucae with a minute-nanoechinate suprasculpture. **G–I.** *Erica* sp. 5, close-up of sculpture in area of mesocolpium. **J–L.** *Erica* sp. 5, close-up of polar area. Scale bars 10 µm (A, B, D, E, G, H, J, K), 1 µm (C, F, I, L).

##### Description

Pollen, tetrad, subspheroidal, outline convex triangular to lobate in polar view, rounded quadrangular in equatorial view; diameter of tetrad 25–28 µm wide in LM, 23–25 µm wide in SEM; pollen tricolporate; exine 0.8–0.9 µm thick (LM); tectate; sculpture psilate in LM, nanoverrucate in SEM, nanoverrucae with nanoechinate suprasculpture, nanoverrucae slightly larger around colpi (SEM).

##### Remarks

The *Erica* sp. 4 pollen tetrads are the only fossil *Erica* tetrads with clearly uniform nanoverrucate sculpture in SEM.

Erica *sp. 5*

(*Figure 15D–L*)

##### Description

Pollen, tetrad, subspheroidal, outline lobate in polar view, rounded quadrangular in equatorial view; diameter of tetrad 25–29 µm wide in LM, 23–26 µm wide in SEM; pollen tricolporate; exine 0.8–1.1 µm thick (LM); tectate; sculpture psilate to scabrate in LM, nanoverrucate in SEM, nanoverrucae with a minute-nanoechinate suprasculpture, nanoverrucae slightly larger around colpi (SEM).

##### Remarks

Pollen tetrads assigned to *Erica* sp. 5 differ from the remaining *Erica* sp. 1–4 in the sculpture observed with SEM. The tetrads are nanoverrucate in *Erica* sp. 5, but verrucate to microverrucate or rugulate in *Erica* sp. 1 and sp. 2. The sculpture elements around the apertures and is areas of mesocolpium in *Erica* sp. 5 are of similar size and outline but vary substantially in *Erica* sp. 2. The *Erica* sp. 5 pollen tetrads are most similar to *Erica* sp. 4 but the nanoverrucae are larger in *Erica* sp. 5, taller, and more globular in outline, and with a higher number of nanoechini per nanoverruca.

Ericaceae gen. et spec. indet. 1

(*Figure 16A–I*)

**Figure 16.**
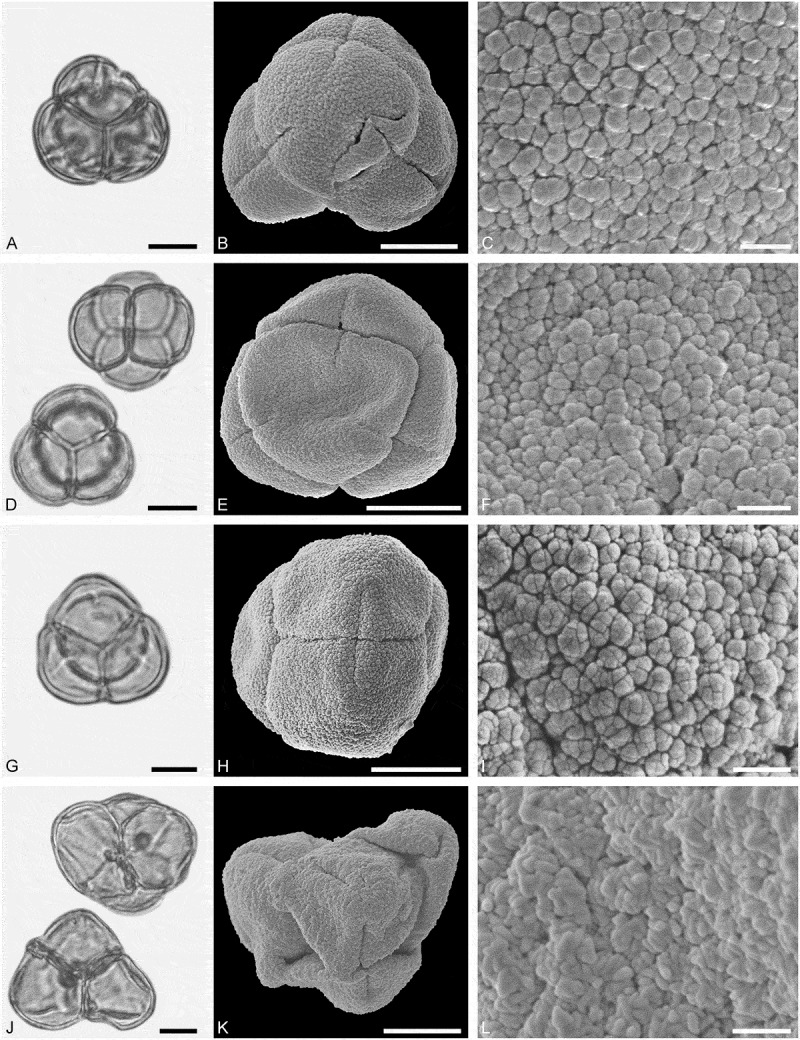
Light microscopy (LM) (**A, D, G, J**) and scanning electron microscopy (SEM) (**B, C, E, F, H, I, K, L**) micrographs of dispersed fossil Ericaceae pollen tetrads. **A–C.** Ericaceae gen. et spec. indet. 1, close-up of polar area showing microverrucae composed of rod-like elements. **D–F.** Ericaceae gen. et spec. indet. 1, close-up of polar area. **G–I.** Ericaceae gen. et spec. indet. 1, close-up of sculpture in area of mesocolpium. **J–L.** Ericaceae gen. et spec. indet. 2, close-up showing microrugulate sculpture in polar area. Scale bars 10 µm (A, B, D, E, G, H, J, K), 1 µm (C, F, I, L).

##### Description

Pollen, tetrad, subspheroidal, outline lobate in polar view, rounded quadrangular in equatorial view, diameter of tetrad 24–29 µm wide in LM, 24–28 µm wide in SEM; pollen tricolporate; exine 0.9–1.2 µm thick (LM); tectate; sculpture psilate to scabrate in LM, microverrucate in SEM, microverrucae composed of rod-like elements, rod-like elements sometimes divided into shorter units (SEM).

##### Remarks

Pollen tetrads of this and the following type definitely belong to the Ericaceae, but we are currently unable to affiliate them with certainty to a particular genus. They could also represent extinct lineages (e.g Kowalski & Fagúndez 2017).

Ericaceae gen. et spec. indet. 2

(*Figure 16J–L*)

##### Description

Pollen, tetrad, subspheroidal, outline lobate in polar view, rounded quadrangular in equatorial view, diameter of tetrad 33–38 µm wide in LM, 31–33 µm wide in SEM; pollen tricolporate; exine 0.9–1.1 µm thick (LM); tectate; sculpture scabrate in LM, microrugulate in SEM, microrugulae often forming clusters (SEM).

Family Sapotaceae Juss.

*Genus* Pouteria *Aubl.*

Pouteria *sp. 1*

(*Figure 17A–F*)

**Figure 17.**
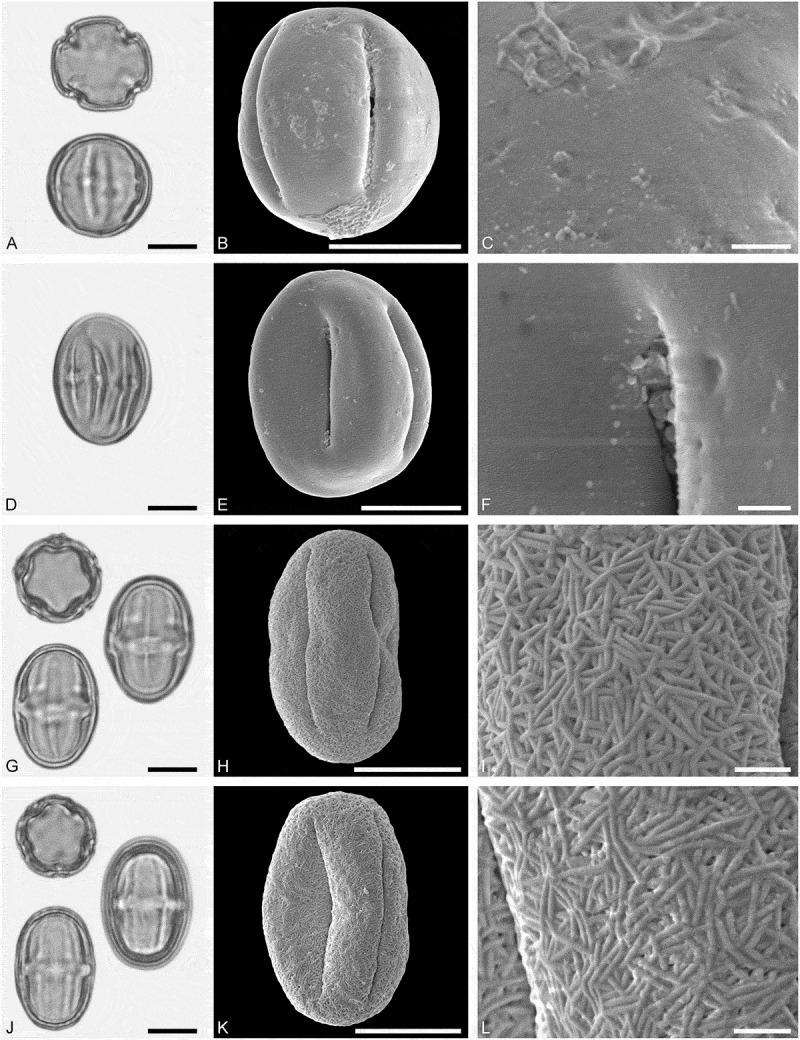
Light microscopy (LM) (**A, D, G, J**) and scanning electron microscopy (SEM) (**B, C, E, F, H, I, K, L**) micrographs of dispersed fossil Sapotaceae pollen. **A–C.** *Pouteria* sp. 1, close-up of granulate sculpture in area of mesocolpium. **D–F.** *Pouteria* sp. 1, close-up showing colpus membrane and widening at the end of colpi. **G–I.** *Pouteria* sp. 2, close-up of microrugulate to rugulate sculpture in area of mesocolpium. **J–L.** *Pouteria* sp. 2, close-up of mesocolpium. Scale bars 10 µm (A, B, D, E, G, H, J, K), 1 µm (C, F, I, L).

##### Description

Pollen, monad, prolate, outline quadrangular to lobate in polar view, elliptic in equatorial view; polar axis 20–27 µm long in LM, 17–24 µm long in SEM, equatorial diameter 19–21 µm wide in LM, 15–19 µm wide in SEM; stephano(4–5)colporate, colpi narrow, varying in length, often wider and rounded at ends, endopori small and slightly lalongate, margins of endopori thickened in corners where crossing colpi; exine 0.6–0.8 µm thick, nexine thinner than sexine (LM); tectate; sculpture psilate in LM, granulate with few perforations in SEM, colpus membrane microverrucate (SEM).

##### Remarks

The fossil *Pouteria* sp. 1 pollen falls within the range of ‘Pollen Type 8ʹ (Harley 1991a, figure 13) and ‘Subtype 8A’ (Harley 1991b, figure 35) as defined by Harley, and is similar to pollen from *Pouteria* sect. *Rivicoa* observed in *P. alnifolia* (Baker) Roberty (cf. Harley 1991b, figure 35A).

##### Fossil record

Fossil *Pouteria* pollen, identified using SEM, has been described from the middle Eocene of Eckfeld (as *Pouteria* sp., Wappler et al. 2015; Grímsson et al. 2017b) and Profen (as Sapotaceae gen. et spec. indet 6, Haring 2014), Germany.

##### Ecological implications

*Pouteria* comprises *c*. 320 species of trees and shrubs occurring in the Americas (*c*. 200 spp.), in Africa (*c*. 5 spp.), and in continental Asia, Malesia, Australia and the Pacific (*c*. 120 spp.) (Pennington 1991, 2004). *Pouteria* is a pantropical genus occurring in wet lowland (rain)forests, lowland swampy forest, (semi-)evergreen lowland forests, periodically flooded forests, upland (rain)forests, (evergreen)montane (rain)forests, riverside gallery forests, along savannah edges, and in dry thickets on limestone hills (Pennington 1990). Since *Pouteria* are pollinated by insects, the fossil pollen grains likely originated from plants growing close to the depositional site. We hence consider that the *Pouteria* pollen from Lavanttal originated from sheltered understorey shrubs (or small trees) that were part of the lowland wetland forests.

Pouteria *sp. 2*

(*Figure 17G–L*)

##### Description

Pollen, monad, prolate, outline pentangular in polar view, elliptic in equatorial view; polar axis 24–25 µm long in LM, 22–23 µm long in SEM, equatorial diameter 15–18 µm wide in LM, 12–14 µm wide in SEM; stephano(5)colporate, colpi narrow, varying in length, often wider and rounded at ends, endopori lalongate, margins of endopori thickened in corners where crossing colpi; exine 0.7–1.0 µm thick, nexine thinner than sexine (LM); tectate; sculpture psilate in LM, microrugulate to rugulate, perforate in SEM, rugulae often interwoven, colpus membrane microverrucate (SEM).

##### Remarks

The fossil *Pouteria* sp. 2 pollen falls within the range of ‘Pollen Type 9ʹ of Harley (1991a, figure 14, 1991b, figure 38) that is restricted to *Pouteria* sect. *Oxythece*, observed in *P. cuspidate* (A.DC.) Baehni, *P. gabrielensis* (Gilly ec Aubrév.) T.D. Penn., *P. pallida* (Gaertn. F.) Baehni, and *P. scrobiculata* Monachino (Harley 1991b).

*Genus* Sideroxylon *L.*

Sideroxylon *sp.*

(*Figure 18A–F*)

**Figure 18.**
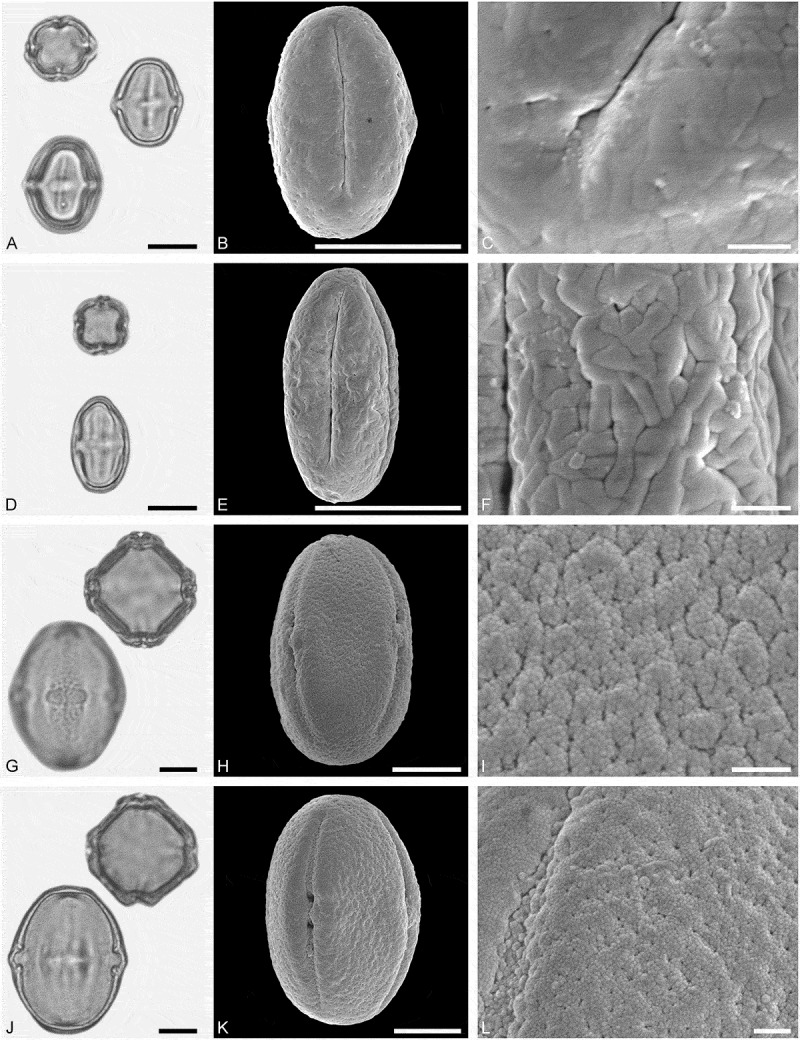
Light microscopy (LM) (**A, D, G, J**) and scanning electron microscopy (SEM) (**B, C, E, F, H, I, K, L**) micrographs of dispersed fossil Sapotaceae pollen. **A–C.** *Sideroxylon* sp., close-up of area around colpi. **D–F.** *Sideroxylon* sp., close-up showing rugulae with granulate suprasculpture in area of mesocolpium. **G–I.** Sapotaceae gen. et spec. indet. 2, close-up of sculpture in area of mesocolpium. **J–L.** Sapotaceae gen. et spec. indet. 2, close-up showing microverrucate colpus membrane. Scale bars 10 µm (A, B, D, E, G, H, J, K), 1 µm (C, F, I, L).

##### Description

Pollen, monad, prolate, outline quadrangular in polar view, elliptic in equatorial view; polar axis 18–20 µm long in LM, 13–17 µm long in SEM, equatorial diameter 11–15 µm wide in LM, 8–11 µm wide in SEM; tetracolporate, colpi long and narrow, endopori slightly rectangular, lalongate, margins of endopori perpendicular to polar axis thickened (LM); exine 0.8–0.9 µm thick, nexine thinner than sexine (LM); tectate; sculpture psilate in LM, rugulate in SEM, rugulae of varying width, rugulae with a granulate suprasculpture (SEM).

##### Remarks

The fossil *Sideroxylon* pollen falls within the range of ‘Pollen Type 6ʹ (Harley 1991a, figure 11) and ‘Subtype 6A to C’ (Harley 1991b, figure 24) as defined by Harley, and is similar to pollen from various extant *Sideroxylon* species (compare Harley 1991b, figures 24, 25).

##### Fossil record

Fossil *Sideroxylon* pollen has been described form the middle Eocene of Kassel, Hesse, Germany (as *Sideroxylon*-type, Hofmann 2018). Twigs with attached leaves and fruits (*S*. *salicites* [Weber] Weyland) are knownearly Miocene of Rott, Germany (Weyland 1937; Winterscheid 2006).

##### Ecological implications

*Sideroxylon* comprises *c*. 75 species of trees and shrubs occurring in the Americas (*c*. 49 spp.), Africa (*c*. 7 spp.), on Madagascar (6 spp.), the Mascarene Islands (8 spp.), and in Asia (*c*. 5 spp.) (Pennington 1991, 2004). *Sideroxylon* is a tropical to subtropical genus, and depending on the species, occurring among others in coastal vegetation, mangroves, wet lowland forests, (lowland) tropical rainforests, montane rain forests, cloud forests, moist seasonal evergreen forests, humid forests, lower montane forests, mixed oak-semievergreen forests, tropical (dwarf) deciduous forests, seasonal (semi)deciduous forests, dry forests, and arid thorn forests (Pennington 1990). Noteworthy is, recent genetic studies indicate that the North American clade of *Sideroxylon*, including the most frost hardy taxa, already split during the upper Eocene (Stride et al. 2014). Extant *Sideroxylon* are entomophilous (insect-pollinated) and, therefore, the fossil pollen grains from Lavanttal probably originate from plants growing close to the depositional site, from shrubs or small trees occurring in the lowland wetland forest or along streams at moderate elevation.

Sapotaceae gen. et spec. indet. 1

(*Figure 19A–L*)

**Figure 19.**
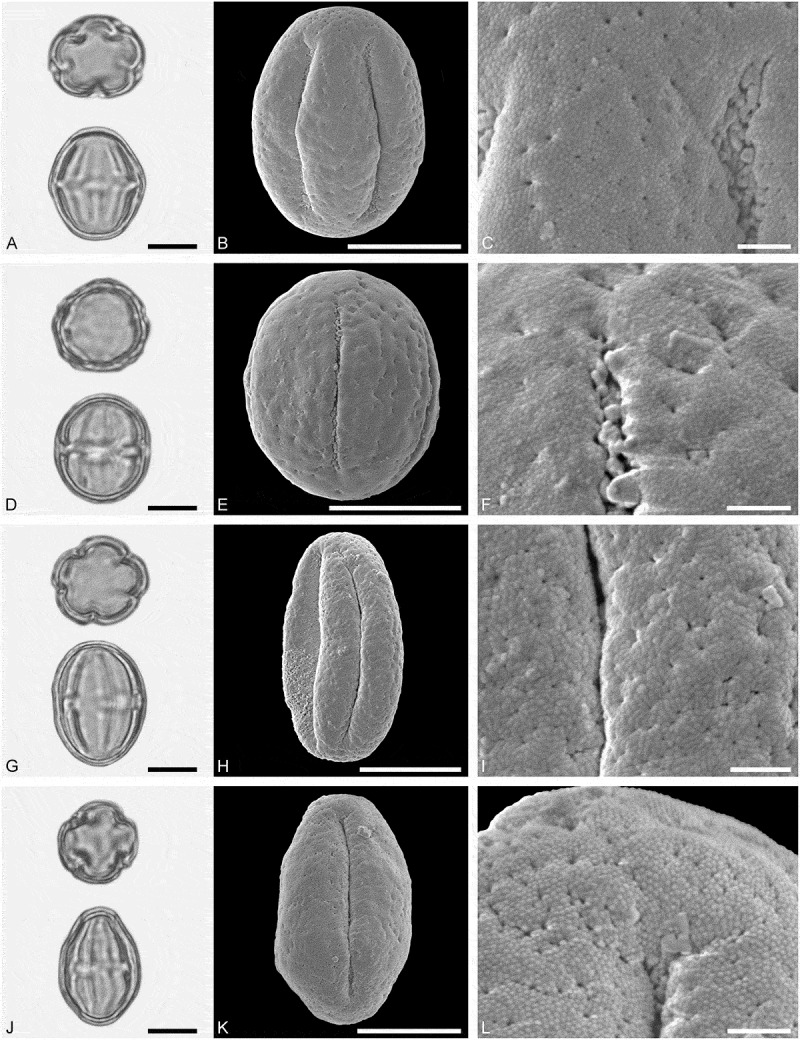
Light microscopy (LM) (**A, D, G, J**) and scanning electron microscopy (SEM) (**B, C, E, F, H, I, K, L**) micrographs of dispersed fossil Sapotaceae pollen. **A–C.** Sapotaceae gen. et spec. indet. 1, close-up showing microverrucate and perforate sculpture and widening at the end of colpi. **D–F.** Sapotaceae gen. et spec. indet. 1, close-up of colpus membrane. **G–I.** Sapotaceae gen. et spec. indet. 1, close-up of sculpture in area of mesocolpium. **J–L.** Sapotaceae gen. et spec. indet. 1, close-up of polar area. Scale bars 10 µm (A, B, D, E, G, H, J, K), 1 µm (C, F, I, L).

##### Description

Pollen, monad, prolate, outline convex-quadrangular to pentagonal in polar view, elliptic in equatorial view; polar axis 21–26 µm long in LM, 17–24 µm long in SEM, equatorial diameter 16–20 µm wide in LM, 13–16 µm wide in SEM; stephano(4–5)colporate, colpi narrow, varying in length, often wider and rounded or truncated at ends, endopori lalongate, margins of endopori perpendicular to polar axis thickened; exine 0.8–1.4 mm thick (LM), nexine as thick as sexine; tectate; sculpture psilate in LM, microverrucate, perforate, fossulate in SEM, microverrucae closely spaced, perforations and fossulae more frequent in polar areas, colpus membrane microverrucate (SEM).

##### Remarks

The pollen morphology (LM and SEM) and ultrastructure (TEM) from 48 of the 53 genera of Sapotaceae has been documented in a series of publications by Harley (1986a, 1986b, 1990, 1991a, 1991b, 2004). In the two main publications, Harley identified 12 pollen types (Harley 1991a), subdivided into a total of 49 subtypes (Harley 1991b). The Sapotaceae gen. et spec. indet. 1 and 2 pollen both fall within the range of ‘Pollen Type 1ʹ (Harley 1991a, figure 6) and ‘Subtype 1A’ (Harley 1991b, figures 5, 6) that occur in subfamilies Sapotoideae (*Madhuca, Manilkara, Mimusops, Palaquium*) and Chrysophylloideae (*Xantolis*). Since only a fraction of the taxa assigned to ‘Subtype 1A’ by Harley (1991b) are illustrated in both LM and SEM, a more detailed comparison and assignment of both fossil pollen types to particular extant genera is impractical.

##### Fossil record

The Sapotaceae pollen record has been summarised by Muller (1981), Harley (1991a, 1991b), Song et al. (2004), and Stuchlik et al. (2014). Few of the records date back to the late Cretaceous, but most are confined to the Cainozoic, suggesting a cosmopolitan distribution during the late Eocene. These fossil Sapotaceae pollen grains have been assigned to various species divided into two main form-genera, *Sapotaceoidaepollenites* R.Potonié, Thomson et Thiergart ex R.Potonié, and *Tetracolporopollenites* Pflug et Thomson. Most of these records are based on LM observations only but see Harley et al. (1991), and any affiliation to extant lineages and/or genera are uncertain (*cf.* Harley 1991b). Relying on the monumental work by Harley (1986a, 1986b, 1990, 1991a, 1991b, 2004) on the pollen morphology of extant Sapotaceae, it is clear that fossil pollen grains of this family can only be affiliated to extant lineages or genera using combined LM and SEM. Fossil pollen grains, documented via SEM, that are similar to the Sapotaceae gen. et spec. indet. 1 pollen type from Lavanttal, have been reported from the middle Eocene of Germany by Manchester et al. (2015, Sapotaceae gen. et spec. indet.) and Hofmann (2018, Mimusopeae/Isonandreae-type sp. 1 and sp. 2).

##### Ecological implications

All extant genera producing pollen similar to that of the Sapotaceae gen. et spec. indet. 1 and 2 pollen types are characterised by woody evergreen shrubs or trees. *Madhuca* comprises *c*. 100 species that occur from India through Malesia and south China and New Guinea (Pennington 1991, 2004). *Manilkara* is a pantropical genus composing *c*. 65 species occurring in the Americas (*c*. 30 spp.), Africa and Madagascar (*c*. 20 spp.), and in Asia and across the Pacific (*c*. 15 spp.) (Pennington 1991, 2004). *Mimusops* comprises *c*. 41 species that are distributed in Africa (*c*. 20 spp.), Madagascar (*c*. 15 spp.), the Mascarene Islands (4 spp.), the Seychelles, and in Asia and the Pacific (one species). The *c*. 110 spp. of *Palaquium* range from India through southeast Asia to the Pacific Islands. *Xantolis* is a small genus comprising *c*. 14 species ranging from southern India to Vietnam and southern China, with one species in the Philippines (Pennington 1991, 2004). All these genera are insect-pollinated and occur in tropical or/to subtropical regions, where they are part of various vegetation units. We speculate that the Sapotaceae gen. et spec. indet. 1 and 2. originate from shrubs or small trees that were part of the lowland wetlands or growing along streams in the surrounding hillside forests of the Lavanttal Basin.

**Figure 20.**
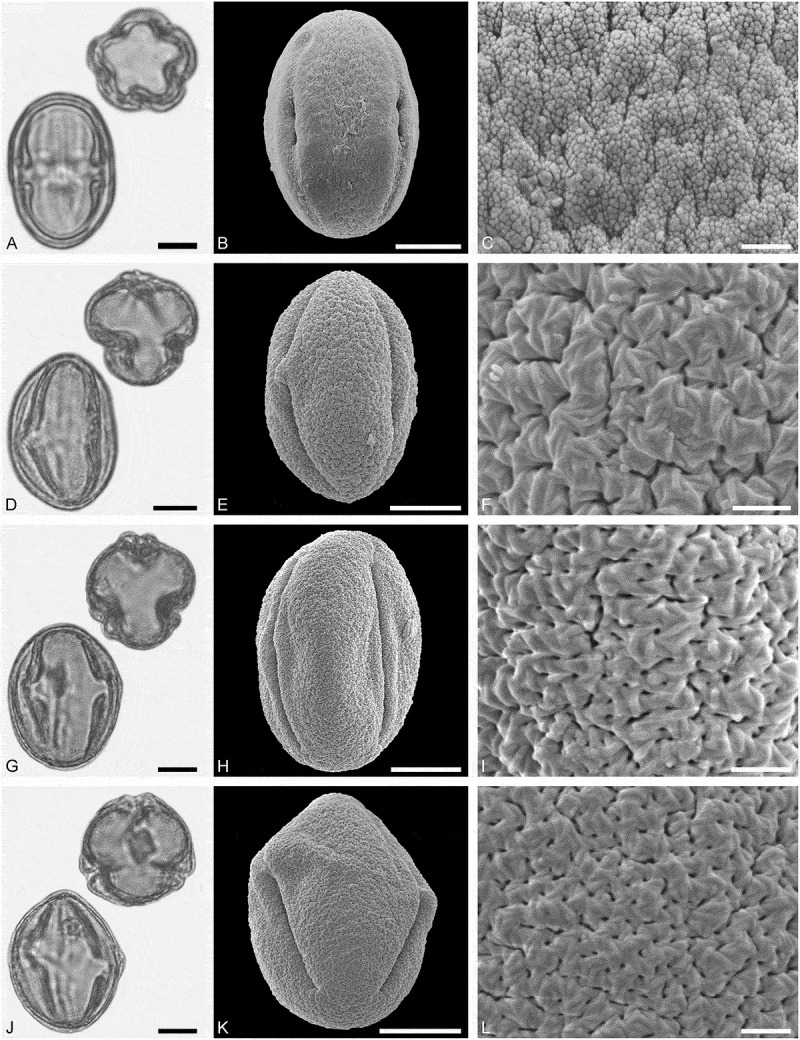
Light microscpy (LM) (**A, D, G, J**) and scanning electron microscopy (SEM) (**B, C, E, F, H, I, K, L**) micrographs of dispersed fossil Sapotaceae and Styracaceae pollen. **A–C.** Sapotaceae gen. et spec. indet. 2, close-up of sculpture in area of mesocolpium. **D–F.** *Rehderodendron* sp., close-up of rugulae composed of rod-like elements in area of mesocolpium. **G–I.** *Rehderodendron* sp., close-up of sculpture in area of mesocolpium. **J–L.** *Rehderodendron* sp., close-up of sculpture in area of mesocolpium. Scale bars 10 µm (A, B, D, E, G, H, J, K), 1 µm (C, F, I, L).

Sapotaceae *gen. et spec. indet. 2*

(*Figure 18G–L, 20A–C*)

##### Description

Pollen monad, prolate, outline quadrangular to pentangular in polar view, elliptic in equatorial view; polar axis 37–40 µm long in LM, 34–36 µm long in SEM equatorial diameter 27–32 µm wide in LM, 22–26 µm wide in SEM; stephano(4–5)colporate, colpi narrow, endopori lalongate elliptic, margins of endopori thickened; exine 1.9–2.5 µm thick, nexine thinner than sexine, sexine thickened along colpi (LM); tectate; sculpture scabrate in LM, slightly rugulate, microverrucate, perforate, fossulate in SEM, colpus membrane microverrucate (SEM).

##### Remarks

The Sapotaceae gen. et spec. indet. 2 pollen differs from the sp. 1 pollen type in both size and outline in polar view (square versus circular or lobed). The sp. 2 pollen type is also consistently tetra-aperturate, but the sp. 1 pollen is usually equipped with five apertures.

##### Fossil record

Comparable pollen, studied using SEM, has been describedearly Miocene/early Miocene of Altmittweida, Germany (as Sapotaceae gen. et sp. indet. 2 and 3, Kmenta & Zetter 2013), and the early and middle Miocene of Turkey (Bouchal et al. 2017; Bouchal 2019; Denk et al. 2019).

Family Styracaceae DC. et Spreng.

*Genus* Rehderodendron *Hu*

**Figure 21.**
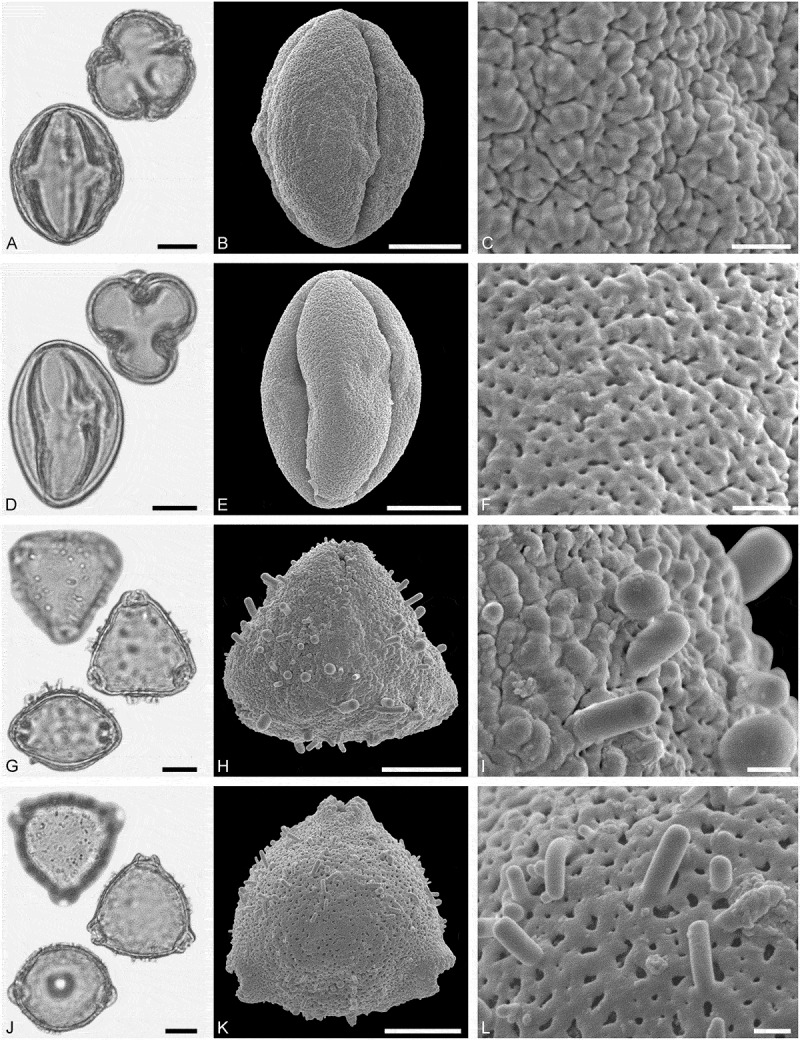
Light microscopy (LM) (**A, D, G, J**) and scanning electron microscopy (SEM) (**B, C, E, F, H, I, K, L**) micrographs of dispersed fossil Styracaceae and Symplocaceae pollen. **A–C.** *Rehderodendron* sp., close-up of sculpture in area of mesocolpium. **D–F.** *Rehderodendron* sp., close-up of sculpture in area of mesocolpium. **G–I.** *Symplocos* sp. 1, close-up of sculpture in polar area. **J–L.** *Symplocos* sp. 2, close-up of sculpturing in polar area. Scale bars 10 µm (A, B, D, E, G, H, J, K), 1 µm (C, F, I, L).

Rehderodendron *sp.*

(*Figure 20D–L, 21A–F*)

##### Description

Pollen, monad, prolate, outline lobate in polar view, elliptic in equatorial view; polar axis 35–38 µm long in LM, 30–35 µm long in SEM, equatorial diameter 24–29 µm wide in LM, 22–25 µm wide in SEM; tricolporate, colpi long, endopori large, lalongate, H-like in outline; exine 1.0–1.2 µm thick (LM), nexine thinner than sexine, nexine thickened along colpi, sexine protruding in area of endopori; sculpture scabrate in LM, rugulate to microrugulate, perforate in SEM, rugulae and microrugulae composed of rod-like elements, rod-like units often radiating from perforations, curved and/or interwoven, varying considerably in length, sometimes indistinct (SEM).

##### Remarks

**Figure 22.**
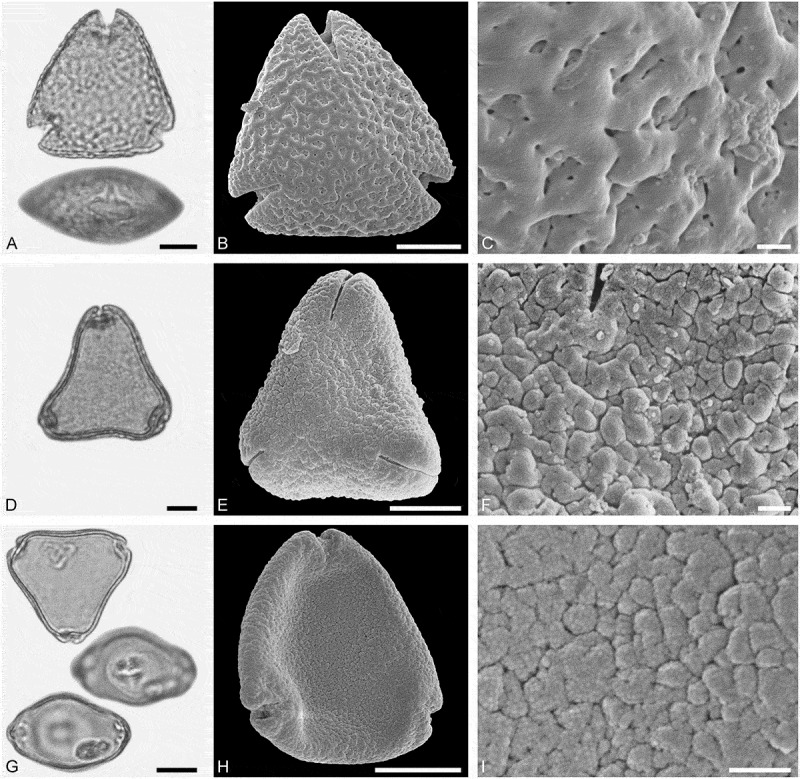
Light microscopy (LM) (**A, D, G**) and scanning electron microscopy (SEM) (**B, C, E, F, H, I**) micrographs of dispersed fossil Symplocaceae pollen. **A–C.** *Symplocos* sp. 3, close-up of sculpture in polar area. **D–F.** *Symplocos* sp. 4, close-up of sculpture in polar area. **G–I.** *Symplocos* sp. 5, close-up of sculpture in polar area. Scale bars 10 µm (A, B, D, E, G, H), 1 µm (C, F, I).

**Figure 23.**
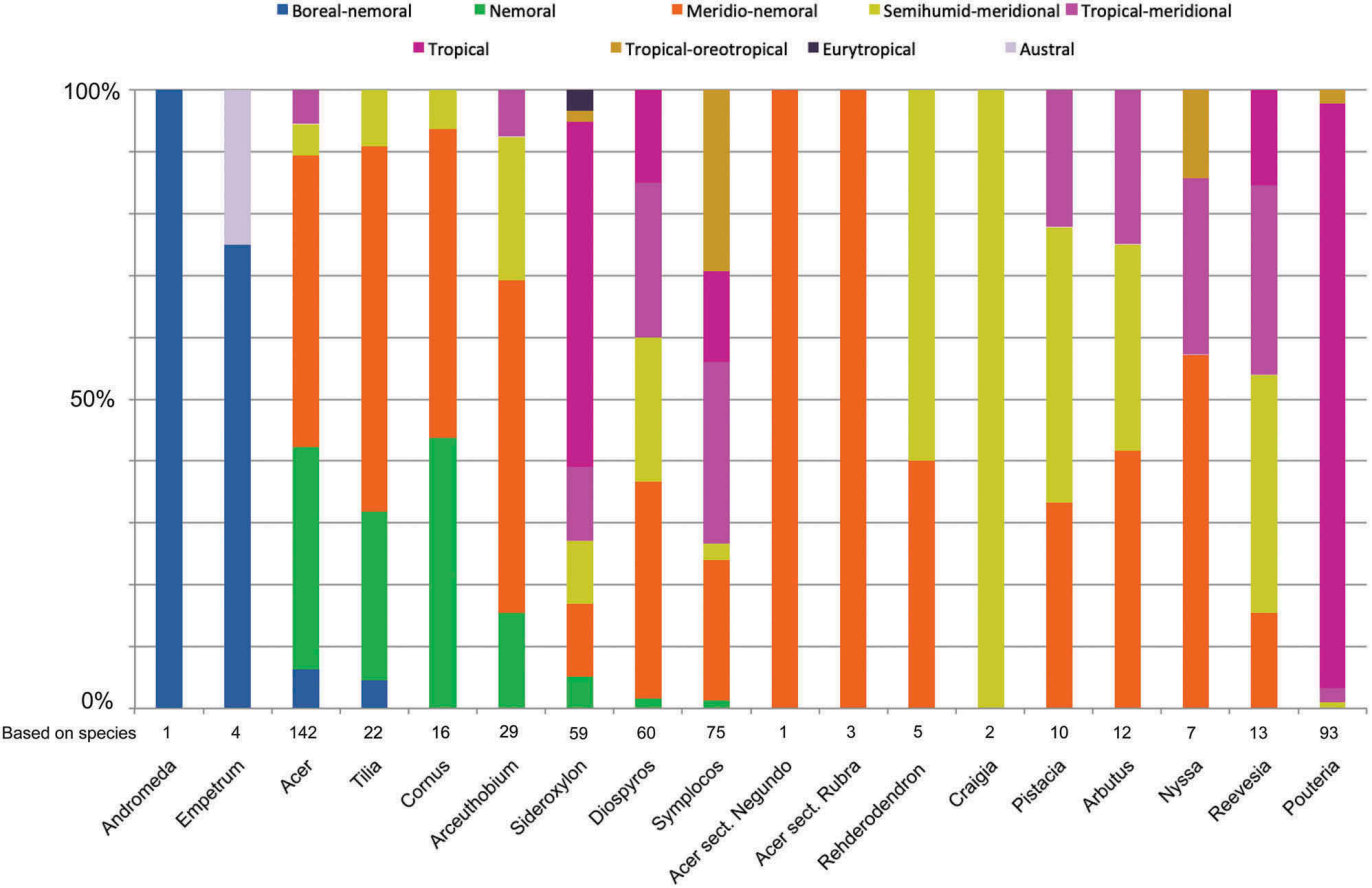
Köppen signatures of potential modern analogues of Myrtales, Malvales, Santanales and Ericales lineages found at the Lavanttal site. The bar chart shows the proportion of extant species part of the modern genus/lineage categorised for generalised climate–vegetation types (see Denk et al. 2013; Grímsson et al. 2016; see also Material and methods section). Boreal-nemoral elements preference for *D*-climates and *C*-climates, occurring in snow and temperate climates with hot to cool summers; nemoral elements preference for warm temperate and/or snow climates with warm summers (*Cfb, Cwb, Csb, Dfb, Dwb, Dsb*); meridio-nemoral elements preference for warm temperate climates with hot, but not warm, summers (*Cfa*- and *Cwa*-climates); semihumid-meridional elements preference for semihumid warm temperate climates with hot (and warm) summers; tropical-meridional elements preference for tropical (*A*-climates) and warm temperate climates with hot but not warm summers; tropical elements species restricted to tropical (*A*-climates); eurytropical elements preference for non-tropical climates with summer draught and generally dry climates (*B*- and *Cs*-climates); oreotropical elements species restricted to temperate climates along altitudinal successions within the tropical zone (*Cfa, Cfb, Cwa, Cwb*).

Pollen from three out of five *Rehderodendron* species has been studied using LM and SEM by Liang and Yu (1985) and Morton and Dickison (1992). The sculpture range, in the area of the mesocolpium, of the Lavanttal pollen is comparable to that documented by Liang and Yu (1985) for both *R. kweichowense* Hu and *R. macrocarpum* Hu (Liang & Yu 1985, plate 3, figures 22, 23).

##### Fossil record

The scarce macrofossil record of *Rehderodendron* is summarised by Mai (1970) and Manchester et al. (2009a). Fruits of this genus are documented from the early Eocene of UK (Mai 1970), the Miocene of Germany, Poland and Czech Republic (Mai 1970), the Pliocene of France (Geisser & Gregor 1981), Italy (Martinetto 1998) and Romania (Mai & Petrescu 1983). The fossil leaf records of Styracaceae are considered unreliable by Fritsch (2004) because they lack detailed anatomical features such as stellate or scale-like trichomes. Fossil pollen of *Rehderodendron*, identified using combined LM and SEM, has been reported from the Miocene of Germany (Ferguson et al. 1998) and Austria (Kovar-Eder et al. 1998).

##### Ecological implications

*Rehderodendron* is a small genus comprising five species of deciduous trees (up to 15 m tall) distributed in south-western China, Myanmar and Vietnam (Hwang & Grimes 1996; Fritsch 2004). The plants occur in dense forests, mixed broad-leaved evergreen and deciduous forests, at elevations from 100–1500 m (Hwang & Grimes 1996). All extant *Rehderodendron* species are currently growing under fully humid to winter-dry warm temperate climates with hot to warm summers (*Cfa, Cfb, Cwa, Cwb*; nemoral to meridio-nemoral vegetation element; File S1). Based on the current habitat of *Rehderodendron* the Lavanttal pollen may represent medium-sized trees that were part of the well-drained dense forests surrounding the basin.

Family Symplocaceae Desf.

*Genus* Symplocos *Jacq.*

Symplocos *sp. 1*

(*Figure 21G–I*)

##### Description

Pollen, monad, oblate, outline triangular in polar view, elliptic in equatorial view; polar axis 22–24 µm long in LM, equatorial diameter 31–33 µm wide in LM, 29–31 µm wide in SEM; brevitricolporate, colpi short, endopori lalongate; exine 1.2–1.4 µm thick (excl. echini), nexine slightly thinner or as thick as sexine (LM); tectate; sculpture baculate in LM, rugulate, verrucate, perforate, baculate in SEM, baculae irregularly distributed, varying in length and form (SEM).

##### Remarks

The pollen morphology (LM, SEM) and ultrastructure (TEM) of *Symplocos* has been studied by Erdtman (1952), van der Meijden (1970), Huang (1972), Barth (1979, 1982), Lieux (1982), Mai (1986), Nagamasu (1989a, 1989b), Premathilake et al. (1999), Aranha Filho et al. (2009), Li et al. (2011), Miyoshi et al. (2011), and Liu and Qin (2013). These studies show a considerable variation in pollen morphology, especially in sculpture observed with SEM. Some of these authors have pointed out characteristic pollen morphologies that are allegedly lineage-dependent and of systematic value (e.g. van der Meijden 1970; Barth 1979; Nagamasu 1989b). When pollen morphology of extant taxa is correlated to the newly established systematic framework by Fritsch et al. (2008), it is not obvious that pollen types are of diagnostic value below the genus level. The basal taxon, *S. paniculata* (subg. *Palura*), has a sort of reticulate to perforate sculpture in SEM (Nagamasu 1989b; Li et al. 2011); such sculpture is also found in pollen of many species of subg. *Symplocos* (sections *Lodhra* and *Hopea*: e.g. Nagamasu 1989b; Aranha Filho et al. 2009; Li et al. 2011; Miyoshi et al. 2011). Verrucate pollen occurs in other species of sect. *Lodhra* and *Hopea*, and baculate to echinate pollen in species of sect. *Lodhra, Hopea* and *Symplocos* (e.g. van der Meijden 1970; Barth 1979, 1983; Lieux 1982; Nagamasu 1989b; Aranha Filho et al. 2009; Li et al. 2011; Miyoshi et al. 2011; Liu & Qin 2013). Only the unique pollen types appear to be lineage-diagnostic, like that of *S. tinctoria* (see Lieux 1982; Nagamasu 1989a) or the latest diverged species of tropical America (see Bart 1982).

##### Fossil record

The fossil record of *Symplocos* was summarised among others by Krutzsch (1989), Mai and Martinetto (2006) and Tiffney et al. (2018). All authors agree that the leaf record is hard to judge because of their similarity to related genera. The fruit/seed fossil record is fairly rich. It includes finds from the middle Eocene of Oregon (Manchester 1994a) and the early Miocene of Vermont, USA (Tiffney et al. 2018), the Eocene to Pliocene of Germany (Mai & Martinetto 2006; Manchester & Fritsch 2014), the Oligocene to Miocene of Poland, Switzerland and Austria (e.g. Mai & Martinetto 2006), the Miocene of Czech Republic, France and Denmark, (e.g. Mai & Martinetto 2006; Manchester & Fritsch 2014), the Pliocene of the Netherlands, Italy (e.g. Mai & Martinetto 2006) and Japan (Miki 1937). The form-genera *Symplocoipollenites* R.Potonié and *Symplocospollenites* R.Potonié, Thomson et Thiergart are widely used for Symplocaceae pollen. The LM-based fossil pollen record of *Symplocos* is summarised by Muller (1981), Krutzsch (1989), Ivanov (1995, 2004) and Stuchlik et al. (2014). These include doubtful pollen from the Cretaceous of the Americas. The pollen record suggests that *Symplocos* already had a European-North American distribution during in Eocene. Based on the compiled fossil record, Manchester and Fritsch (2014) and Fritzsch et al. (2015) hypothesised an early Cainozoic European origin of the genus followed by an Eocene dispersal across the North Atlantic land bridge into the Americas. Dispersal from Europe into Asia is believed to have taken place following the closure of the Turgai Strait. There are only a few fossil *Symplocos* pollen grains that have been studied using combined LM and SEM. These include two pollen types from the Eocene of Profen, Germany (Haring 2014), three pollen types from the late Oligocene/early Miocene of Altmittweida, Germany (Kmenta 2011; Kmenta & Zetter 2013), five pollen types from the Miocene of Kreuzau, Germany (Ferguson et al. 1998, figured only one of the types), and numerous pollen types from the Miocene of Austria (Kovar-Eder et al. 1998, figured one out of three pollen types; Zetter 1998, figured none out of three pollen types; Meller et al. 1999, figured one out of five pollen types). Ashraf and Moosbrugger (1996) figured three types of *Symplocoipollenites* using SEM from the Lower Rhine Embament of Germany. Therefore, micrographs documenting the SEM-based sculpture variation in fossil *Symplocos* is lacking. Fossil pollen most similar to the Lavanttal fossils can be found in the Miocene (Ferguson et al. 1998, plate 6, figures 8–11), and, Eocene of Germany (Haring 2014, plates 8, 9).

##### Ecological implications

*Symplocos* comprises about 319 species distributed in tropical to subtropical regions of the Americas, eastern Asia and Australasia. These are mostly shrubs to small trees (< 20 m tall), but some become large trees that are up to 50 m tall. The plants are generally evergreen, but semi-deciduous (*S. tinctoria* [L.] L’Hér.) or deciduous (*S. paniculata* [Thung.] Miq.) species also exist (e.g. Fritsch et al. 2008; Kelly et al. 2016). The genus is divided into *Symplocos* subg. *Palura* (one species, *S. paniculata*, eastern Asia) and *Symplocos* subg. *Symplocos* (all other species, Americas, East Asia, Australasia). The latter subgenus is further divided into three sections, each showing a clear geographic pattern. Subgenus *Symplocos* sect. *Lodhra* comprises 142 species in eastern Asia and Australasia, *Symplocos* sect. *Hopea* (or *Barberina*) comprises 24 species in the Americas and one in eastern Asia, and *Symplocos* sect. *Symplocos* comprises 150 species in tropical America (Fritsch et al. 2008; Aranha Filho et al. 2009). Extant taxa producing pollen similar to the fossil *Symplocos* sp. 1 to sp. 4 occur in all three sections of *Symplocos* subgenus *Symplocos*, and show a subtropical to tropical distribution. The plants of *Symplocos* subg. *Symplocos* sect. *Lodhra*, growing in east to southeast Asia, are evergreen shrubs to small understory trees (mostly less than 10 m tall; rarely up to 30 m tall) occurring in mixed forests, especially on forested slopes, and depending on their geographic occurrence are found at elevations between 100 and 3000 m (Wu & Nooteboom 1996). They grow under equatorial monsoon and winter-dry savannah climates, as well as in fully humid to winter-dry warm temperate climates with hot or warm summers (*Am, Aw, Cfa, Cfb, Cwa, Cwb*; tropical-meridional to meridio-nemoral vegetation element; File S1). The Mexican to Central American *Symplocos* of subg. *Symplocos* sections *Symplocos* and *Hopea* (*S. culminicola* Standl. et Steyerm. and *S. longipes* Lundell) are also evergreen shrubs to small or medium-sized trees that are usually less than 20 m tall, and rarely up to 50 m in *S. hartwegii* A.DC. (Kelly et al. 2016). The plants occur mostly in tropical rainforests, from sea level up to 1600 m elevation, and in cloud forests, at elevations between 600 and 3350 m. They are also found in cool mountain forests, mountain rain forests, broad-leaved evergreen forests and mixed forests (Kelly et al. 2016). In this part of the world, *Symplocos* thrives under fully humid equatorial rainforest climate, equatorial monsoon climate, and summer-dry equatorial savannah climates (*Af, Am, Aw*; tropical vegetation element; File S1), but extends into fully humid or winter-dry warm temperate climates with warm summer (*Cfb, Cwb*). Based on the above, the various *Symplocos* pollen (sp. 1 to sp. 4) could represent evergreen shrubs or small trees that were part of the understory in the mixed deciduous-evergreen broad-leaved and conifer forests surrounding the basin.

Symplocos *sp. 2*

(*Figure 21J–L*)

##### Description

Pollen, monad, oblate, outline triangular in polar view, elliptic in equatorial view; polar axis 25–27 µm long in LM, equatorial diameter 32–34 µm wide in LM, 29–31 µm wide in SEM; brevitricolporate, colpi short, endopori lalongate, colpori annulate; exine (excl. echini) 1.2–1.3 µm thick (LM), nexine as thick as sexine, sexine protruding in area of endopori (LM); tectate; sculpture baculate in LM, foveolate, perforate, baculate in SEM, baculae irregularly distributed, varying in length, foveolae irregular in outline (SEM).

Symplocos *sp. 3*

(*Figure 22A–C*)

##### Description

Pollen, monad, oblate, outline triangular in polar view, elliptic in equatorial view; polar axis 20–22 µm long in LM, equatorial diameter 42–44 µm wide in LM, 37–39 µm wide in SEM; brevitricolporate, colpi short, endopori, elliptic lalongate, margin of endopori thickened; exine 1.4–1.6 µm thick, nexine as thick as sexine (LM); tectate; sculpture reticulate in LM, foveolate to reticulate, perforate in SEM, colpus membrane granulate (SEM).

##### Remarks

Fossil pollen similar to the *Symplocos* sp. 3 from Lavanttal has been figured by Haring (2014, plates 5–7) from the Eocene and by Kmenta and Zetter (2013, plate 6, figures 7–9) from the late Oligocene/early Miocene of Germany.

Symplocos *sp. 4*

(*Figure 22D–F*)

##### Description

Pollen, monad, oblate, outline triangular in polar view, elliptic in equatorial view; equatorial diameter 42–44 µm wide in LM, 34–36 µm wide in SEM; brevitricolporate, colpi short, endopori lalongate, margin of endopori thickened; exine 1.8–2.1 µm thick, nexine as thick as sexine (LM); tectate, sculpture scabrate in LM, verrucate to rugulate, perforate, fossulate in SEM, low relief sculpture around colpi (SEM).

##### Remarks

Fossil pollen similar to the *Symplocos* sp. 4 from Lavanttal has been figured by Kmenta and Zetter (2013, plate 7, figures 1–3) from the late Oligocene to early Miocene of Altmittweida, Germany.

Symplocos *sp. 5 (aff*. S. tinctoria)

(*Figure 22G–I*)

##### Description

Pollen, monad, oblate, outline triangular in polar view, elliptic in equatorial view; polar axis 18–21 µm long in LM, equatorial diameter 29–31 µm wide in LM, 27–29 µm wide in SEM; brevitricolporate, colpi short, endopori lalongate, margin of endopori thickened; exine 1.2–1.4 µm thick (LM), nexine as thick as sexine, sexine protruding in area of aperture; tectate, sculpture scabrate in LM, verrucate to microverrucate in SEM, verrucae and microverrucae with a nanoverrucate suprasculpture (SEM).

##### Remarks

This fossil *Symplocos* pollen type, with its nanoverrucate suprasculpture, is very similar to pollen of extant *S. tinctoria*, the only semi-deciduous taxon in the *Symplocos* subg. *Symplocos* sect. *Hopea* (or *Barberina*), figured by Nagamasu (1989a) and Lieux (1982). Fritsch et al. (2015) concluded that *Symplocos* originated in Eurasia and dispersed into the Americas during the early Cainozoic. They also postulated that dispersal from North America back to Eurasia occurred within *Symplocos* sect. *Hopea* in the middle to late Miocene, pinpointing *S. tinctoria* as one of two species involved in the Miocene disjunction. This fossil pollen type from the late middle Miocene of Lavanttal seems to collaborate that theory.

##### Ecological implications

*Symplocos tinctoria* is distributed in south-eastern United States, from sea level to an elevation of *c*. 1400 m. It is a deciduous shrub to small tree and part of several different vegetation units ranging from lowland wetlands to well-drained or dry upland forests (moist mixed-deciduous hardwoods to dry pine-oak woods). It occurs in maritime forests, swamps, hammocks, bottomlands, flatwoods, streamheads, baygalls, on rocky summits and in ravines (Almeda & Fritsch 2009). *Symplocos tinctoria* is growing under fully humid warm temperate climate with hot to warm summers (*Cfa, Cfb*; meridio-nemoral vegetation element; File S1). The fossil pollen could have originated from small understory trees growing either in the lowland wetlands or in forests on dryer substrates surrounding the Lavanttal Basin.

## Discussion

### Occurrence and identification of angiosperm pollen

In total, 46 angiosperm pollen types are described herein. Of these, we suspect that 16 types (35%) were previously identified from the Lavanttal Basin by Klaus (1984). Since Klaus studied most of the palynoflora using LM only, it is sometimes uncertain if the pollen types described/figured here represent the same taxon as depicted by Klaus (Table I; for example, *Tilia caroliniana*, Chenopodiaceae Form A, *Ericaceoipoll. cf. acastus*). Pollen of the genera *Craigia* (Malvaceae), *Pistacia* (Anacardiaceae), *Zanthoxylum* (Rutaceae), *Persicaria, Rumex* (Polygonaceae), *Alangium, Cornus* (Cornaceae), *Diospyros* (Ebenaceae), *Andromeda, Empetrum, Erica* (Ericaceae), *Pouteria, Sideroxylon* (Sapotaceae), and *Rehderodendron* (Styracaceae) are first-time reports. We further document a higher diversity within previously identified genera like *Ludwigia* (Onagraceae), *Tilia* (Malvaceae), *Acer* (Sapindaceae), and *Symplocos* (Symplocaceae). Quantitatively, the pollen types described here rarely make up for more than 0.5% of the entire palynomorph spectrum (F. Grímsson, pers. comm., based on six years of studying this sample). The Malvaceae (*Craigia, Reevesia, Tilia*) and the Sapindaceae (*Acer* sp. 1 and sp. 3) are by far the most common types. Followed by *Nyssa* (Cornaceae), *Arceuthobium* (Santalaceae), *Arbutus, Erica* sp. 2 and sp. 5, Ericaceae gen. et spec. indet. 1 (Ericaceae), and Sapotaceae gen. et spec. indet. 1 and 2 (Sapotaceae). All other pollen types presented herein are extremely rare and would most likely not be encountered in a conventional LM observation that includes counting up to 600 grains.

### Palaeoecological interpretations and palaeoenvironmental reconstructions preliminary results

Previous contributions on the middle Miocene (Sarmatian) palynoflora from the Lavanttal Basin suggest that pollen and spores, preserved in freshwater lake sediments, originate from both lowland wetland plants, as well as plants that occurred in various vegetation units of the surrounding hills/mountains (Grímsson et al. 2011, 2015a, 2016a; Grímsson & Zetter 2011). Based on the ecological preferences of potential modern analogues of the fossil pollen/spores from the Lavanttal Basin, we suggest that the pollen described in this part dispersed from plants occurring in different habitats/vegetation units in lowland wetlands, in dryer lowlands at the periphery of the basin, and in the surrounding highlands and mountains (Table II).

Taxa typical of lowland wetland forests (mixed evergreen/deciduous broad-leaved/conifer forests), occupying lake margins, banks of rivers, swamps, levees, hammocks, floodplains, etc. found at the Lavanttal site include: *Acer, Alnus*, Amaranthaceae, *Andromeda, Arceuthobium* (host-dependent, parasite on conifers), *Betula, Carex, Carpinus, Carya, Cedrelospermum, Celtis, Cercidiphyllum, Cornus, Craigia, Diospyros, Dryopteris, Elaeagnus, Ephedra, Erica*, Ericaceae, *Ginkgo, Glyptostrobus, Juglans, Larix, Liquidambar, Ludwigia, Lycopodium, Magnolia, Myrica*/*Morella, Nyssa, Osmunda, Ostrya, Parthenocissus, Persicaria, Picea, Pinus, Platanus, Pouteria, Prunus, Pterocarya, Quercus* subg. *Quercus* (mainly sects *Lobatae, Virentes*), Ranunculaceae, *Rumex, Salix*, Sapotaceae, *Sciadopitys, Selaginella, Sequoia, Sideroxylon, Sparganium, Sphagnum, Symplocos, Trigonobalanopsis, Typha, Ulmus, Vitis*, and *Zanthoxylum*.

Many taxa like *Acer, Alangium*, Amaranthaceae, *Arbutus, Betula*, Caryophyllaceae, *Cornus, Corylus*, Diospyros, *Elaeagnus*, Engelhardioideae, *Erica*, Ericaceae, *Myrica*/*Morella, Nyssa, Quercus* sect. *Cyclobalanopsis, Persicaria, Pistacia, Pouteria, Prunus, Pterocarya, Reevesia, Rehderodendron*, Rhamnaceae, *Rumex*, Sapotaceae, *Sideroxylon, Symplocos, Tilia, Zanthoxylum*, and *Zelkova* were most likely (also) growing in lowland/hinterland forests along or outside the periphery of the wetland basin and reaching into hillside forests surrounding the basin. Some were probably also growing along streams reaching well into the surrounding highland and/or mountains.

At some distance from the main accumulating area, occurring in the mixed hillside and mountain forest, were *Abies, Buxus, Carya, Castanea, Cathaya, Cedrus, Celtis, Cercidiphyllum, Cryptomeria, Daphnyphyllum, Distylium, Elaeagnus*, Engelhardioidea, *Fortunearia, Ginkgo, Juglans, Keteleeria, Larix, Lycopodium, Myrica*/*Morella, Ostrya, Parrotia, Parthenocissus, Picea, Pinus, Prunus, Pteris, Quercus* (sects *Cerris, Ilex, Lobatae, Quercus*), *Sequoia, Trochodendron, Tsuga, Ulmus, Vitis*, and *Zelkova*.

Based on the dominant climatic preferences of potential modern analogues of the fossil Myrtales to Ericales from the Sarmatian of Lavanttal, as expressed by their ‘Köppen signatures’ (Figure 23, Table III), equatorial (*A* [lowlands]), arid (B), and polar (*E* [at highest altitudes]) climates seem unlikely for this region at that time. Even though some of the taxa, including *Reevesia, Pistacia, Zanthoxylum, Nyssa, Dyospyros, Arbutus, Erica*, and *Symplocus*, extend into equatorial climates, they also thrive in various warm temperate (*C*) climates, while by far the most elements are extra-tropical. The majority of the woody plants, *Craigia, Reevesia, Tilia, Pistacia, Zanthoxylum, Acer, Alangium, Cornus, Nyssa, Diospyros, Arbutus, Erica, Rehderodendron*, and *Symplocos*, are mostly nemoral and/or meridio-nemoral vegetation elements and typical of warm temperate (*C*) climates (Figure 23, Table III). *Nyssa talamancana* thrives at middle elevations in Costa Rica and Panama under fully humid equatorial climate or fully humid warm temperate climate with warm summers (*Af, Cfb*; tropical-oreotropical vegetation element; File S1) together with other relict genera, e.g. *Ticodendron, Alfaroa, Oremunnea* and *Gordonia* (Hammel & Zamora 1990). Sapotaceae, which are warmth-loving and predominantly occur under equatorial (*A*) climates, also extend into warm temperate (e.g. *Cwa, Cwb, Cfa, Cfb*) climates. The co-occurrence of insect-pollinated tropical-meridional (frost- and snow-cover intolerant) and meridional-nemoral (extra-tropical) elements and the scarcity of steppe-climate (*BS*) tolerating taxa points towards a subtropical, humid warm temperate climate with hot summers (*Cfa, Cwa*) for the lowland deposition area. The taxa extending into snow (D) and polar (*E*) climates (Amaranthaceae, Caryophyllaceae, *Andromeda, Empetrum*, Ericaceae, *Ludwigia*) are water plants and/or herbaceous plants or small shrubs with a cosmopolitan distribution, and therefore more or less climate independent.

**Table III.** Summarised ‘Köppen signatures’ for Myrtales, Sapindales, Santalales, Caryophyllales, Cornales and Ericales of Lavanttal.

	Pollen taxon/taxa	Köppen signature	Indicative of
O	*Ludwigia* sp. 1/2	Cosmopolitan	Cosmopolitan
M	*Craigia* sp.	***Cwa***	Winter dry warm temperate climate with hot summer
M	*Reevesia* sp.	*Aw, Cfa, **Cwa***	Winter dry warm temperate climate with hot summer
M	*Tilia* sp. 1/2	***Cfa***, *Cfb, **Cwa**, Cwb, Dwa, Dwb, Dfa, Dfb*	Fully humid to winter dry warm temperate climate with hot summer
AN	*Pistacia* sp.	*Aw, **Cfa***, ***Cwa***, *Cwb*	Fully humid to winter dry warm temperate climate with hot summer
R	*Zanthoxylum* sp. 1/2	*Aw, **Cfa, Cwa, Cwb**, Dwa*	Fully humid to winter dry warm temperate climate with hot to warm summer
SA	*Acer* sp. 1/4	***Cfa, Cfb***, ***Dfa***, ***Dfb***, *Cwa, Dwa, Csb*	Fully humid warm temperate to snow climate with hot to warm summer
SN	*Archeuthobium* sp.	*B*, ***Cs, Cw***, ***Ds, Dw, Df***	Summer to winter dry warm temperate to snow climate, fully humid snow climate, or arid climates
AM	Amaranthaceae gen. et spec. indet. 1/2	Cosmopolitan	Cosmopolitan
CA	Caryophyllaceae gen. et spec. indet. 1/2/3	Cosmopolitan	Cosmopolitan
P	*Persicaria* sp.	Cosmopolitan	Cosmopolitan
P	*Rumex* sp.	Cosmopolitan	Cosmopolitan
CO	*Alangium* sp.	***Cfa***, *Cwa*	Fully humid to winter dry warm temperate climate with hot summers
CO	*Cornus* sp.	***Cfa, Cfb, Dfa, Dfb***, *Dfc, **Cwa, Cwb**, Dwa, Dwb, Dwc, Csa, Csb*,	Fully humid to winter dry warm temperate to snow climate with hot to warm summer
CO	*Nyssa* sp.	***Af***, *As, **Aw***, ***Cfa, Cfb***, *Dfa, Dfb, **Cwa***, ***Cwb***,	Fully humid to winter dry warm temperate climate with hot to warm summer and various equatorial climates
EB	*Diospyros* sp.	*Af, Am, **Aw, Cfa**, Cfb, **Cwa**, Cwb*	Fully humid to winter dry warm temperate climate with hot to warm summer and various equatorial climates
ER	*Andromeda* sp.	*Cfa, **Cfb, Cfc***, ***Dfb***, ***Dfc**, Dwb, Dwc, Dsb, Dsc, ET*	Fully humid warm temperate to snow climate with hot to warm summer and summer to winter dry snow climates
ER	*Arbutus* sp.	*Aw, BSh, BSk, **Csa***, ***Cwa***, ***Cwb, Csb, Cfa, Cfb**, Cfc*	Warm temperate climates
ER	*Empetrum* sp.	*Csb, Cfa, **Cfb, Cfc**, Dsc, Dwb, Dwc, **Dfa, Dfb, Dfc**, ET*	Fully humid warm temperate to snow climates with hot to cool summer and cold winter
ER	*Erica* sp. 1/2/3/4/5	*A, B, **Csa***, ***Csb, Cwa***, ***Cwb, Cfa***, ***Cfb***, ***Dfb, Dfc***	Warm temperate climates and fully humid snow climates with warm to cool summer and cold winter
ER	Ericaceae gen. et spec. indet. 1/2	Cosmopolitan	Cosmopolitan
SP	Sapotaceae gen. et spec. indet.1/2	***A***, *B, Cfa, Cfb, Cwa, Csa*,	Equatorial to warm temperate climate
SP	*Pouteria* sp. 1/2	***A***, *B, Cfa, Cfb, Cwa, Csa*,	Equatorial to warm temperate climate
SP	*Sideroxylon* sp.	***A***, *B, Cfa, Cfb, Cwa, Csa, Csb*	Equatorial to warm temperate climate
ST	*Rehderodendron* sp.	***Cfa***, *Cfb, **Cwa**, Cwb*,	Fully humid to winter dry warm temperate climate with hot to warm summer
SY	*Symplocos* sp. 1/2/3/4	***Af, Am, Aw, Cfa, Cfb, Cwa, Cwb***	Equatorial to warm temperate climate
SY	*Symplocos* sp. 5 (aff. *S. tinctoria*)	***Cfa***, *Cfb*	Fully humid warm temperate climate with hot to warm summer

Note: O, Onagraceae; M, Malvaceae; AN, Anacardiaceae; R, Rutaceae; SA, Sapindaceae; SN, Santalaceae; AM, Amaranthaceae; CA, Caryophyllaceae; P, Polygonaceae; CO, Cornaceae; EB, Ebenaceae; ER, Ericaceae; SP, Sapotaceae; ST, Styracaceae; SY, Symplocaceae. Most prominent climate types appear in bold. See Kottek et al. (2006) for the three letter code used in the Köppen-Geiger climate classification and quantitative definition of categories.

The climatic preferences of the taxa presented herein support our previously interpreted climate signal based on the fossil Fagales to Rosales from the Sarmatian of the Lavanttal Basin (Grímsson et al. 2016, figure 23, table III). The lowlands in the Lavanttal region probably thrived in fully humid warm temperate (*Cfa*) climate, with possible dryer winters than summers (→ *Cw* climate), subtropical conditions as found today in south-eastern part of the United States and southern China (see also Grímsson et al. 2016, figure 1). From the lowlands up into the mountains, subsequent altitudinal succession would have occurred, and the climate gradually shifted from warm temperate into snow (*D*) climates (*Cfa* → *Cfb*/*Dfa* → *Dfb*) providing niches for species intolerant to summer heat.

The angiosperm pollen described herein as well as palynomorphs previously presented by Grímsson et al. (2011, 2015a, 2016a) and Grímsson and Zetter (2011) represent the major part of the palynoflora from the Sarmatian of the Lavanttal Basin. The remaining angiosperm (and unknown) pollen types will be described in a following contribution. A detailed interpretation and comprehensive discussion of the palaeovegetation, ecology and paleoclimate is envisaged to be presented after the remaining pollen types have been described.
